# Redox mechanisms in hepatic chronic wound healing and fibrogenesis

**DOI:** 10.1186/1755-1536-1-5

**Published:** 2008-10-13

**Authors:** Erica Novo, Maurizio Parola

**Affiliations:** 1 Dipartimento di Medicina e Oncologia Sperimentale and Centro Interuniversitario di Fisiopatologia Epatica, Università degli Studi di Torino, Corso Raffaello 30, 10125 Torino, Italy

## Abstract

Reactive oxygen species (ROS) generated within cells or, more generally, in a tissue environment, may easily turn into a source of cell and tissue injury. Aerobic organisms have developed evolutionarily conserved mechanisms and strategies to carefully control the generation of ROS and other oxidative stress-related radical or non-radical reactive intermediates (that is, to maintain redox homeostasis), as well as to 'make use' of these molecules under physiological conditions as tools to modulate signal transduction, gene expression and cellular functional responses (that is, redox signalling). However, a derangement in redox homeostasis, resulting in sustained levels of oxidative stress and related mediators, can play a significant role in the pathogenesis of major human diseases characterized by chronic inflammation, chronic activation of wound healing and tissue fibrogenesis. This review has been designed to first offer a critical introduction to current knowledge in the field of redox research in order to introduce readers to the complexity of redox signalling and redox homeostasis. This will include ready-to-use key information and concepts on ROS, free radicals and oxidative stress-related reactive intermediates and reactions, sources of ROS in mammalian cells and tissues, antioxidant defences, redox sensors and, more generally, the major principles of redox signalling and redox-dependent transcriptional regulation of mammalian cells. This information will serve as a basis of knowledge to introduce the role of ROS and other oxidative stress-related intermediates in contributing to essential events, such as the induction of cell death, the perpetuation of chronic inflammatory responses, fibrogenesis and much more, with a major focus on hepatic chronic wound healing and liver fibrogenesis.

## Background

### From oxidative stress to redox homeostasis and redox signalling

**Figure 1:**
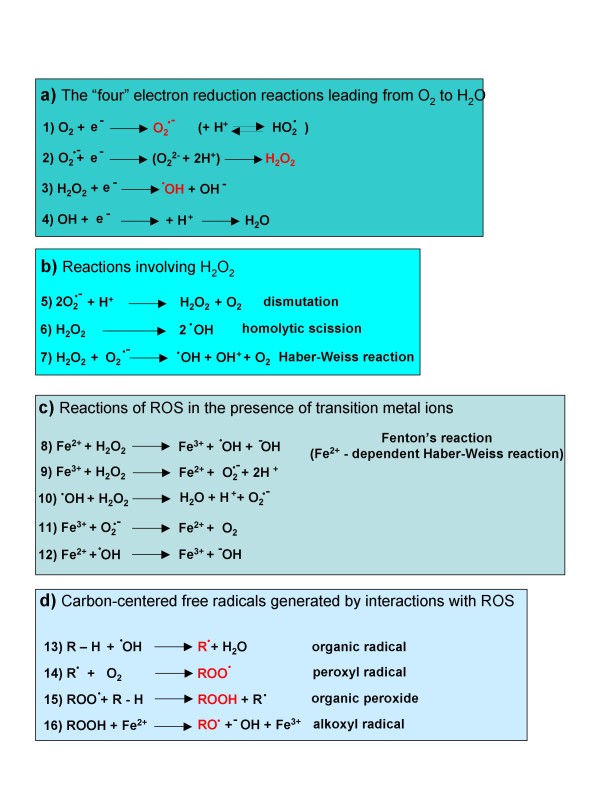
ROS are generated in biological systems through a number of interrelated reactions.

Molecular oxygen (O_2_) is essential for the survival of human beings and, more generally, of all aerobic organisms. Aerobic energy metabolism relies on oxidative phosphorylation, a crucial process by which the oxido-reduction energy of mitochondrial electron transport is eventually converted to the high-energy phosphate bond of ATP. Aerobic organisms use O_2 _as the final electron acceptor for mitochondrial cytochrome c oxidase, which, in turn, represents the terminal functional element of the mitochondrial multicomponent NADH dehydrogenase enzymatic complex, which is able to catalyze the four-electron reduction of O_2_, leading then also to H_2_O formation (Figure 1). During mitochondrial oxidative phosphorylation and other electron transfer reactions, however, partially reduced and highly reactive O_2 _metabolites, including superoxide anion (O_2_^•-^), hydrogen peroxide (H_2_O_2_) and hydroxyl radical (^•^OH), can be formed within cells. These reactive O_2 _metabolites are usually collectively referred to as 'reactive oxygen species' (ROS) and their generation in a biological environment exposes most living organisms to the so-called 'oxygen paradox': oxygen is necessary for life but it is also potentially hazardous since ROS may easily become a source of cell and tissue injury, as was recognized by early pioneers of free radical research [1-9]. However, as a natural consequence of this paradox, aerobic organisms have developed evolutionarily conserved mechanisms and strategies to carefully control the generation of ROS and other oxidative stress-related radical or non-radical reactive intermediates (that is, to maintain redox homeostasis), as well as to 'make use' of these molecules under physiological conditions as tools to modulate signal transduction, gene expression and cellular functional responses (the concept of redox signalling) [10-23].

At present, redox research is at the forefront of biomedical research in view of the expanding knowledge on the roles that increased and/or sustained levels of oxidative stress and related mediators have been described to play in major human diseases, including atherosclerosis, diabetes and cardiovascular diseases [14,24-29], cancer [30,31], neurodegenerative disorders [32-34], chronic liver [35-38] and lung diseases [39-41], to name just a few. Most of the conditions in which the role of oxidative stress and related mediators has been characterized belong to what one may define as chronic inflammatory/fibrogenic diseases, often involving chronic activation of wound healing.

### About this review

This review has been designed as an attempt to offer a comprehensive, but not hyper-specialized, critical introduction to current knowledge in the field in order to introduce readers to the fascinating complexity of redox signalling and redox homeostasis regulation, with a major focus on chronic wound healing and liver fibrogenesis. This review will then offer a sequence of ready-to-use key information and concepts on major types of ROS, free radicals and oxidative stress-related reactive intermediates operating in living organisms, their sources in mammalian cells and tissues, and antioxidant defences. Along these lines, this review will not intentionally deal with all the details but, whenever possible, the interested reader will find indications for highly recommended and more detailed and specialized reviews and articles on specific topics.

## ROS, free radical and non-radical reactive intermediates in biological materials

### Reactive oxygen species

**Figure 2:**
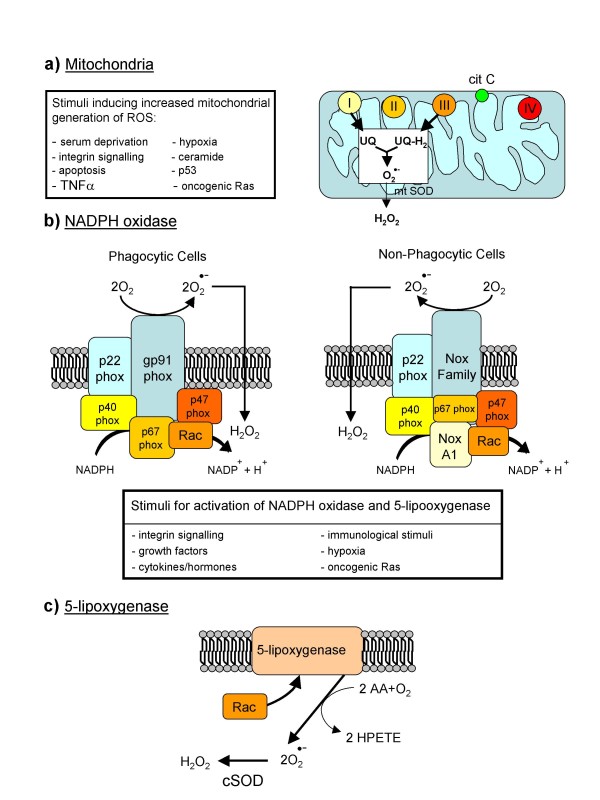
Major cellular sources of ROS in living cells.

ROS is a generic collective term indicating a number of active and reactive partially reduced O_2 _metabolites. Some of them, such as O_2_^•- ^and ^•^OH, can be defined as true free radicals, which are reactive molecular species with an impaired electron in their outer orbital. Free radicals are paramagnetic and reactive chemical entities that can undergo redox reactions by interacting with surrounding molecules in order to regain the more stable non-radical condition. Other ROS, such as H_2_O_2_, are more properly pro-oxidant non-radical agents. Indeed, O_2_^•-^, ^•^OH and H_2_O_2 _are by far the most relevant in physiological or pathophysiological conditions. Crucial information on major ROS, their sources and reactions are briefly summarized below and in Figures 1 and 2.

#### The superoxide anion O_2_^•-^

O_2_^•- ^is the result of univalent reduction of triplet state molecular oxygen; its intracellular generation can primarily occur non-enzymatically by the intervention of redox components such as the semi-ubiquinone compound of the mitochondrial electron transport chain [8,42] or through the intervention of enzymes like NADPH-oxidase (NOX) [43,44], xanthine-oxidase and others (Figure 2; and see below) or in auto-oxidation reactions [7,8,42]. Major features of O_2_^•- ^include: it is a relatively unreactive intermediate, being able to act at best as a mild reactant in physiological conditions and, indeed, only the interaction with nitric oxide (NO) to give peroxynitrite is able to transform superoxide into a very reactive intermediate; in living tissue, O_2_^•- ^can be converted into H_2_O_2 _enzymatically by superoxide-dismutase (SOD) isoforms [42], or non-enzymically [8]; and it has a rather poor ability to cross biological membranes [42].

#### Hydrogen peroxide

H_2_O_2 _represents a two-electron reduction state of molecular oxygen and originates mainly from enzymatic dismutation catalysed by superoxide dismutase (SOD) isoforms. H_2_O_2 _can also originate from non-enzymic dismutation of O_2_^•- ^as well as from direct reduction of O_2_[8,42]. Major features of H_2_O_2 _include: it can easily diffuse across biological membranes; it is a non-radical potent oxidizing agent; in aqueous solutions it can oxidize or reduce several inorganic ions [42,45]; it can usually be removed by either catalase or glutathione peroxidase; it can give rise to the very reactive and damaging ^•^OH when interacting with O_2_^•- ^(Figure 1; Haber-Weiss reaction), or in the presence of divalent metal ions like iron and copper – when Fe^2+ ^is present, the latter reaction is also defined as Fenton's reaction (or Fe^2+^-catalysed Haber-Weiss reaction) [42]; and myeloperoxidase of phagocytic cells use it to form hypochlorite (HOCl), a highly reactive compound able to oxidize thiol groups, amino groups and methionine in proteins.

#### Hydroxyl radical

^•^OH is a three-electron reduction state of O_2 _formed during Haber-Weiss or Fenton reactions or by decomposition of peroxynitrite. ^•^OH has a very short half-life (10^-9 ^s) and high reactivity. As such, in biological systems it does not diffuse from the site of generation and can rapidly damage any surrounding macromolecules, including: amino acids, potentially leading to protein inactivation/denaturation; carbohydrates, with degradation; lipids, leading to lipid peroxidation; and nucleic acids, leading, for example, to the formation of adducts with deoxyguanidine (8-OH-dG adducts, a reliable marker of ROS-dependent damage to DNA) and, potentially, to mutations.

#### Major reactions of ROS and other related free radicals

Figure 1 offers a summary of most relevant reactions leading to the generation of ROS or to their transformation into other reactive intermediates or inactive molecules. Reactions 1 to 12 have been already described, so we mention here only reactions 13 to 16. Reaction 13 is a 'starting' reaction, leading to the generation of an organic radical, R•, as a consequence of the interaction of ^•^OH with an organic carbon-hydrogen bond. Peroxides, peroxyl-radicals as well as alkoxyl-radicals (reactions 14 to 16) can be generated during on-going oxidative stress as, for example, during lipid peroxidation.

#### Intracellular sources of ROS

In living cells ROS can be generated by several sources, but without any doubt the most relevant are those described in Figure 2.

#### Mitochondria

Approximately 1–5% of electrons 'flowing' through the electron transport chain can be diverted to form O_2_^•- ^at the levels of complex I (NADH/ubiquinone oxidoreductase) and complex III (ubiquinol/cytochrome c oxidoreductase). O_2_^•- ^is then usually converted by mitochondrial SOD into H_2_O_2_, which can cross mitochondrial membranes to reach the cytoplasm [8,42].

#### NADPH oxidase

NOX is present in both professional phagocytic cells (macrophages, neutrophils and eosinophils) and non-phagocytic cells and plays a crucial role in different diseases [43,44,46], including chronic liver diseases (CLDs) [47,48]. The classic phagocytic NOX is formed by the two membrane bound components p22phox and gp91phox/Nox2 (comprising the flavocytochrome b558) and four cytosolic components (p40 phox, p47phox, p67phox and the GTPase Rac1/2), which, following stimulation of phagocytic cells, are recruited to the plasma membrane where they interact with Cyt b558, leading to increased activity and then ROS generation. The NOX of non-phagocytic cells is similar in structure and function, with gp91phox/Nox2 being replaced by another member of the same family of proteins (usually by Nox2 homologues Nox1, Nox3, Nox4, Nox5 or Duox1/2). The main difference, relevant for redox signalling, is that non-phagocytic NOX is constitutively active, producing a very low level of ROS and increasing both its activity and ROS generation in response to a number of factors and conditions.

#### 5-Lipoxygenase

5-Lipoxygenase (5-LOX) is a mixed function oxidase involved in the synthesis of leukotrienes from arachidonic acid in response to essentially the same stimuli that are able to stimulate NOX, particularly growth factors and cytokines. The latter mediators lead to membrane ruffling and the generation of superoxide, and then H_2_O_2_, through the intervention of the small GTPase Rac1 and a SOD isoform [14-18,49].

#### Other enzymes

ROS can also be generated enzymatically in many subcellular compartments by several oxidases, peroxidases, and mono- and di-oxygenases as well as by isoforms of the cytochrome P450 superfamily. Here it seems relevant to mention xanthine oxidase [42,50], nitric oxide synthase [51], cyclooxygenase [42,52] and other NAD(P)H dependent oxido-reductases, which are all able to generate primarily O_2_^•-^. Similarly, peroxisomal oxidases [52] (glycolate oxidases, D-amino oxidases, ureate oxidases, fatty acid-CoA oxidases and L-α-hydroxyacid oxidases) can generate H_2_O_2 _when metabolizing various substrates. Also, lysyl oxidase [52], the enzyme catalysing the formation of the aldehyde precursors of cross-links in collagen and elastin, can give rise to H_2_O_2_.

### The process of lipid peroxidation and the generation of non-radical intermediates

**Figure 3:**
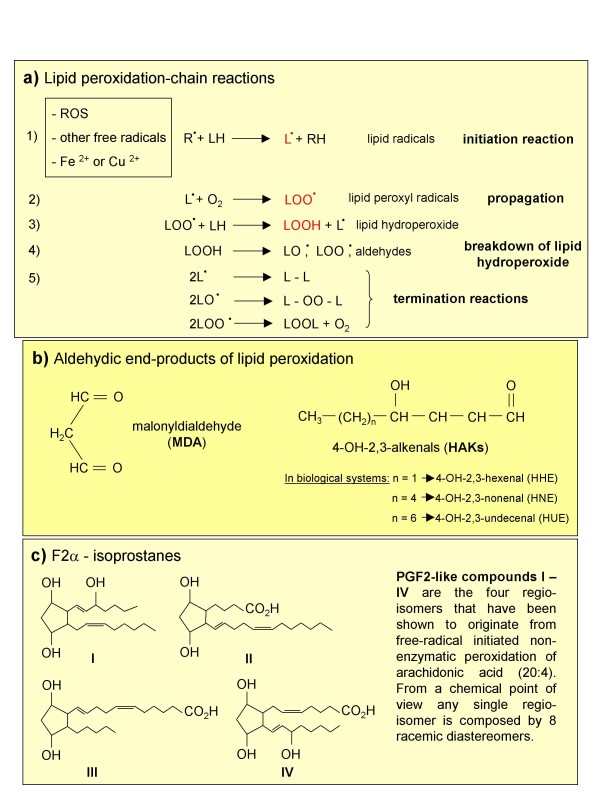
Lipid peroxidation and the formation of non-radical intermediates.

Lipid peroxidation (Figure 3) is a term commonly used to indicate oxidative decomposition of the ω-3 (22:6) and ω-6 (18:2, 20:4) polyunsaturated fatty acids of membrane phospholipids [19,42]. This process, which is very common in pathological conditions [21-23,42], is usually initiated by the interaction of a ROS or other free radical with polyunsaturated fatty acids and exacerbated by the presence of divalent metal ions. This reaction leads to the formation of lipid radicals (L•) that, in turn, can react with available O_2 _to generate lipid peroxyl radicals (LOO•). From this point the propagation phase of this chain reaction occurs, whereby LOO• interacts with other lipid molecules, resulting in the generation of lipid hydroperoxides (LOOH). These in turn undergo a degradative breakdown, leading to the generation of other radical species (LO• and LOO•) that further propagate lipid peroxidation, and to several aldehydic end-products, such as malonyldialdehyde (MDA) and 4-hydroxy-2,3-alkenals (HAKs) of different chain lengths, [19,42] as well as to F_2_-isoprostanes [53]. 4-Hydroxy-2,3-nonenal (HNE), the most active biological and pathophysiological HAK [23-25], and F_2_-isoprostanes (so defined because of their PGF_2_-like structure) are relatively stable and lipid soluble compounds that can diffuse from the site of generation and easily cross biological membranes. Moreover, as proposed more than 25 years ago for HNE [23-25,35,54] and more recently for F_2_-isoprostanes [55,56], these non-radical species can also act as mediators that are able to affect redox state, signal transduction and cell responses. Detection of HNE or F_2_-isoprostanes in biological fluids or tissues is today considered one of the best ways to evaluate *in vivo *on-going oxidative stress [57].

### Nitric oxide and reactive nitrogen species

**Figure 4:**
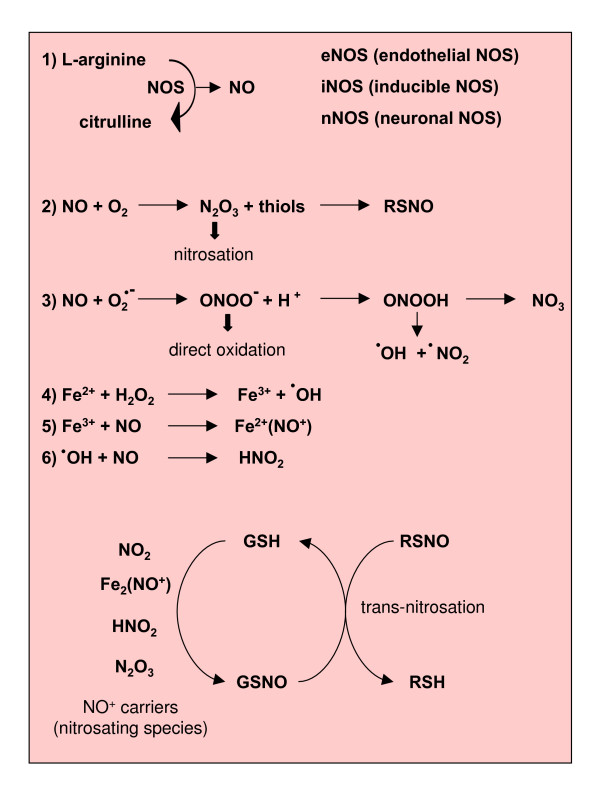
Reactions leading to generation of NO and RNS.

NO is a small hydrophobic molecule that crosses cell membranes without needing channels or receptors [58]. It is generated by NO synthase (NOS) isoforms through the conversion of L-arginine to citrullin. Three types of NOS have been identified: endothelial NO synthase (eNOS), which is bound to plasma membranes and known to be strongly activated by the entry of calcium through membrane-bound receptors [59]; inducible NO synthase (iNOS), which was first identified in macrophages and then in other cells, including hepatocytes, is known to be up-regulated by pro-inflammatory cytokines and/or lipopolysaccharide (LPS), and is able to generate low levels of NO compared with the other NOS isoforms; and neuronal NO synthase (nNOS) (Figure 4).

NO exerts physiological effects by controlling vascular tone, cell adhesion, vascular permeability and platelet adhesion [12,13,15,60]. It also exerts several potentially toxic effects, although many of these are more likely mediated by oxidation products included in the definition 'reactive nitrogen species' (RNS). In particular, NO is able to rapidly react with O_2_^•- ^to form the much more powerful oxidant peroxynitrite (ONOO^-^). Indeed, neither O_2_^•- ^nor NO are particularly toxic *in vivo *because of efficient systems able to minimize their accumulation [61,62]: O_2_^•- ^is removed by SOD isoforms whereas NO is removed as a consequence of its rapid diffusion through tissues [63]. Under pro-inflammatory conditions, simultaneous production of O_2_^•- ^and NO can be strongly activated and significant amounts of ONOO^- ^are generated, which may cause significant injury to different cellular structures.

#### Peroxynitrite

**Figure 5:**
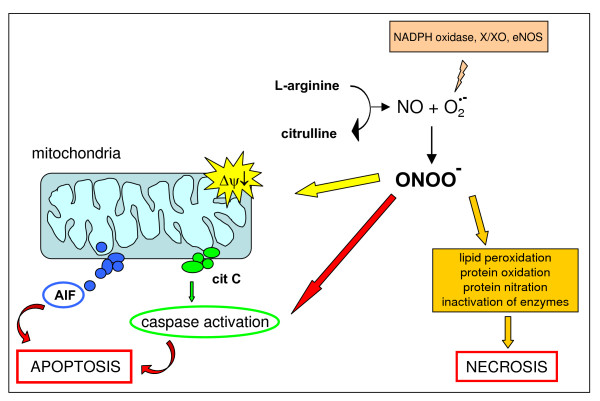
Reactions of peroxynitrite leading to either apoptotic or necrotic cell death. NO and RNS may potentially prevent hepatocyte apoptosis as well as promote either necrotic or apoptotic cell death. The following mechanisms have been proposed. With regard to NO, RNS and prevention of apoptosis, the main molecular mechanisms resulting in an anti-apoptotic effect, related to S-nitrosating species, include [237-239]: stimulation of guanylate cyclase, leading to increased cyclic guanine monophosphate levels; the evolutionarily conserved inhibition of caspases by potentially reversible S-nitrosation of a critical cysteine residue at the caspase active site; activation of the Ras/Erk1/2 pro-survival pathway, which may result in activation of mitogen and stress activated kinase 1 (MSK1) and pp90 ribosomal S6 kinase (RSK), which in turn may inactivate the pro-apoptotic protein Bad or up-regulate anti-apoptotic proteins of the Bcl-2 family [237]; RNS also possibly acting by inhibiting leukocyte adhesion through S-nitrosation of critical -SH groups exposed by activated neutrophils and macrophages [240]. NO and RNS may prevent or promote cell death in relation to intracellular and intramitochondrial (because of mitochondrial NOS) levels of GSH and the concomitant cellular levels of transition metal ions. Moreover, NO may also lead to up-regulation of heme oxygenase 1 (HO-1) in hepatocytes and this may serve as a cytoprotective event [237,238]. The dark (that is, damaging) side of NO and RNS: in the presence of higher levels of ROS, the right NO/superoxide ratio or levels of molecular oxygen, NO may lead again to generation of highly reactive RNS, such as N_2_O_3 _or ONOO^- ^at levels that are able to induce more aggressive oxidation, nitrosation/S-nitrosation and nitration of different biological macromolecules, potentially leading either to necrotic or apoptotic cell death. If NO-dependent pro-apoptotic mechanisms are concerned, the following have been shown to have a major role, with some again depending on S-nitrosating species: RNS and so called NO^+ ^-carriers (nitrosating species) may result in activation of JNK, which, as previously reported for ROS, may sustain induction of apoptosis; NO, if generated at high levels in mitochondria, may result in ubiquinol auto-oxidation with concomitant production of superoxide, hydrogen peroxide and ONOO^-^, species that may be responsible for irreversible damage to complexes I and II of the respiratory chain, inhibition of ATP synthesis and eventually cytochrome *c *release and induction of caspase-dependent apoptosis. It should also be noted that, in the presence of significant redox stress, NO can potentiate damaging effects, resulting in a scenario of necrotic cell death rather than apoptosis. This is likely to occur particularly when the redox state is significantly affected, as in conditions resulting in depletion of GSH or significant alterations of the GSH/GSSG ratio.

ONOO^- ^(see [58] and references therein) is a strong oxidant able to react directly with thiol groups, iron-sulphur centres and the active site -SH groups in tyrosine phosphatases. In physiological conditions, the production of ONOO^- ^is quite low and oxidative injury is minimized by endogenous antioxidant defences. When increased in pathological conditions, ONOO^- ^can act either as a direct oxidising species or indirectly by decomposing into highly reactive radicals. When ONOO^- ^acts as an oxidant, it produces nitrite and a hydroxide ion rather than isomerising to nitrate (Figure 4) and can react with proteins (tyrosine nitration or direct reactions with specific amino acids), lipids (lipid peroxidation) and nucleic acids (oxidative modifications in nucleobases). ONOO^- ^can also interact with mitochondria, reaching them from extra-mithocondrial compartments or being locally produced through the interaction of NO (generated by the mitochondrial NOS) and O_2_^•-^. Mitochondrial toxicity of ONOO^- ^results from direct oxidative reactions of principal components of the respiratory chain or from free radical-mediated damage. Persistent generation of significant levels of ONOO^- ^can lead to the induction of cell death, either apoptosis or necrosis (Figure 5; see also 'Mitochondria, nitric oxide, RNS and cell death' below).

### How ROS and other oxidative stress-related reactive intermediates interact with biological macromolecules

ROS, NO, HAKS and other free-radical or non-radical reactive intermediates may interact with relevant biological macromolecules [7-9,14,15,19,21,58], events that can easily lead to cytotoxic/damaging consequences or contribute to redox regulation and signalling.

#### ROS and other pro-oxidants

ROS and other pro-oxidants can interact with virtually any macromolecule of biological interest. Their interaction with DNA can lead to oxidative damage, strand breaks and the formation of adducts (such as 8-hydroxy-deoxyguanidine (8-OH-dG)). By interacting with polyunsaturated fatty acids in membrane phospholipids, ROS and reactive pro-oxidants can elicit peroxidation of lipids and their subsequent degradation and fragmentation. When interacting with proteins, ROS may lead to: (a) oxidation of critical amino acid residues, for example, the thiol group of cysteine; (b) formation of intra-molecular disulfide bonds (-S-S-); (c) thiol/disulfide changes leading to either formation or disruption of inter-molecular disulfide bonds between homo- or hetero-dimers; (d) formation of di-tyrosine and protein cross-linking; and (e) iron and copper metal ions can lead to the formation of OH radicals in a Fenton reaction – this can extensively damage target proteins, leading to their ubiquitination and proteasomal degradation. Reactions (a-c) can either lead to functional inactivation of the target protein or (c) convert a protein between its active and inactive states. Reactions (d) and (e) can lead to the formation of new antigens that the immune system may recognize as non-self.

#### Nitric oxide and reactive nitrogen species

RNS such as ONOO^- ^[58] can easily lead to oxidation and formation of strand breaks when reacting with nucleic acids or to lipid peroxidation when interacting with membrane lipids. Again, RNS may simply lead to oxidative modification of proteins or to more selective reactions by nitrosation or nitration (Figure 5).

#### 4-Hydroxy-2,3-nonenal

HNE, an aldehydic end product of lipid peroxidation, can exert both cytotoxicity as well as signalling modulation by forming Michael type adducts on lysine, cysteine or histidine residues [19-23]. HNE and other HAKs can also interact with nucleic acids, leading to formation of DNA adducts or even to strand breaks and genotoxicity. HNE may also operate by eliciting intracellular generation of ROS when interacting with mitochondria [23].

## Antioxidant defences

**Figure 6:**
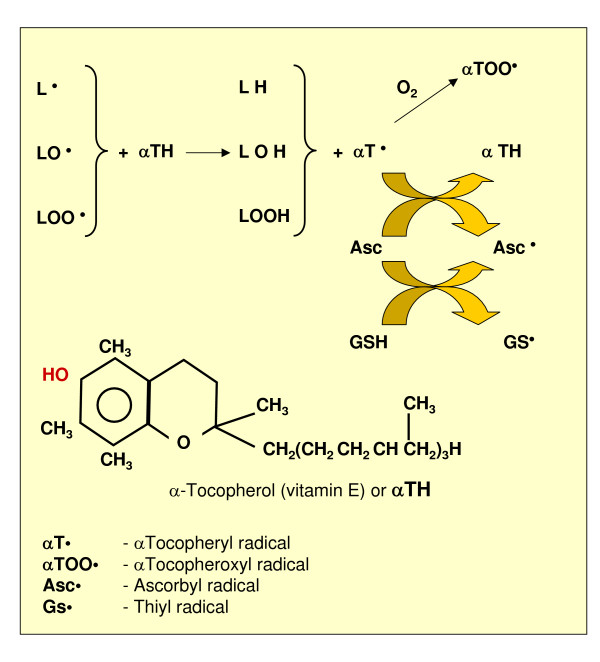
Antioxidant (chain breaking) action of α-tocopherol and its recycling through ascorbate and GSH.

**Figure 7:**
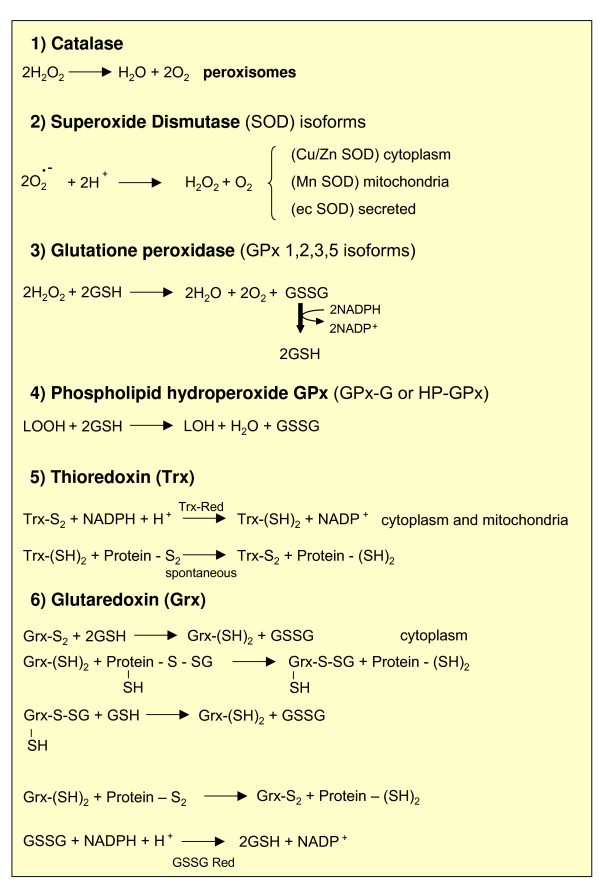
Overview of antioxidant enzymes.

**Figure 8:**
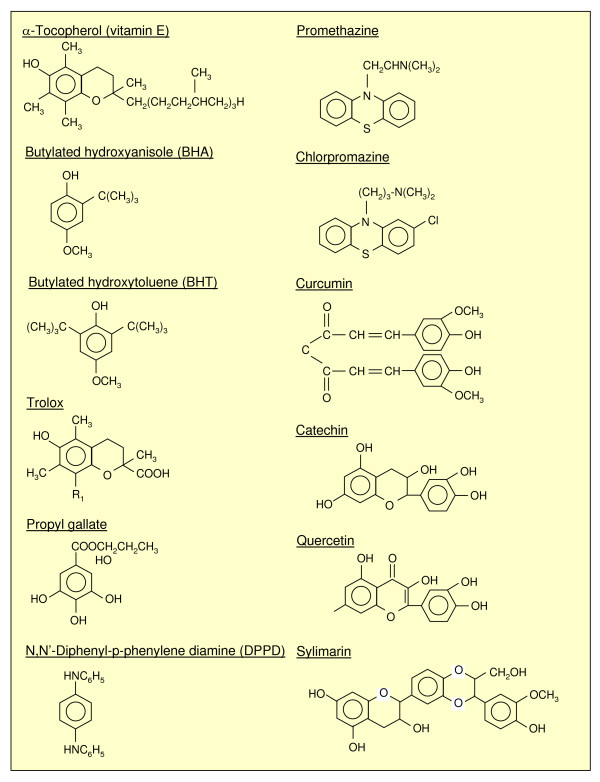
Chemical structures of the most common chain-breaking antioxidants.

Antioxidant defences rely on the sum of those mechanisms that nature has developed to protect biological tissues from ROS and other oxidants and from lipid peroxidation (Figures 6, 7, 8. With respect to the 'hepatic' focus of this review, the reader should note that all clinical and experimental conditions of CLDs (that is, those leading to fibrosis/cirrhosis) have in common a sharp and significant decrease in antioxidant defences (reviewed in [35,36]). More details on antioxidant defences can be found in [42,64-66].

### Protection from ROS and oxidants

The following categories of naturally occurring components may be defined.

#### Antioxidant enzymes

This group of antioxidant enzymes includes 'major' enzymes such as catalase, glutathione peroxidase (GPX) isoforms and SOD isoforms. Catalase and GPX isoforms are responsible for the removal of H_2_O_2 _as well as other organic hydroperoxides, whereas SOD isoforms operate by transforming O_2_^•- ^into H_2_O_2 _(Figure 7).

#### Protection by small molecules

Small molecules involved in protection from ROS and oxidants include ascorbic acid, reduced glutathione (GSH) and uric acid. Ascorbic acid (vitamin C) is a cofactor for several enzymes that has the ability to act as an electron donor and then as a reducing agent; ascorbate can also scavenge (that is, interact directly with) •OH but one has to briefly mention that, depending on the overall concentration, ascorbate may become deleterious by reducing Fe^3+ ^to Fe^2+ ^and, in the presence of H_2_O_2_, lead to the generation of significant amounts of •OH. GSH is a hydro soluble tripeptide acting as a substrate for H_2_O_2_-removing enzymes such as GPX and dehydroascorbate-reductase as well as a scavenger of •OH (leading to the thiyl radical GS•, which is not harmless) or as a thiol in regenerating the oxidized -SH groups of proteins; Figure 6 also shows the essential reaction of GSSG reductase, which recovers GSH. Uric acid, present in blood plasma, has been reported to scavenge singlet oxygen, •OH and peroxyl radicals.

#### Protection by sequestration of metal ions

Transition metal ions like iron and copper can exacerbate ROS generation. Ferritin, transferrin, ceruloplasmin, metallothionein and lactoferrin can thus be seen not only as relevant for their respective role in metal homeostasis but also as molecules that, by 'sequestering' redox active metal ions, may prevent ROS production via the Fenton reaction.

#### Thioredoxin and glutaredoxin systems

Thioredoxins (Trxs, including Trx-1 and Trx-2) [65,66] are 12 kDa proteins with a catalytic site containing two cysteine residues that can be oxidized reversibly to form disulfide bridges. Trxs undergo NADPH-dependent reduction by Trx-reductase and, in turn, they can reduce oxidized cysteine groups on proteins. Through this intramolecular disulfide-thiol exchange, Trxs can act as hydrogen donors, contributing to the control of redox state. Trxs (mainly Trx-1) may supply reducing equivalents to a number of Trx peroxidases (peroxiredoxins) and also play a role in redox signalling by modulating kinases or transcription factors by forming heterodimers with them.

Glutaredoxins (Glrxs, the cytosolic Glrx-1 isoform and Glrx-2, the latter existing as both mitochondrial and nuclear isoforms) [66] also belong to the Trx superfamily of thiol/disulfide exchange proteins and act as reductants of protein-SG mixed disulfides. Similar to what was described for the Trx system, Glrxs have a role in redox regulation and the Glrx system is composed of Glrx isoforms, GSH reductase, GSH and NADPH.

### Protection from lipid peroxidation: natural and synthetic antioxidants

According to Halliwell and Gutteridge [64] "an antioxidant is any substance that, when present at low concentrations compared to those of an oxidizable substrate, is able to significantly delay or inhibit oxidation of that substrate." Of course, this generic definition also includes primary antioxidants (free radical scavengers able to interact directly with and/or to block the initiating free-radical, such as mannitol) and synthetic molecules able to bind metal ions (for example, desferrioxamine). However, several authors, when using the word 'antioxidant', have in mind the so-called 'chain breaking' or 'secondary antioxidants', with α-tocopherol (vitamin E) being the naturally occurring prototype. These natural or synthetic molecules have a chemical structure (Figure 8) able to intercept radical intermediates produced during on-going lipid peroxidation, such as peroxyl or alkoxyl radicals, thus preventing (that is, 'breaking') the perpetuation of hydrogen abstraction in the chain reaction. Figure 6 offers an overview of the reactions involving α-tocopherol, including its re-cycling based on the involvement of GSH and ascorbate.

A number of additional and useful concepts regarding antioxidants (whether enzymic or not) should be considered: antioxidants binding metal ions are not usually consumed during the course of reaction; antioxidants able to decompose peroxides may be consumed or not, depending on their nature (for example, as enzymes GPXs are not consumed); chain breaking – as well as primary – antioxidants are usually consumed during on-going oxidative stress; many antioxidants have multiple mechanisms of action; some antioxidants (tocopherols, ubiquinol, carotenoids and flavonoids) will exert their effects in a lipid phase (that is, at the level of biological membranes) whereas others will do so in an aqueous phase (ascorbate, urate, GSH and other thiols).

## Redox homeostasis, redox signalling, redox sensors and redox-dependent transcriptional regulation in mammalian cells: the good, the bad and the ugly

Redox signalling is a definition that can be used to indicate any physiological or pathophysiological condition in which a process can be regulated or modulated by a signal that is delivered through redox chemistry [14-18]. When significant levels of ROS are generated in a biological system (that is, altering redox homeostasis), 'redox signalling' then represents the response or part of the response designed to 'reset' the original state of equilibrium. As in any complex system reacting to the presence of defined reactants, single cells and multicellular organisms have developed highly specific redox sensors and mechanisms that form the basis of oxidant scavenging and ROS signalling systems.

### Principles of redox homeostasis

**Figure 9:**
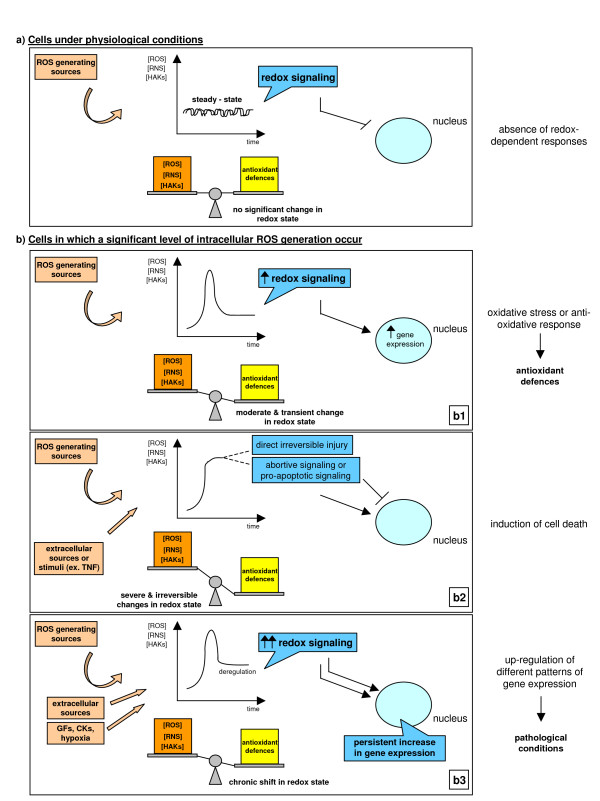
Alteration of redox homeostasis, redox signaling and cellular responses. Figure 9a: cells under physiological conditions in the absence of redox-dependent responses. Figure 9b: cells in which a significant level of intracellular ROS generation occurs as a moderate and transient change in redox state (b1), as a severe and irreversible change leading to cell death (b2) or as a chronic shift in redox state (b3).

To introduce the concept of redox homeostasis one can refer to the scenario depicted in Figure 9 and to the intuitive concept of oxidant/antioxidant balance, which is still the simplest way to begin understanding the complexity of redox mechanisms.

#### Physiological conditions or unstimulated cells

Under physiological conditions, relatively low amounts (steady-state levels) of ROS, free radicals and other reactive intermediates are produced as a result of a dynamic balance between the rate of their generation and removal. Redox homeostasis is primarily controlled by catalase, Trxs, SODs and GPXs, as well as by naturally occurring antioxidants like GSH, vitamin E, β-carotene, ascorbate, urate, and many others. However, enzymes and natural antioxidants that are highly specific are present at relatively low concentrations; the antioxidant arm of the so-called oxidant/antioxidant balance is significantly implemented by less specific or efficient, but much more abundant, actors that are represented by amino acids, peptides and proteins [14-18,42,65,66]. Indeed, almost all amino acids may serve as molecular targets for pro-oxidants (cysteine, tyrosine, tryptophan and histidine being the most sensitive to oxidants) and the intracellular concentrations of free amino acids reach levels as high as 10^-1 ^M. Oxidized proteins may offer an additional contribution: oxidative attack on proteins can lead to inactivation, fragmentation, aggregation of fragments and/or increased susceptibility to proteolysis. Proteolytic degradation of oxidized proteins is mainly due to 20S proteasome and it has been proposed that oxidatively fragmented and/or misfolded proteins have an increased ROS scavenging activity than normal, non-oxidized proteins [67-69]. In practical terms, cells in which very low levels of ROS are generated (a baseline-like or steady state condition, as in Figure 9a) do not suffer a significant imbalance in pro-oxidants versus antioxidant defences and do not respond by means of a redox signalling.

#### Cells exposed to an 'acute' and/or 'isolated' versus chronic generation of ROS

Whenever redox homeostasis is significantly disturbed – by an increase in ROS generation, by a decrease in one or more antioxidants or by a change in the thiol/disulfide redox state – redox signalling can be elicited [14-18]. Three different scenarios, depending on the absolute intracellular levels of ROS and other reactive species or the temporal length of the alteration, may be envisaged (Figure 9b).

In the first scenario, the increase in ROS is relatively low and transient (Figure 9b1). In these conditions, the shift in redox balance will be limited and redox signalling will operate through redox-sensitive signalling pathways and transcription factors [14-18] in order to up-regulate genes encoding products that will reset redox homeostasis (enzymes, Trxs and Glrxs, the cystine transport system to sustain the production of GSH, and so on).

In the other two scenarios, oxidative stress is more severe (Figure 9b2 and 9b3). During acute tissue injury or in tissues undergoing chronic injury, levels of intracellular ROS and other reactive intermediates may be very high and/or persistently increased within cells. The 'oxidative stress response' may not be sufficient to contain disturbances and reset the original redox homeostasis. Depending on several factors (that is, the specific agent or condition involved, the overall severity of the injurious process, and so on) this may cause at least two scenarios. Levels of ROS or reactive intermediates may be high enough within the cells to significantly damage macromolecules or alter cellular structures and functions, eventually leading to irreversible injury and cell death (Figure 9b2). If levels of oxidative stress are significantly higher but are not able to induce irreversible cell damage, as may occur in conditions of chronic injury, cells and/or tissues may still reach an equilibrium or, as elegantly defined by Dröge [15], a 'quasi-stable state' (Figure 9b3). This definition implies a shift of the intracellular redox state to higher levels of ROS and a chronically deregulated state in which redox signalling can up-regulate patterns of gene expression and cell responses that are believed to significantly contribute and/or sustain the development of chronic diseases and even cancer progression [15,70]. Of course, the scenario given in Figure 9b1–b3 is a didactic one and in a tissue undergoing chronic injury, inflammation and wound healing the three conditions are likely to coexist, with an overall scenario in which the development of the disease results from the sum of both ROS-dependent damaging effects and changes in gene expression.

### Redox sensors and the basis of redox-dependent transcriptional regulation

At this point one should move beyond the simple concept of oxidant/antioxidant balance by introducing the more refined notion of redox sensors as well as the principles of redox-dependent regulation of transcription. The key messages in this area (for more details see [18,71]) can be summarized as follows.

#### The definition of 'redox sensors'

A redox sensor is a specialized redox-sensitive protein that is able to 'sense' or 'measure' intracellular levels of ROS by a redox-based mechanism affecting one or more residues/domains within its three-dimensional structure, and to transform the redox change into a specific setting for antioxidant activity-related transcription and, particularly for mammalian cells, much more.

#### Redox sensors in prokaryotic cells and yeast

**Figure 10:**
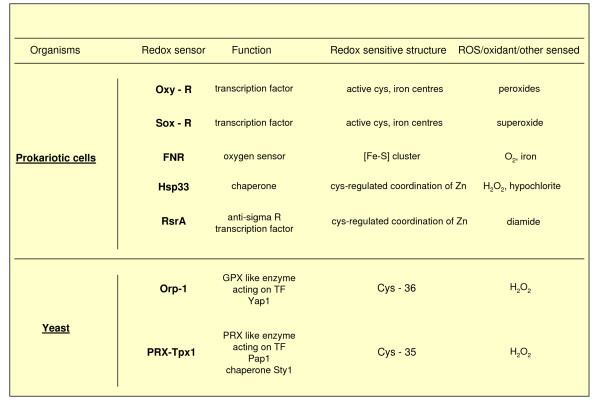
Relevant examples of redox sensors in prokariotic cells and yeast. In the case of redox sensors described in yeast, the following can apply. The redox sensor Orp-1 of *Saccharomyces cerevisiae *(Oxidant receptor peroxidase-1, also known as Gpx3) is known to interact with hydrogen peroxide at Cys36, forming a -SOH group that, in turn, will lead to rearrangement (disulphide bonds) in the OxyR analogue Yap1 transcription factor and in the associated Ybp1 protein, leading ultimately to Yap1 nuclear translocation and Yap1-dependent gene activation [18,71]. Similar systems have also been described in *Schizosaccharomyces pombe *and a very similar mechanism has been described for PRX-Tpx1.

Redox sensors were first described in bacteria, including the OxyR and SoxR redox sensitive transcription factors, the chaperon molecule Hsp33, the oxygen sensor FNR and others. All these 'redox receptors' have a structure designed to sense specific ROS, oxidants or other reactive intermediates. These ancestral redox sensors can essentially contribute to fast mechanisms designed to deal with ROS and to make adjustments allowing the survival of the bacteria (that is, to reset redox homeostasis). During evolutionary development these simple bacterial sensors have been replaced with more specifically designed proteins, such as yeast thiol peroxidases (enzymes belonging to the family of peroxyredoxins or GPXs), which contribute to H_2_O_2 _signalling (see Figure 10 for more details).

#### Redox sensors in higher eukaryotes

**Figure 11:**
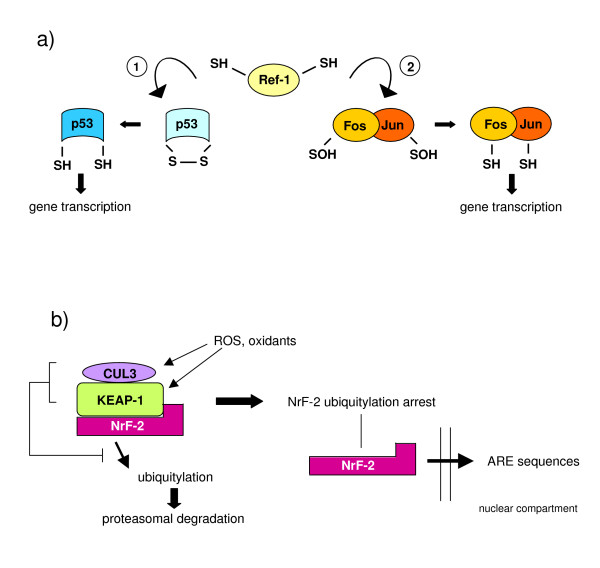
Redox sensors, redox signalling and control of redox sensitive transcription in higher eukaryotes. **(a) **Redox reactions involving transcription factors such as Ref1 (Redox-factor-1); Ref-1 is a ubiquitous reductase having cysteine residues (Cys65 and Cys94) that are believed to be critical for redox-dependent modification of several transcription factors, including AP-1 (activator protein-1), NF-κB (nuclear factor κB), p53, ATF/CREB (activating transcription factor/cAMP-response element-binding protein), and HIF-1α (hypoxia-inducible factor 1α). Ref-1 acts by reducing -SOH groups and/or oxidized cysteine residues or disulphide bonds present on transcription factors that, under these 'oxidized' conditions, have reduced or absent DNA-binding activity; the 'reduced' transcription factors then become able to bind their related sequences on DNA (shown here are the two examples Ref-1/p53 1 and Ref1/AP-1 2). **(b) **Nuclear translocation of transcriptional regulators that are maintained in an inactive form in another cellular compartment; a characteristic example is Nrf-2 (nuclear factor (erythroid-derived-2)-like-2), which is a transcriptional regulator able to bind to the so-called ARE (antioxidant responsive elements) regulatory sequences that are located on genes encoding a number of enzymes involved in detoxification, including those for glutathione S-transferases, NAD(P)H quinine oxidoreductase, the multidrug resistance-associated protein and cysteine-glutamate exchange transporter, thus up-regulating their transcription. In this case, Nrf-2 is usually bound to KEAP-1 (Kelch-like ECH associated protein-1) receptor or sensor, a protein rich in cysteine residues that usually forms a complex with cullin-3 and Nrf-2 to target the latter for proteasomal degradation. Exposure to oxidative stress (oxidation of Cys151, Cys273 and Cys288 combined with other reactions, including a Cys-zinc redox centre) results in modification of KEAP-1, leading to arrest of Nrf-2 ubiquitylation, allowing Nrf-2 to detach from KEAP-1 and translocate into the nucleus. **(c) **Modulation of transcription by alterations in the so-called 'redox buffer'; this concept indicates simply that several transcription factors as well as DNA modifying enzymes are sensitive to the most relevant reduced/oxidized molecular redox pairs, such as GSH/GSSG, NADPH/NADP and NADH/NAD. Examples of this way of coupling redox status to transcription factors or chromatin modifying enzymes include proteins that regulate circadian rhythms (Clock, NPAS2 and BMAL1), the protein for transcriptional silencing related to lifespan, SIRT1, and the transcriptional repressor C-terminal-binding protein (CtBP).

In higher eukaryotes redox regulation of transcription, as well as of signalling elements like protein phosphatases, relies on properties and strategies similar to those described for bacteria or yeast (cysteine-based oxidation/reduction cycles), which have been evolutionarily conserved. Here we still see thiol peroxidases affecting H_2_O_2_-dependent signalling, with some crucial differences since PRXs and GPXs have been reported to be involved in the modulation of signal pathways. Figure 11 illustrates established examples of three different mechanisms by which an increase in intracellular ROS may trigger transcription of redox sensitive genes: redox reactions directly involving either signalling components or transcription factors; nuclear translocation of transcriptional regulators that are maintained in an inactive form in another cellular compartment; and modulation of transcription by alterations in the so-called 'redox buffers'. More details can be found in the legend to Figure 11 and in [18,71-73].

#### The meaning of redox sensors and redox signalling in higher eukaryotes

In mammalian cells, ROS-specific responses such as those regulated by p53, activator protein (AP)-1, nuclear factor (NF)-κB, c-Myc, FOXO and other factors can be seen as part of long-term differentiation programmes that integrate ROS protection and multiple metabolic/adaptative responses. Higher eukaryotes have developed strategies that, by diverting the original defensive design of redox signalling, use intracellular ROS produced within cells to modulate several signalling pathways, such as those downstream of growth factor receptors. Redox changes and ROS signals may potentially simultaneously affect different signalling pathways, modulating main metabolic or adaptative responses of cells and playing a strategic role in several physiological or pathophysiological conditions, including many chronic diseases of clinical relevance.

## Chronic injury and liver fibrogenesis: the tissue, cellular and molecular scenario involving liver parenchyma as a paradigm to introduce the role of ROS and redox signalling

**Figure 12:**
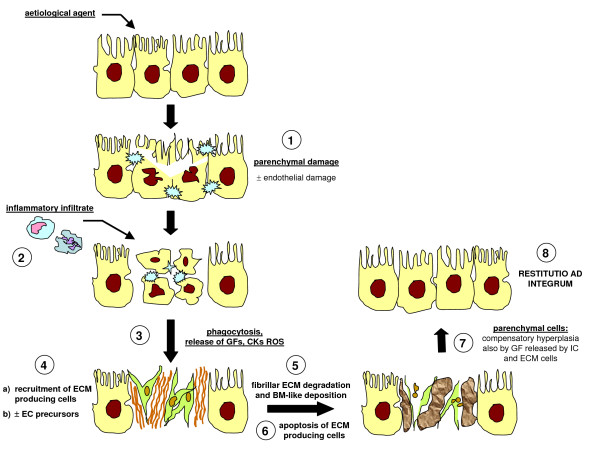
Acute liver injury: when healing is a coordinated and sequential process. A standard acute liver injury leading to irreversible parenchymal damage is followed by recruitment in the injured site of resident (Kupffer cells) or peripheral blood-derived activated monocyte/macrophages, resulting in phagocytosis, and the release of growth factors, cytokines, chemokines and ROS. Healing proceeds with recruitment of ECM-producing cells (HSCs and/or portal fibroblasts) and, likely, also of endothelial progenitor cells (EPCs): recruitment of these cells is essential to provide deposition of new ECM (basal membrane-like) and to form new sinusoids. Next, HSCs in excess will undergo apoptosis and the '*restitutio ad integrum*' will require compensatory hyperplasia of hepatocytes that have survived the original injury as a response to a number of growth factors released by either inflammatory cells, endothelial cells or HSCs.

Liver tissue has a unique ability to respond to different injuries leading to parenchymal damage, which may also include damage to endothelial cells and sinusoids as well as to other non-parenchymal cells. Following a single acute injury, healing in the liver can be envisaged as a highly coordinated and sequential process (the more relevant steps are summarized in Figure 12) involving recruitment of inflammatory cells and extracellular matrix (ECM)-producing cells and compensatory hyperplasia of hepatocytes, with the final goal of '*restitutio ad integrum*'. The response to acute liver injury may vary, as in fulminant acute liver failure and/or in the presence of specific toxins or carcinogens, by involving a response also including proliferation, plus differentiation, of bi-potent hepatic progenitor cells (HPCs) located at the level of the ductules of Hering (see [74] and references therein).

**Figure 13:**
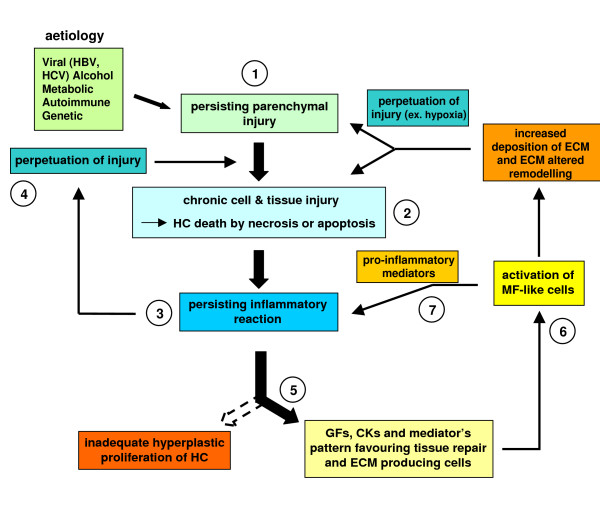
Schematic representation of events involved in fibrosclerotic development of CLDs. CLDs may involve different aetiological agents or conditions able to cause persisting parenchymal liver injury (1) and then hepatocyte (HC) cell death (either necrosis or apoptosis) (2). As a result, a persistent inflammatory reaction can occur (3), which may significantly affect the progression of the disease by either contributing to the perpetuation of injury (4) or 'creating' a growth factor, cytokine and mediator pattern favouring tissue repair and the activation of ECM-producing cells (5). This chronic scenario will lead to activation of myofibroblast-like cells that will contribute either to perpetuation of inflammation by releasing pro-inflammatory mediators (7) or to the wound healing response by excess and progressive accumulation of fibrillar (rich in collagen type I and III) extracellular matrix (ECM) components. If the aetiological agent or causal condition persists, the CLD can undergo a fibrosclerotic progression to cirrhosis and liver failure [74-81]. Cirrhosis in turn may be defined as an advanced end-stage of fibrosis, characterized by formation of regenerative nodules of parenchyma surrounded and separated by fibrotic septa, a scenario that is intrinsically associated with significant changes in hepatic angio-architecture [81-83].

**Figure 14:**
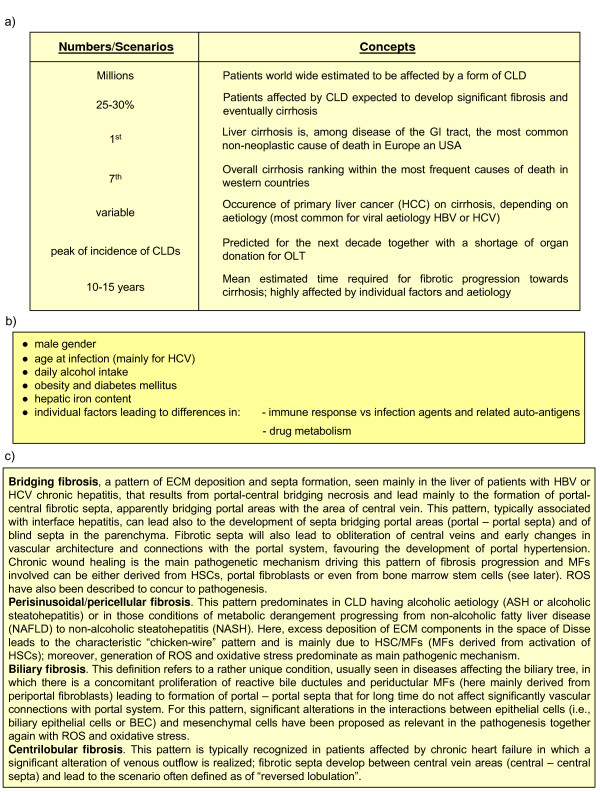
Concepts and numbers. **(a) **The clinical impact of progressing fibrogenesis. **(b) **Predictors for fibrosis progression in CLDs. **(c) **Patterns of fibrosis progression in CLDs.

The scenario changes significantly in CLDs, which are typically characterized by persisting liver injury due to chronic infection by hepatotropic viruses (mainly HCV and HBV) as well as to autoimmune, metabolic, toxic or drug-induced causes, with ethanol consumption representing either a major single cause of toxic chronic injury or a very common additive one. As a result of these conditions (more details are given in Figure 13), persistent inflammatory reaction and chronic activation of the wound healing response will occur, sustaining progression of fibrogenesis to the end-point of cirrhosis [74-83]. The dynamic motor of CLD progression is likely to be represented by fibrogenesis. Figure 14 briefly summarizes the impressive 'numbers' that reveal the global clinical impact of progressive fibrogenesis and indicates those features that are likely to serve as major predictors of fibrosis progression in a CLD. An extensive review of liver fibrogenesis and its progression to cirrhosis is beyond the scope of this review and the interested reader can refer to several reviews in the specific field [75-84]. Here only crucial tissue, cellular and molecular concepts and mechanisms will be mentioned.

### Patterns of fibrosis progression in CLDs

Fibrosclerotic progression follows distinct patterns that are intrinsically related to the aetiological cause of the CLD and the topographic site of tissue injury, as well as to the predominant pro-fibrogenic mechanism and the involvement of populations of pro-fibrogenic myofibroblast-like cells of different origin (MFs). Four main patterns of fibrosis have been identified and are described in detail in Figure 14.

### Myofibroblast-like cells as pro-fibrogenic effectors in CLDs

**Figure 15:**
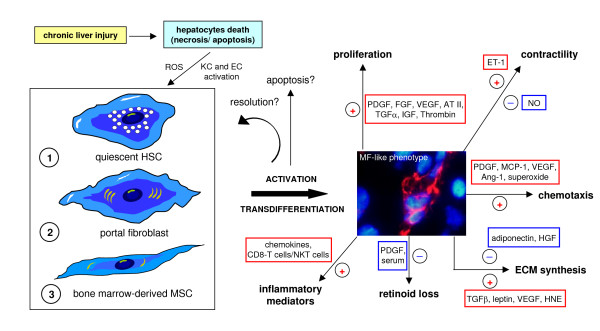
Origin of MF-like cells and their activation in the scenario of a CLD. Myofibroblast-like cells (MFs) may originate, under CLD conditions, from either quiescent HSCs, portal fibroblasts or bone marrow-derived MSCs able to engraft the chronically injured liver. Whatever the origin, MFs are believed to be characterised by the following properties and phenotypic responses: **(a) **high proliferative attitude; **(b) **increased ability to synthesise ECM components, particularly collagen type I and III; **(c) **altered ability to express matrix metallo-proteinases (MMPs) and related tissue inhibitors (TIMPs), resulting in an altered ability to remodel ECM in excess; **(d) **increased ability to migrate in response to different stimuli, including truly chemotactic ones; **(e) **increased synthesis of growth factors and pro-inflammatory cytokines and chemokines [76], including pro-angiogenic cytokines [81,83,92,93], that may act as paracrine as well as autocrine mediators; **(f) **contractility in response to vasoactive compounds like NO, endothelins and others; **(g) **the potential to undergo apoptosis in case of removal of the aetiological agent (that is, successful therapy, alcohol withdrawal, and so on) or causative conditions [80,81,91], although fibrosis regression has been mainly observed in experimental models. Here it should be mentioned that although there is no doubt that fibrosis is, at least in principle, a potentially reversible process, a complete reversion of cirrhosis (particularly for human cirrhotic livers) has never been convincingly documented [84] and human HSC/MFs have been shown to possess a peculiar survival attitude both *in vitro *as well as *in vivo *that may indeed favour progression over reversion [94].

MFs are pro-fibrogenic cells found in chronically injured liver in either experimental or clinical conditions. They are characterized by a positive stain for α-smooth muscle actin (α-SMA). The origin of liver MFs has been a matter of controversy for more than a decade but now there is substantial agreement on the following major concepts (summarized in Figure 15). First, three different phenotypes of MFs have been identified in fibrotic/cirrhotic livers of human patients or in animal models, including hepatic stellate cells (HSCs) that become activated or HSC/MFs (in capillarised sinusoids), interface myofibroblasts (MFs located at the interface between fibrotic septa and the surrounding parenchyma) and portal/septal myofibroblasts (MFs in the expanded portal areas or within fibrotic septa) [85,86]. Second, liver MFs have multiple origins, with most originating from HSCs; indeed, most of our present knowledge comes from studies investigating this peculiar liver cell population, as recently reviewed by Friedman [81]. HSCs are likely to give rise to HSC/MFs and to most interface MFs; MFs can also originate from portal fibroblasts, which are also able to give rise to the phenotypically identical septal MFs. A significant number of MFs can also originate, in chronically injured human [87] and murine livers [88,89], from bone marrow-derived mesenchymal stem cells (MSCs), suggesting caution when considering therapeutic procedures involving autologous transplant of bone marrow-derived stem cells [90]. Third, whatever the origin, all the mentioned 'precursors' of ECM-producing cells in CLDs are likely to undergo a similar process of activation and trans-differentiation that leads to the peculiar MF-like phenotype [82,90]; thus, the actual 'feeling' is that HSC/MFs, and likely all activated MF-like cells, may share, from a functional point of view, the ability to exhibit a number of phenotypic responses [80,81,91] (summarized in Figure 15), including proliferation [76,81,83,92,93], synthesis and remodelling of ECM and of mediators, migration, contractility and the potential to undergo apoptosis [80,81,91,94]. More details are given in the legend of Figure 15.

### Major events, cells and mechanisms regulating liver fibrogenesis in CLDs

Several relevant events for fibrosclerotic progression of CLDs may be considered relatively independent of the specific aetiology, as detailed in Figure 13; in these conditions of persisting tissue injury, ROS and other related mediators, released by damaged cells or activated inflammatory cells, are likely to play a relevant role. Along these lines, it should be noted that, in chronic diseases, both necrotic and apoptotic, as well as apoptosis-like, forms of cell death have been reported to occur and have been detected in the same tissue section [80,95,96] and that hepatocyte apoptosis represents an effective pro-fibrogenic stimulus [97,98].

**Figure 16:**
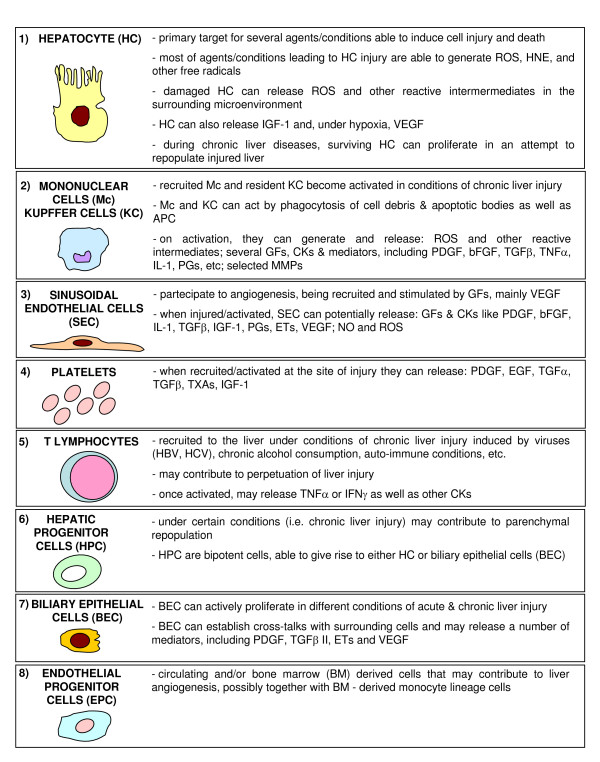
The other cellular 'actors' in hepatic chronic wound healing: the roles of the different cell types, including those that may be related to redox state and signaling.

To complete the oversimplified scenario offered in Figure 13 (more details in [74,91,95]), the following concepts should be recalled. First, in such a complex scenario several other cellular 'actors' are likely to be involved; Figure 16 summarizes all the cells involved in CLDs, indicating how they can contribute to fibrogenesis progression and, for some of them, to the generation of ROS or related reactive intermediates. Second, a major pathogenic role has to be attributed to several soluble factors (produced by different kind of cells; Figure 16) that can regulate both the state of activation of MFs and their phenotypic responses as well as the responses of other cells involved; these factors include platelet-derived growth factor (PDGF), transforming growth factor (TGF)β, connective tissue growth factor (CTGF), endothelin-1 (ET-1), monocyte chemotactic protein (MCP)-1, and tumour necrosis factor (TNF), to name just a few. Third, several signalling pathways, transcription factors and related transcriptional gene regulation have been dissected and identified as involved in the process of activation of HSCs or in mediating phenotypic responses of MFs, and most of these (see next section) are known to be redox-sensitive. Fourth, angiogenesis, pro-angiogenic cytokines and expression of related receptors are emerging as crucial factors potentially able to contribute actively to liver fibrogenesis [83,92,93]. Finally, oxidative stress as well as increased generation of ROS, HNE, NO and RNS have been unequivocally detected in all clinical conditions and animal models of fibrogenic CLD; moreover, administration of antioxidant agents in animal models usually offers prevention ([35,36] and references therein).

## ROS and intracellular signalling cascades: redox sensitive molecular targets in signalling pathways likely to be involved in chronic wound healing

Signal transduction elicited by interaction of peptide factors (growth factors, cytokines, chemokines as well as other ligands) with their respective receptors can be enhanced or modulated by intracellular ROS generation. Indeed, peptide ligands also trigger activation of NOX but the positive feedback on signal transduction can also be elicited whatever the source of ROS, including ROS produced by mitochondria and other intracellular sources or entering the cell from the extracellular environment. The latter can include H_2_O_2_, which has a rather long half-life, a relatively low reactivity and an intrinsic ability to cross biological membranes. The literature concerning redox sensitive signalling pathways is now impressive (for more details, see [14,15,17,49]) and here we present the most established concepts that may have a major role in chronic wound healing.

### ROS and receptor-mediated signalling pathways

**Figure 17:**
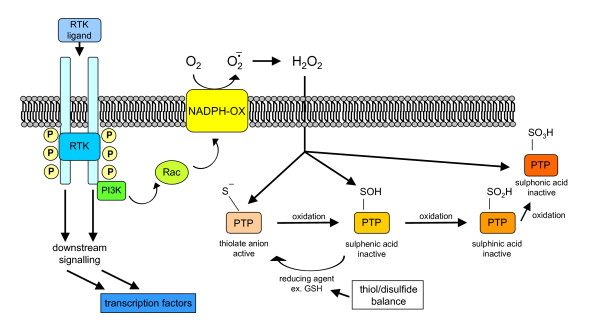
ROS may modulate receptor tyrosine kinase (RTK) signalling by regulating protein tyrosine phosphatases (PTPs) redox state. When a peptide ligand such as PDGF binds to its receptor RTK on the surface of a non-phagocytic cell (for example HSC/MFs), the signal can involve activation of PI3K and Rac, which in turn will result in activation of membrane NOX and generation of ROS. Within the cell ROS, such as H_2_O_2_, may act on a redox-sensitive cysteine residue in the active site of PTPs and transform the -SH group into the oxidized – SOH group (sulphenic acid), thus reversibly inactivating PTPs. Under physiological conditions and with low levels of ROS this change is rapidly reverted by reducing agents, with this transient redox inhibition of PTPs having a relevant role in RTK signalling. However, in conditions in which intracellular ROS are significantly increased, this may lead to more oxidation and then to irreversible changes, with formation at the level of the sensitive cysteine residues of sulphinic and sulphonic acid. These oxidized forms of PTPs are inactive and this will result in long-lasting blocking of PTP-dependent receptor dephosphorylation, allowing a positive reinforcement of RTK downstream signal transduction. The intracellular thiol/disulfide balance potentially plays a relevant role here: cellular levels of GSH or other reducing agents, for example, may operate to revert the sulphenic acid group in the active site of PTP to the thiolate anion, converting PTP back to the active state.

ROS have been shown to mediate a positive feedback on signal transduction elicited by, for example, PDGF, epidermal growth factor or nerve growth factor; this usually reinforces the receptor tyrosine kinases (RTKs) (Figure 17) and involves p21Ras and Rac, leading to activation of a subunit of non-phagocytic NOX, likely a gp91^phox ^analogue. TGFβ1, which operates by binding receptor serine/threonine kinase and involves Smads and Src kinases, as well as other relevant ligands in a scenario of chronic wound healing (as in CLDs), including interleukin (IL)-1, TNF, angiotensin II (Ang II), thrombin and insulin, have also been described to lead to activation of a NOX in non-phagocytic cells to generate O_2_^•-^, which will then spontaneously or enzymatically dismutate into H_2_O_2_. The reader may then envisage a scenario in which non-phagocytic cells involved in chronic wound healing receive signals from the extracellular milieu, such as those derived from peptide factors binding their receptors or extracellularly generated ROS or other oxidants, and then face an increase in intracellular ROS that affects signalling pathways by one or, more likely, two or more of the following mechanisms.

#### ROS can enhance signalling pathways by inhibiting protein tyrosine phosphatases

Protein tyrosine phosphatases (PTPs) can be considered as negative regulators of RTK-mediated signalling, switching off the activated receptor by means of dephosphorylation [49,99-101]. However, ligand-induced activation of RTKs can lead (as for PDGF signalling; Figure 17) to phosphoinositide 3-kinase (PI3K)-mediated activation of Rac that, in turn, is able to switch on ROS generation by NOX [49,99]. ROS such as H_2_O_2 _can act on a redox-sensitive cysteine residue with a low pK_a _in the active site of PTPs (a similar condition has been described also for p21Ras, AP-1, NF-κB and hypoxia-inducible factor (HIF)-1) by oxidizing the -SH group of the active PTP. Depending on ROS levels (Figure 17), this may result in reversible as well as irreversible inactivation of PTPs, reinforcing downstream RTK signalling for variable durations. This scheme has been described also for radiation, exposure to metals, alkylating agents and environmental oxidants, and conditions that may even activate RTKs in a ligand-independent manner – 'RTK trans-activation' [102]. Changes in intracellular thiol/disulfide redox state may also affect the system since the relative, or time-limited, depletion of reducing agents may prevent reversion of oxidized/inactive PTPs to the reduced/active state.

#### ROS can activate protein kinases as well as MAPK cascades

Cytoplasm protein kinases can respond to very high levels (1 mM) of H_2_O_2 _by enhancing their activity, as shown in pioneeristic studies [14,15]. Whether these high concentrations of ROS may be reached in a biological environment is still controversial. If one refers to more realistic studies, few molecular targets and pathways have been identified to be activated by mild oxidizing conditions or by mild shifts in the intracellular thiol/disulfide redox state, including signalling components of the Src family of protein tyrosine kinases (p59^fyn ^and p56^lck^), JAK2, c-Jun amino-terminal kinases (JNKs), p38^MAPK ^and, in some cells, ERK1/2. A peculiar mechanism is the one disclosed in studies designed to analyze ASK-1 (apoptosis signalling-regulating kinase 1) activation, which, in turn, leads to activation of MKK3/6, MKK4/MKK7 and then JNKs and p38^MAPK^, finally leading to phosphorylation of ATF-2, c-Jun and p53. Elegant studies have shown that ASK-1 is usually associated with a Trx protein that binds to the amino-terminal domain of ASK-1, inhibiting its kinase activity. If ROS induce Trx dimerization and dissociation from ASK-1, this is followed by multimerization of ASK-1, activation of its kinase activity and then of the downstream signalling, leading to activation of JNKs and p38^MAPK ^[103,104].

Another interesting example of redox sensitivity is that of the serine/threonine kinase protein kinase Cα (PKC-α) [14,15]: this and other PKC isoforms are usually activated by diacylglycerol or phorbol esters, for which PKC has a binding site in an evolutionarily conserved cysteine-rich region. These PKC isoforms can be activated by ROS like H_2_O_2 _in a way that involves tyrosine phosphorylation in the catalytic domain. Interestingly, vitamin E has been described to inhibit the activity and translocation of PKC to the membrane and to be able to down-regulate some PKC-dependent responses (that is, proliferation) in target cells like smooth muscle cells; intriguingly, data on modulatory effects by vitamin E have been extended to other components of the signalling machinery, suggesting that only some of these effects may depend on the antioxidant activity of the vitamin [105].

#### ROS and oxidative stress can activate defined transcription factors

Several transcription factors can be considered as redox sensitive but the two best characterized examples are NF-κB and AP-1. NF-κB is a transcription factor shown to respond to oxidative stress [106] and it is known to be involved in inflammatory reactions, in the control of cell growth and the balance between survival and apoptosis [107] and, possibly, necrotic cell death [108] (see below). NF-κB, a definition that, in mammalian cells, includes c-Rel, RelA (p65), RelB, NF-κB1/p50 and NF-κB2/p52 proteins, which all recognise DNA sequences called κB sites [107,109,110], is also involved in maintaining mitochondrial integrity and in regulating antioxidant activity [107,110]. The redox-dependence of NF-κB relies on different mechanisms of activation that, depending on the specific target cell, may involve either an atypical phosphorylation of the Tyr42 residue of IκBα by the kinase Syk (thus, independently of IκB kinase (IKK)) or a more conventional H_2_O_2_-dependent activation through the classic IKK-dependent pathway [110], the latter being activated also by HOCl, singlet oxygen and peroxynitrite.

**Figure 18:**
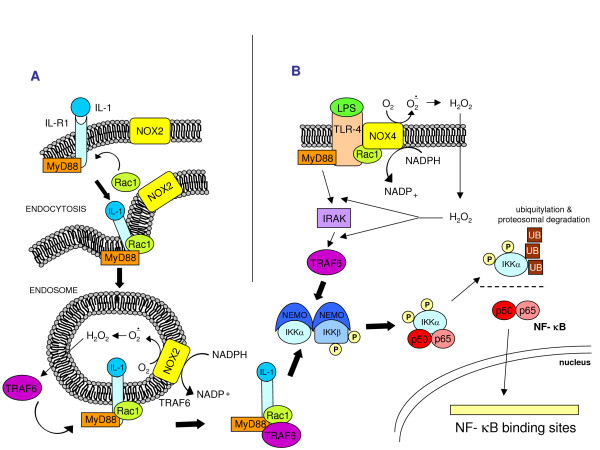
Two examples of ROS involvement in cytokine-dependent NF-κB activation. **(a) **NF-κB activation by IL-1β. In some cells IL-1β induces MyD88-dependent endocytosis of IL-R1; during endocytosis Rac1 recruits NOX2 in the endosomal compartment. NOX2 activation generates superoxide that spontaneously dismutates into H_2_O_2_, which then diffuses in the cytoplasm and triggers TRAF6 association with the ligand-receptor complex on the endosome, leading finally to NF-κB activation. **(b) **NF-κB activation by LPS. NF-κB activation by LPS through TLR4 activation involves Myd88 recruitment, which links TLR-4 activation to IRAK and TRAF6, mediating NF-κB activation. The involvement of ROS is consequent to direct interaction, followed by the Rac1-mediated activation of TLR-4 by NOX4 (or another NOX isoform, depending on the target cells). At present it is uncertain whether H_2_O_2 _operates (as for IL-1) by triggering activation of TRAF6.

**Figure 19:**
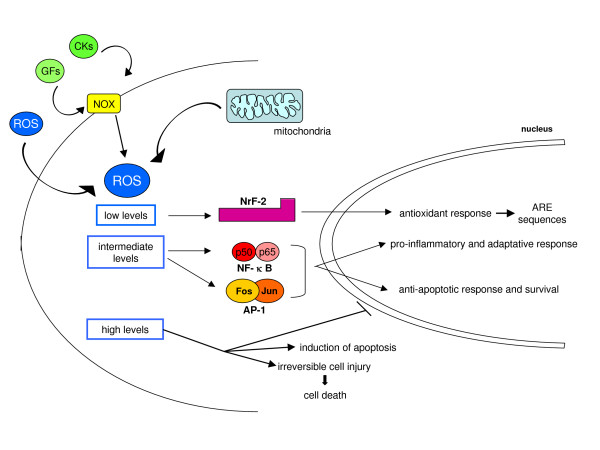
Oversimplified scheme of responses induced by increased intracellular levels of ROS.

Pertinent to this review, a general model is emerging suggesting that all cytokines leading to NF-κB activation are likely to cause intracellular generation of ROS that are then responsible for IKK activation and IκBα degradation, with IL-1, TNF and LPS being the. best characterized examples (Figure 18). The concept here is again simple: ROS, produced intracellularly as a part of the response induced by inflammatory cytokines, contribute to reinforce the signal. Figure 19 oversimplifies the concepts described above (in the 'Principles of redox homeostasis' and 'Redox sensors and the basis of redox-dependent transcriptional regulation' sections) by proposing an intuitive model that relates the levels of ROS and oxidative stress to the overall response and even fate of the target cells.

Within the same scenario one can also easily include AP-1, a dimeric (homo- or heterodimer) transcription factor typically formed from c-Jun and c-Fos and involved in several physiological and pathophysiological processes. Activation of AP-1 occurs in the presence of low levels of ROS (mainly H_2_O_2_), IL-1, UV light, and γ-irradiation. Two mechanisms may lead to redox-dependent activation of AP-1: oxidative activation of JNKs that, in turn, phosphorylate Ser63 and Ser73 of the amino-terminal transactivation domain of c-Jun, a domain that is essential for functional activation [104]; and a mild shift in the redox state by different oxidants or ROS [14,15].

A cautionary note has to be added: one should keep in mind that the DNA-binding activity of most transcription factors is redox sensitive in the opposite way. It has been shown that the binding of transcription factors to a DNA regulatory sequence requires reducing conditions because transcription factors must expose positively charged amino acid residues in their binding sites in order to be able to bind target DNA sequences (usually highly acidic and negatively charged). This introduces an apparent paradox: the binding site of a transcription factor presents redox-sensitive amino acids (cysteine, arginine) and, as is the case for NF-κB, oxidation of these critical residues may prevent its DNA-binding activity. This note is to underline that even in physiological conditions, the final response to redox changes relies on a delicate balance between pro-oxidant conditions needed to reinforce the signal and reducing conditions needed for the same signal (that is, the transcription factor) to be efficiently delivered in order to obtain the response. Pathophysiological conditions (Figures 9 and 19) can easily interfere with such a delicate balance by shifting redox homeostasis to a 'quasi-stable' but deregulated redox state.

## ROS and oxidative stress in relation to CLD aetiology

In addition to the general mechanisms able to sustain increased generation of ROS and other reactive mediators that are common to all conditions of human and experimental CLDs (that is, cell injury and death, chronic hepatitis, responses to growth factors, cyto- and chemokines, and so on), one should also consider the intrinsic contribution of the specific aetiology of a CLD. This is relevant when the primary aetiology is represented by chronic ethanol consumption, a disturbance of iron homeostasis or metabolic imbalances like those occurring in non-alcoholic fatty liver disease (NAFLD) and non-alcoholic steatohepatitis (NASH). Clinical observations have clearly established that all these three very common conditions, as independent factors, may significantly affect and accelerate fibrogenic progression of any CLD towards cirrhosis.

### Iron and its role in fibrogenic CLDs

Iron in its ferrous (Fe^2+^) or ferric (Fe^3+^) forms is critical for the life of all aerobic organisms, being included in haemoproteins like haemoglobin and myoglobin, metalloproteins, cytochromes and redox-dependent enzymes involved in oxygen and electron transport as well as in several other reactions, including oxygen sensing, NO sensing, DNA synthesis and transcriptional regulation. Major achievements in the understanding of hereditary hemocromatosis (HH) have disclosed in a detailed way [111,112] crucial molecular and cellular mechanisms responsible for the control of iron homeostasis. Iron levels are carefully controlled in terms of adsorption, stores, plasma levels and transferrin saturation, whereas major physiological pathways for its excretion are lacking in higher organisms. The literature suggests that increased levels of hepatic iron (the liver being the most relevant site of storage) can significantly contribute to fibrogenic progression of a CLD.

**Figure 20:**
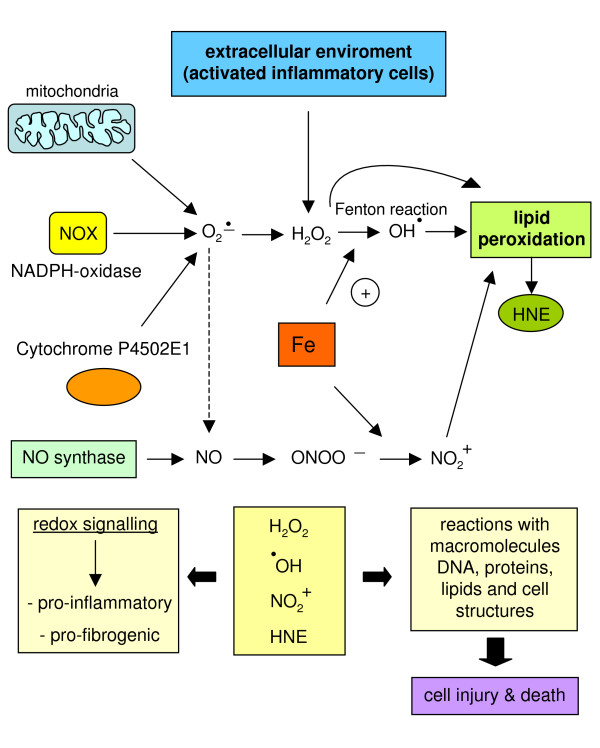
Increased levels of iron can contribute to increased generation of ROS and other radical or non-radical intermediates, resulting in a potentiation of cytotoxic, pro-inflammatory or pro-fibrogenic consequences. The role of iron in CLD progression. Hereditary hemochromatosis (HH) is a long-lasting disease in which hepatic iron levels increase progressively over a long period during which no or relatively modest inflammation and injury can be detected; when hepatic levels of iron increase to over 60 mmol/g dry weight, HSCs become activated and fibrogenesis becomes significant [113], although this transition (from non-fibrotic to fibrotic and then later cirrhotic) is not yet completely clear and other risk factors (ethanol consumption and ALD, chronic infection by HCV, concomitant metabolic conditions leading to NAFLD) are likely to be involved. However, with regard to patients with chronic HCV infection, it has been proposed that mutations in the hereditary hemochromatosis HFE gene may be responsible not only for derangement of iron homeostasis and HH, but may also worsen or accelerate the course of CLD by eliciting a turn-over of redox active iron in both the liver and plasma; in other words, the hypothesis is that HFE mutations may additionally result in increased intracellular production of ROS and free radicals taking place in hepatocytes or, also on the basis of recent knowledge on the role of hepcidin (see the section 'ROS-dependent sustained activation of JNK: a common step in oxidative stress-dependent cell death') and the iron transporter ferroportin, may affect the ability of Kupffer cells to handle and retain iron [112,114,115]. Chronic HCV infection. In addition to what has been reported for HH, it should be recalled that non-hereditary (that is, secondary) increased hepatic iron levels have been shown to represent a significant determinant for both the severity and progression rate of CLD associated with chronic HCV infection. Along these lines, different laboratories have shown a correlation between liver iron levels and HSC activation as well as fibrosis progression [114,115], which can be significantly prevented by phlebotomy. It should be noted (reviewed in [113]), however, that other researchers did not find evidence for such a correlation. NAFLD and NASH. With regard to NAFLD, current evidence suggests that metabolic disturbances leading to steatosis (as associated with obese or overweight patients and, usually, with the so-called metabolic syndrome, often also including diabetes and insulin resistance) are likely to represent the 'first hit'. In order for NAFLD to progress to non-alcoholic steatohepatitis or NASH a 'second hit' (see later) is believed to be necessary and usually identified as occurrence of oxidative stress. Along these lines, iron is a rather obvious candidate because of its well known role as an ideal metal catalyst for the generation of ROS and other free-radical or non-radical intermediates. Alcoholic liver disease (ALD). Homologous considerations (that is, the role of hepatic iron levels) may be advanced for ALD and may help to explain, at least in part, why only approximately 30% of patients with high levels of chronic alcohol consumption are likely to develop cirrhosis over time. As recently reviewed [113], there are several reasons to believe that iron is a serious and, likely, independent candidate factor able to contribute to progression of ALD to cirrhosis. For example, in the pre-cirrhotic stage, approximately 30% of ALD patients show an elevated hepatic iron index, but when the ALD progresses to cirrhosis the percentage of ALD patients having iron overload rises up to 60%. Interestingly, it was recently suggested that ethanol consumption is able to alter IL-6-dependent expression of hepcidin, a condition resulting in enhanced absorption of iron and hepatic siderosis [118]. Other mechanisms that may enhance iron hepatic levels have been recently reviewed by Brittenham [119].

Whatever the reason for increased hepatic iron levels, Figure 20 offers a simplified and 'iron-centric' view of how the metal may exacerbate oxidative stress and its consequences, which may range from redox-mediated cytotoxicity to ROS, radical and non-radical intermediate-related pro-inflammatory and pro-fibrogenic action (for more details, see [113]). Figure 20 (more details in [112-119]) also offers additional information detailing the role of excess iron in relation to HH, chronic HCV infection, NAFLD/NASH or alcoholic liver disease (ALD).

If one comes back to the central point (that is, how iron may contribute to fibrogenesis and CLD progression), several hypothesis have been proposed in which the pro-oxidant role of the metal ion remains prominent. The following concepts may be relevant: first, increased oxidative stress and lipid peroxidation have been detected in association with all major conditions of hepatic iron overload [35,113]; second, in animal models evidence suggests that antioxidant supplementation is able to significantly prevent both iron-dependent chronic liver injury and excess ECM deposition [35,120,121]; and third, iron overload may induce cytotoxicity that primarily depends on the ability to generate oxidative stress and operates by inducing mainly mitochondrial damage (including damage to mitochondrial DNA) and destabilization of lysosomal membranes [122-124].

Another concept should be recalled: intracellular levels of iron are usually carefully controlled (as in hepatocytes or macrophages) by means of iron binding to cytoplasmic Iron regulatory proteins (IRP-1 and IRP-2) and the expression of genes involved in iron homeostasis through the binding of IRPs to Iron responsive element (IRE) sequences, including those for transferrin receptor (TfR) and ferritin (Ft). An uncontrolled rise in intracellular iron may lead to the formation of a low molecular weight pool of iron potentially able to convert O_2_^•- ^and H_2_O_2 _into highly reactive •OH radicals or ferryl ions. IRPs may indeed represent a target for ROS and RNS, possibly as part of a more general scheme designed to protect the intracellular environment from oxidative stress [125]. IRP inactivation should, by down-regulating TfR expression and up-regulating Ft, decrease the intracellular labile iron pool, thus preventing amplification of iron-mediated oxidative damage. We do not know whether these regulatory mechanisms may be altered in CLDs, although it has been proposed that inactivation of IRP-2 (which is usually highly expressed in macrophages) by RNS may help to explain iron sequestration patterns of macrophages detected in tissues undergoing inflammation [125].

Copper is another transition metal acting as an excellent pro-oxidant catalyst that may contribute to enhance oxidative stress in Wilson's disease (WD), the human disease in which hepatic copper overload can occur. WD is an autosomal recessive disease caused by mutations in the gene encoding the copper-transporting P-type ATPase, ATP7B, required for copper biliary excretion [126,127]. As for iron overload, copper overload has also been described to cause hepatic oxidative stress, leading to hepatocyte injury and subcellular damage to several structures [128], although other mechanisms, either oxidative or non-oxidative, may offer significant contributions [35,36,129].

### Chronic ethanol consumption and metabolism: induction of oxidative stress and related events

Chronic ethanol consumption can lead to ALD, which encompasses a large spectrum of pathological liver changes, ranging from simple fatty liver with minimal injury to alcoholic steatohepatitis (ASH) and, in more advanced stages, fibrogenic progression to cirrhosis. Progression of ALD is now considered a multifactorial process involving nutritional, environmental and genetic factors [130], and ethanol consumption also represents one of the major host-related factors able to accelerate progression of fibrosis towards cirrhosis in chronic HCV patients [131,132] and, possibly, in patients affected by CLDs with a different aetiology. The role of ROS and oxidative stress in the pathogenesis of ethanol-induced liver injury has been extensively investigated [133-136]. Here the following relevant concepts are recalled. First, experimental and clinical data indicate that oxidative stress and lipid peroxidation are involved, with antioxidants and free radical scavengers being able, at least in animal models, to afford prevention [135]. Second, ethanol-related ROS can be produced by the mitochondria respiratory chain, ethanol metabolizing (and ethanol-inducible) cytochrome P450 2E1 (CYP2E1) in hepatocytes, but not in HSCs [137], and NOXs of activated Kupffer cells or infiltrating neutrophils [133-135]; NO produced by NO-synthase of Kupffer cells and other RNS has also been shown to contribute to ethanol-dependent hepatic injury [135,136]. The CYP2E1 isoform can also lead to generation of the ethanol-derived hydroxyethyl radical. Third, ethanol-induced oxidative stress is likely to contribute to liver steatosis found in alcoholics [138] by causing an impairment in either mitochondrial lipid oxidation [139] or lipoprotein secretions, the latter being related to enhanced degradation of ApoB100 [140] and/or oxidative alteration of lipoprotein glycosilation in Golgi apparatus [135]. Fourth, CYP-2E1 generated ROS and formation of protein adducts by lipid peroxidation products may affect proteasomal degradation leading to cytoplasmic aggregates of cytokeratins 8 and 18, leading to the formation of Mallory's bodies [141]. More features of ethanol-induced oxidative stress are presented below (see the sections 'Ethanol-related redox mechanisms leading to mitochondrial damage and hepatocyte apoptosis', 'Redox mechanisms and chronic inflammatory response in CLDs', 'Redox mechanisms in liver fibrogenesis: pro-fibrogenic cells as a functional target' and 'Redox mechanisms in immune reactions associated with CLDs: fuel for chronic inflammation and fibrogenic progression').

### ROS and oxidative stress-related reactive intermediates in NAFLD and NASH: their generation and role in causing steatosis

The term NAFLD refers to a wide spectrum of disorders having in common hepatic steatosis as a hallmark but also encompassing NASH, advanced liver fibrosis and cirrhosis. NAFLD is actually recognized as a major cause of liver-related morbidity and mortality, with a very high prevalence in Europe, USA and, more generally, in western countries (reviewed in [142-145]).

**Figure 21:**
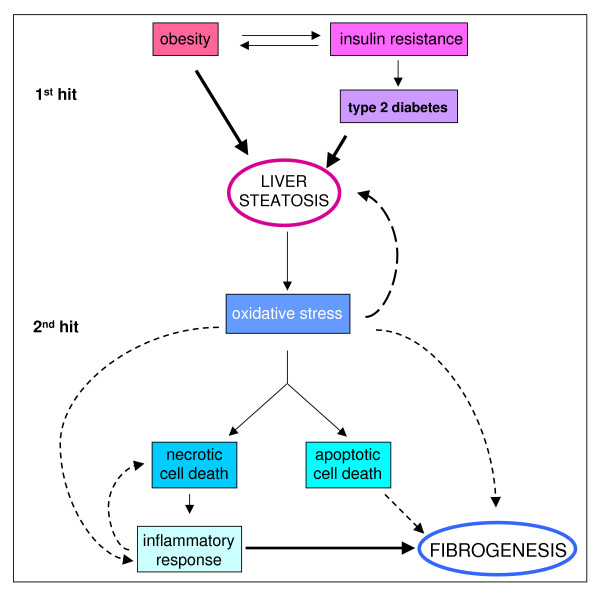
The two hit theory of NAFLD progression. Current literature indicates that the crucial derangement in NAFLD is represented by insulin resistance (IR), which is a key feature of the metabolic syndrome, a clinical entity that also includes type 2 diabetes mellitus, hypertrigliceridemia, hypertension, a decreased level of high-density lipoprotein and obesity. Indeed, as suggested by several authors, steatosis may simply represent the hepatic manifestation of the metabolic syndrome ([143,144] and references therein). The first hit is a metabolic one dominated by IR, with NAFLD being associated with both hepatic and adipose tissue IR as well as reduced insulin sensitivity of the whole human body [143,144,146]. This can lead from one side to 1) a significant reduction of glucose disposal and to a lack of suppression of hepatic glucose production as well as 2) a defect of disposal of free fatty acids (FFAs) at the adipocyte level (and skeletal muscle) that, in turn, 3) will open the way to high circulating levels of FFAs (and hypertrigliceridemia) coming from either subcutaneous and visceral fat, which will cause a persistent excess delivery of FFAs to the liver, the ultimate cause of liver steatosis [142-146]. Molecular details on how the excess load of FFAs to hepatocytes can lead to steatosis can be found in more specialized reviews [142,143], with increased *de novo *synthesis of fatty acids and triglycerides as well as both impaired oxidation (mitochondrial plus peroxisomal) and impaired efflux of fatty acids (by reduced synthesis of Apo-B100 or transport of VLDL particles) being the candidate responsible processes.

The traditional 'two-hit theory' proposed by Day and James [146] around ten years ago, which is still widely accepted [142-145], is summarized in the scheme presented in Figure 21, which also provides more details on mechanisms leading to increased circulating and hepatic levels of free fatty acids (FFAs), the ultimate cause of steatosis. There is consensus concerning the fact that long-term injury from hepatocyte triglyceride storage is responsible for the development of oxidative stress and, thus, significant intracellular generation of related reactive intermediates, with oxidative stress (detected in all clinical and experimental conditions of NAFLD/NASH [147-151]) representing the 'second hit', potentially favouring NAFLD progression. Along these lines, impairment of mitochondrial β-oxidation can lead to accumulation of FFAs in hepatocytes and FFAs are substrates (ω-oxidation) and inducers of cytochrome P450 isoforms CYP2E1 and CYP4A. Both isoforms (overexpressed in human and animal NASH) can generate ROS that, in turn, can elicit lipid peroxidation, a common hallmark of NASH [152-154]. In addition, ROS may also be generated as a consequence of increased FFA oxidation in peroxisomes (β-oxidation) by Acyl-CoA oxidase.

Other mitochondrial dysfunctions occur in NASH patients, some possibly due to up-regulation of uncoupling protein-2 (UCP-2) or representing the consequence of an oxidative stress-dependent derangement of mitochondrial membranes and/or the respiratory chain that may result in an increased release of ROS from mitochondria. The role of UCP-2 is still controversial and has recently been critically reviewed [155].

Oxidative stress and related reactive intermediates may contribute, as proposed for ethanol, to the genesis of steatosis by negatively affecting secretion of lipoproteins (either by enhancing degradation of Apo-B100 or by affecting lipoprotein glycosylation in the Golgi apparatus) [135,140] or even by interfering with the regulation of lipid synthesis by the sterol regulatory element binding protein 1 (SREBP-1) or the peroxisome proliferator-activated receptor α (PPAR- α) [156]. Moreover, ROS have been proposed to contribute to insulin resistance (IR) itself: activation of stress-activated protein kinases by ROS can lead to impairment of the correct transduction of insulin-mediated signals through the induction of serine and threonine phosphorylation of IRS-1 (insulin receptor substrate-1) and the concomitant down-regulation of IRS-1 tyrosine phosphorylation [157,158]. This has been confirmed by studies performed in hepatocytes overexpressing CYP2E1 [159].

Finally, the pivotal role of ROS in NAFLD progression has been shown by Xu *et al*. [160], who, in an elegant study (commentary in [161]), described the key role of the *Nrf-1 *gene, which is known to be involved in mediating activation of the oxidative stress-response. When using a mouse model of selective hepatic deletion of the *Nrf-1 *gene, the liver of these animals developed progressively all the characteristic features found in the progression of human NAFLD, including steatosis, apoptosis, necrosis, hepatitis, fibrosis and even liver cancer.

### Chronic HCV infection and oxidative stress

The occurrence of oxidative stress is a common finding in human patients affected by chronic HCV infection (reviewed in [162]) and is likely to rely mostly on the actual conditions of persisting liver injury and chronic inflammation. However, experimental studies have proposed that HCV core protein may induce mitochondrial injury in hepatocytes, leading to increased generation of ROS [163,164]. Mice overexpressing HCV core protein showed alterations of redox homeostasis in the absence of significant inflammation and were more susceptible to the hepatotoxin CCl_4 _[163]. Moreover, overexpression of viral proteins in these transgenic mice was found to be associated with the development of steatosis and hepatocellular carcinoma, two common features of chronic HCV infection in humans [165].

Other researchers have provided similar evidence for the HCV non-structural protein 5A (NS5A), which has been shown to associate with the membranes of endoplasmic reticulum (ER) and increase generation of ROS and activation of NF-κB and STAT-3 [166] as well as of JNKs and p38 MAPK [167]. NS5A-mediated activation of NF-κB seems to operate through tyrosine phosphorylation of IkBα and its degradation by calpain protease [168]. Thus, HCV by itself may contribute to elicit a state of oxidative and nitrosative stress in affected patients and the following major concepts, as recently reviewed [169], should be outlined. First, only some HCV-related proteins, such as NS5A, NS3 and HCV core protein, are likely to play roles in mediating redox perturbations, which can include increased generation of both ROS and RNS, iNOS and COX-2 (NS5A and C). Second, significant alterations of redox state may help to explain synergistic effects of HCV and alcohol on the progression of disease. Lastly, ROS and ethanol consumption may influence HCV replication and affect the outcome of interferon therapy.

Recently, another concept has been introduced by a study performed on transgenic mice expressing the HCV polyprotein: HCV-induced excess generation of ROS causes down-regulation of hepcidin synthesis through inhibition of the DNA binding activity of C/EBPα [170], an event able to increase iron transport from duodenum and iron release from macrophages, which lead potentially to iron overload and related consequences.

### Chronic cholestatic diseases and oxidative stress

The pattern of fibrosis in diseases of the biliary tract is peculiar and involves either significant alterations in the interactions between cholangiocytes and mesenchymal cells as well as generation of ROS and occurrence of oxidative stress. In adults and children, chronic cholestasis is usually the consequence of cholangiopathies due to autoimmunity, infectious or toxic agents, ischemia or genetically transmitted defects. All these cholangiopathies share features such as cholestasis and cholangiocyte loss associated with cholangiocyte proliferation and a variable degree of portal and periportal inflammation and fibrosis [171,172]; relevant is the 'cross-talk' between cholangiocytes, portal MFs and/or HSCs, first demonstrated for PDGF-BB and PDGF-β R [173], with cholangiocytes being able to secrete IL-6, TNF, IL-8 and MCP-1 as well as PDGF, ET-1, TGFβ2 and CTGF (reviewed in [171]).

Oxidative stress and lipid peroxidation have been detected in the most relevant clinical conditions, including primary biliary cirrhosis and the bile duct-ligation model in the rat [174-178], with antioxidant providing a significant degree of protection in animal models (reviewed in [35,36]). Generation of ROS, induction of oxidative stress and lipid peroxidation may represent the consequences of activation of inflammatory cells, as in the bile duct ligation (BDL) model [174], but ROS generation may even follow a direct effect of hydrophobic and cytotoxic bile acids on hepatocyte's mitochondria, thus mediating hepatocyte necrotic cell death and/or apoptosis [128,179-182]. Recently, it has been suggested that intracellular generation of ROS may also mediate cholangiocyte apoptosis found in primary biliary cirrhosis [178].

### Oxidative stress and genetic polymorphisms

Genetic polymorphisms may have a significant role in fibrotic progression of CLDs, as suggested by the broad spectrum of manifestations or responses that individual patients offer to the same aetiological agent or condition. Bataller and coworkers [183] have delineated a list of candidate genes involved and, not surprisingly, most, if not all, of the 'suspected' polymorphisms concern factors or enzymes that are known to be directly or indirectly either redox-sensitive or involved in the generation of ROS or other relevant reactive intermediates. The actual list includes genes encoding cytochrome P450 isoform CYP2E1, alcohol-dehydrogenases (ADHs), and Mn-SOD, as well as a number of cytokines, including TGFβ1 and TNFα.

## Redox mechanisms in the induction of cell death in CLDs: much more than a dose-dependent process

### Oxidative stress as an event able to induce cell death

Persisting liver injury and hepatocyte loss are common in CLDs and oxidative stress is likely to play a relevant role in inducing both necrotic as well as apoptotic cell death [136,184-187]. Severe oxidative stress, able to significantly damage any relevant biological macromolecule and cellular structure, is an obvious candidate to induce necrosis and may represent the outcome of severe inflammatory response following acute liver injury, with activated Kupffer cells, neutrophils or recruited mononuclear cells from peripheral blood being the 'effectors' of increased generation of reactive species. High levels of intracellular oxidative stress may also be reached within hepatocytes damaged by specific toxins (for example, CCl_4 _and acetaminophen) or aetiologies (for example, high levels of transition metal ions) or because of individual differences, including induction of peculiar cytochrome P450 isoforms (such as CYP2E1 in ASH/ALD or NASH), antioxidant status or even genetic polymorphisms.

In the scenario of a chronic inflammatory and fibrogenic disease, the first point to be discussed is the following: what do we mean, in terms of concentrations, by 'high levels of oxidative stress'? The literature offers the following considerations: reliable analytical (that is, quantitative) data on steady state concentrations of ROS as well as of other critical reactive intermediates are lacking for human liver of patients affected by a CLD; and the best data come from analyses performed on livers of animals undergoing experimental models of acute liver injury. In the liver of mice treated with acetaminophen, a model of severe and oxidative stress-dependent acute liver injury and failure, sophisticated techniques have detected a total concentration of approx 0.25 μM ROS, with H_2_O_2 _detected at levels of 0.15 μM [188]; in the CCl_4 _model of oxidative stress-related acute liver injury, intrahepatic levels of HNE reached maximal values around 10 μM [189].

Data on acute liver injury should imply that during CLDs tissue levels of ROS and other mediators are likely to be lower; this has been shown for HNE levels, which ranged from 1–3 μM in chronic CCl_4 _treatment and BDL (reviewed in [35,36]). Although several researchers have the feeling that in some conditions (that is, the environment of biological membranes within the cell, the site of active inflammation, the concomitance of more 'pro-oxidant events') ROS may locally reach levels higher then those reported in [188], these data suggest caution when interpreting results obtained *in vitro *by exposing either suspensions of freshly isolated cells or cultured cells to unrealistic concentrations of ROS or HNE, such as in the range 0.1–1.0 mM. The problem of the 'steady state' concentration of reactive intermediates is not academic: reactive intermediates of oxidative stress can induce necrotic cell death, caspase-dependent apoptosis [184-186] or other intermediate forms, including apoptosis-like or necrosis-like cell death [96], even in the presence of the same pro-oxidant agent or condition [179-182,190,191]. This is relevant in the parenchymal chronic 'battlefield': whatever the aetiology of the CLD, signs of both necrosis and apoptosis can be found in the same section, in association with the other events of the chronic scenario (inflammation, fibrogenesis, angiogenesis, and so on) [185,186]; moreover, apoptosis can indeed act in a pro-inflammatory and pro-fibrogenic way, thus sustaining progression of the CLD [192].

Some years ago, Kaplowitz [184] proposed that necrotic cell death may occur in cells exposed to very high levels of oxidative stress and be able to irreversibly damage mitochondria or to inactivate executioner caspases. This indeed may happen, as shown by *in vitro *experiments, in hepatocytes [19] or human HSC/MFs [189,191] exposed to very high concentrations of HNE (50 μM or more). However, it is not really easy sometimes to identify which mode of cell death may predominate in different conditions of liver disease. Recently, Mahli and coworkers [186] have proposed that controversies about this specific feature may be solved by recognizing that apoptosis and necrosis frequently represent alternative outcomes of the same pathways leading to cell death. For example, even in conditions able to induce caspase-dependent apoptosis (activation of either intrinsic pathways or of death receptor-related pathways), severe mitochondrial changes with membrane depolarization and uncoupling of oxidative phosphorylation may result in ATP depletion and, thus, in the blocking of caspase activation and classic apoptosis.

**Figure 22:**
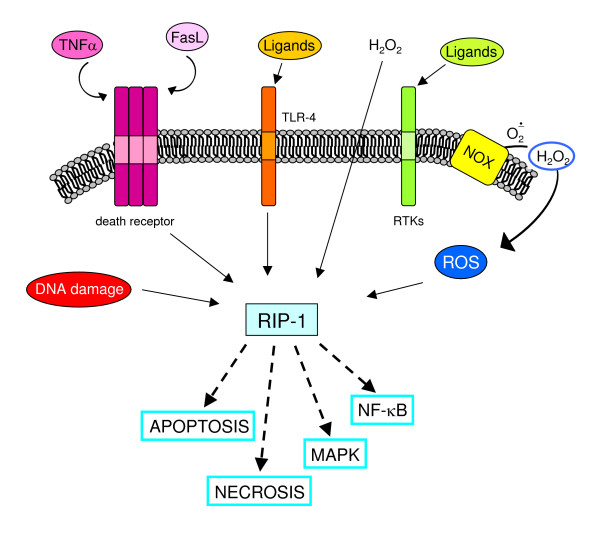
Receptor interacting protein (RIP) kinase 1 as a crucial cellular crossroads affecting whether target cells survive or die. ROS may increase in the cells also as a consequence of increased release by mithocondria, as in the case of TNFα and FasL-related responses. Activation of death receptor (DR), Toll-like receptors (TLRs) as well as signalling pathways initiated upon detection of intracellular stress (including oxidative stress itself and/or DNA damage) all have been reported to converge on RIP, particularly RIP1; the cellular context will then drive the RIP-related response of target cells towards survival by preferentially inducing activation of NF-κB and/or MAPK, or to cell death by inducing either true apoptosis or a form of caspase-independent cell death [193], although this is an oversimplified scheme (for example, sustained JNK activation is a well known event leading to cell death).

Another relevant question is, is it really necessary to reach high levels of oxidative stress to induce irreversible cell death, for example, in hepatocytes? In theory, in the scenario of a CLD, oxidative stress should be quantitatively mild to modest on a tissue basis, but it is conceivable that additive or synergic factors may overlap, leading to higher levels that may locally (for example, in the site of an inflammatory flare) or, even more likely, at the level of the single cell 'make' or 'mark' the difference for one or more cells to survive or die. Two concepts should be stressed (interested readers can find more details in [107,136,186,187,190,192-195]. First, necrosis or caspase-independent cell death is no longer seen as an accidental and uncontrolled form of cell death but is beginning to be envisaged as the result of crosstalk between several biochemical and molecular events, including an interplay between crucial signalling pathways [193]. ROS and increased levels of intracellular calcium are believed to be the main players in this scenario and necrosis, *in vivo *and *in vitro*, may function as a sort of back-up program when caspase activation is impaired. Second, both caspase-independent cell death and apoptosis, particularly when related to the engagement of death receptors (DRs) or Toll-like receptors (TLR) by their respective ligands, critically involves the kinase RIP (Receptor interacting protein), which is currently seen as one of the crucial, and redox sensitive, cellular crossroads determining whether cells live or die (Figure 22).

In the next sections a number of concepts and mechanisms involved in ROS-dependent and 'aetiology-related' forms of hepatocyte death are outlined to possible serve as a paradigm to understand the extremely complex scenarios occurring in CLDs.

### ROS and mitochondria in cell death

Alterations of mitochondria have a role in different types of either caspase-dependent or caspase-independent cell death [196] and are strictly associated with ROS. If mitochondrial integrity is deranged, this will be associated with the dissipation of the mitochondrial inner transmembrane potential (ΔΨ_m_), leading to mitochondrial outer membrane permeabilization and release of pro-apoptotic proteins such as cytochrome *c*, Smac, Diablo and AIF, leading then to apoptosis. Increased permeabilization of the mitochondrial outer membrane can also lead to increased intracellular ROS generation as a consequence of damage to the mitochondrial electron transport chain. ROS may also increase permeabilization of mitochondrial outer membrane by altering thiol groups of ANT (Adenine nucleotide translocase) or VDAC (Voltage-dependent anion channel), the latter being required for superoxide anion efflux from mitochondria. The overall message then is quite simple: mitochondria can represent not only a source of ROS but also a target for their action in relation to cell death.

### Death receptor activation, ROS, NF-κB and sustained activation of JNK: to die or not to die, that is the question

**Figure 23:**
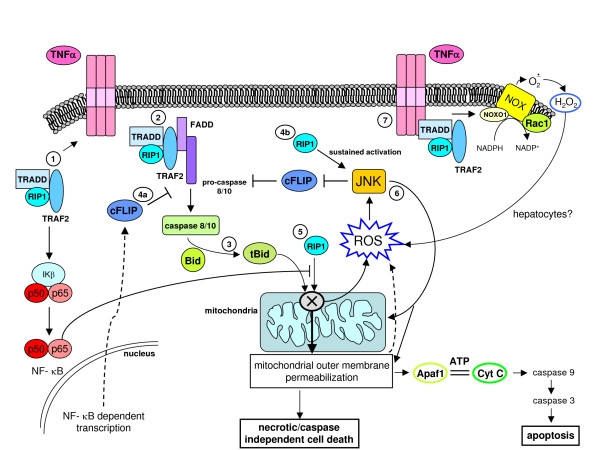
TNF-induced and ROS-dependent events in target cells: the role of JNK activation in mediating cell death. TNF interaction with its type I receptor will generate a sequence of events (depicted in Figure 23) that is not designed to lead uniquely to cell death [197-200] and in which ROS may play a crucial role. 1) The interaction between TNF and TNFRI is first followed by association with the intracytoplasmic receptor tail of the adapter TRADD (TNF-receptor associated death domain), the protein kinase RIP1 and TRAF-2 and TRAF-5 (TNF-receptor associated factors 2 and 5); this works as a signalling complex that activates NF-κB (through Inhibitor of NF-κB kinase (IKK)) and MAPK cascades that regulate the AP-1 transcription factor. Activation of NF-κB-binding activity and transcription of dependent genes is potentially pro-survival by up-regulating the synthesis of cFLIP (c FLICE inhibitory protein), which can inhibit activation of caspase 8/10, the starting point for TNF-mediated induction of classic apoptosis. 2) At this point the TRADD/RIP-1/TRAF-2 complex can dissociates from the receptor by a still unknown mechanism and bind to FADD (Fas-ligand associated death domain) to form another complex leading to recruitment of several molecules of pro-caspase 8/10 and to their autocatalytic cleavage, resulting in activation; however, this event occurs only in cells having low levels of cFLIP, suggesting that apoptosis as well as caspase-independent or necrotic cell death (see later) may occur when the preventive pro-survival function of NF-κB fails. When activation of caspase 8/10 ocurs, the scenario can still prefigure two alternatives. 3) Activated caspase 8/10 may cleave the pro-apoptotic protein Bid (to tBid), which then translocates to mitochondria, causing permeabilization of mitochondrial outer membrane, cytochrome *c *release, apoptosome assembly and activation of executioner caspases, leading to classic apoptosis. This pro-apoptotic scenario may be amplified by the recruitment to TNFRI of FAN (Factor-associated neutral sphingomyelinase), which is able to produce sphingosine that, in turn, permeabilizes lysosomal membranes, leading to the release of cathepsins. These lysosomal proteases, which can be activated following engagement of different death receptors, act as pro-apoptotic proteases able to cleave Bid as well as to induce oligomerization of Bax or interact directly with mitochondrial outer membrane (note that this pathway, for the sake of clarity, has been omitted from Figure 23). It should be noted that in hepatocytes it has been reported that generation of ROS following activation of death receptors (by Fas or TNF) is strictly dependent on Bid cleavage to tBid and its subsequent translocation to mitochondria [201]. 4) The second alternative is the one more strictly related to ROS and involves the crossroads kinase RIP1. RIP1 has multiple crucial roles; it is involved in the activation of NF-κB, but may also contribute to apoptosis when cleaved by caspase-8 to cRIP1 (4a), an event preventing or reducing activation of NF-κB and sustaining enhanced interaction between TRADD, FADD and procaspases 8/10. However, it has also been described (4b) that the kinase domain of RIP1 is essential to activate JNK, which in turn (although this is just one of the JNK-dependent actions) will favour TNFRI-related apoptosis and mitochondrial outer membrane permeabilization by eliciting cFLIP phosphorylation and degradation. Concerning the involvement of ROS, however, RIP1 (reviewed in [107,193,194]) has been shown (5) to be able also to translocate to mitochondria (prevented by NF-κB) following TNF stimulation of cells: this event induces mitochondrial outer membrane permeabilization and increases release of ROS in the cytoplasm without leading to cytochrome *c *release and apoptosome complex formation. This increased TNF-related mitochondrial generation of ROS is crucial because it can lead target cells to apoptosis or, more likely, to a necrotic or caspase-independent form of cell death: in this latter scenario a significant role is attributed to ROS-mediated sustained activation of JNK (6), surely operating in hepatocytes [186,187], which, as we will see (Figure 24), is a crucial crossroads for ROS-related irreversible injury of parenchymal cells as elicited by the different aetiologies leading to CLDs. TNF (7) may lead to ROS-dependent and JNK-mediated cell death (necrotic type) also by involving activation of Nox1: however, this mechanism has been described in fibroblasts and, at present, we do not know whether it may apply to hepatocytes or other liver cell populations [195,202].

Probably the best detailed example of ROS involvement in cell death is related to the activation of death receptors, the mitochondria-related generation of ROS and the subsequent sustained activation of JNK. Activation of pathways leading to increased mitochondrial ROS generation and related to cell death (apoptotic or not) may be elicited by TNF, mainly by acting on the TNF receptor TNFRI, but a significant role for ROS has also been described in the activation of Fas as well as other death receptors, such as DR-4 and DR-5 [107]. The reader can then easily understand the intrinsic relevance of what we are going to describe by thinking about the complex scenario of CLDs of different aetiology, where chronic inflammation and ligand-mediated activation of death receptors is a very common event (reviewed in [186]). TNF interaction with its type I receptor will generate a complex sequence of events (more detail is given in Figure 23) that are designed to lead either to survival or cell death, with ROS forcing them towards the latter [197-202]. Indeed, TNF-TNFRI interaction may serve as a paradigm for describing the role of ROS (here generated mostly by mitochondria) since (see below) several conditions leading to the increased generation of ROS will converge on sustained JNK activation and its consequences. Interestingly, TNF (Figure 23) may even lead to ROS-dependent and JNK-mediated necrotic cell death through the activation of Nox1: however, this has been described in fibroblasts and, at present, we do not know whether it may apply to hepatocytes or other liver cell populations [195,202].

### ROS-dependent sustained activation of JNK: a common step in oxidative stress-dependent cell death

**Figure 24:**
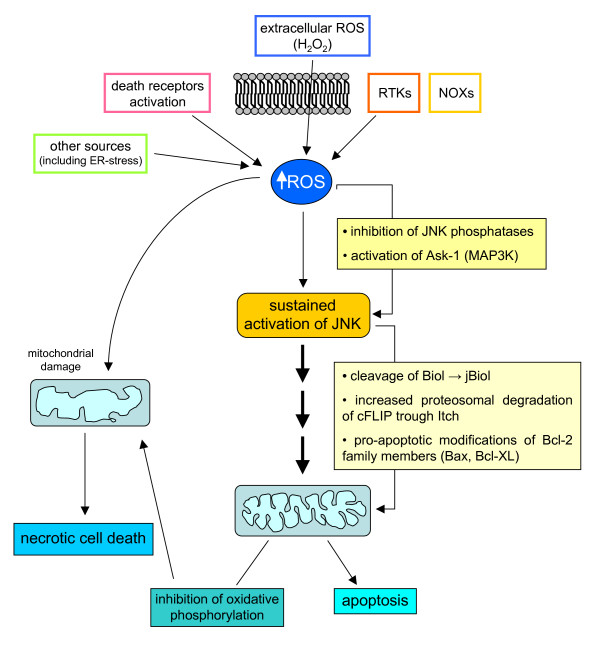
ROS-dependent sustained activation of JNK isoforms as a crucial event in inducing cell death. ROS-mediated sustained activation of JNK isoforms is likely to rely on inhibition of JNK phosphatases and/or activation of the upstream kinase ASK-1, finally resulting in mitochondrial outer membrane permeabilization. To explain this later, crucial event, the following hypotheses have been proposed: a) JNK may, in a caspase-independent way that has still not been characterized, promote the cleavage of the BH3 domain of Bid, resulting in the production of jBid, which should operate in a pro-apoptotic way similarly to tBid [209]; b) JNK may favour apoptosis by increasing proteasomal degradation of cFLIP (the inhibitor of pro-caspase 8/10 activation) by activating the ubiquitin ligase Itch [199]; c) by pro-apoptotic modifications of proteins belonging to the Bcl-2 family, such as Bax or Bcl-XL [187,194].

Mitochondria-derived ROS are implicated in TNF-dependent apoptotic and non-apoptotic cell death by inducing a ROS-dependent sustained activation of JNK (Figure 24), the latter being an event commonly seen in other conditions resulting in increased generation of ROS [107,186,187,193,199,203,204]. Several mechanisms have been described to explain ROS-mediated activation of JNK but three should be considered. The group of Karin has described for TNF-mediated cell death that ROS are able to inhibit JNK phosphatases, which are the JNK-inactivating enzymes [203,204]. Alternatively, it has been described that ROS may act by removing a thioredoxin-dependent inhibitory action on the JNK upstream kinase ASK1 (MAP3K) [104,205,206], although others [203] were not able to confirm ASK1 involvement in TNF-mediated cell death. ROS may even operate by inducing a PARP-1 over-activation resulting in depletion of NAD+ and ATP as well as in a downstream involvement of TRAF2/RIP1 mediating sustained JNK activation and cell death, but it is still unclear how TRAF2 and RIP1 may be involved following PARP-1 activation [207,208].

Whatever the mechanism, there is no doubt that ROS-dependent sustained JNK activation can promote cell death, being effective in inducing mitochondrial outer membrane permeabilization; although there is still some controversy on the molecular 'targets' of ROS-activated JNK, a number of hypotheses [187,194,199,209] have been proposed to explain this JNK-mediated event (summarized in Figure 24). The reader should also remember that NF-κB will act to prevent cell death by a number of mechanisms, some of them potentially affecting ROS-mediated sustained JNK activation, such as specifically the reported up-regulation of SOD-2 (that is, the mitochondrial SOD isoform), in TNF-dependent cell death [210]. According to the protective role of NF-κB, it has been shown that NF-κB inhibition can indeed sensitize hepatocytes to TNF-induced apoptosis by means of JNK sustained activation [211].

### Free fatty acids, endoplasmic reticulum stress, oxidative stress and cell death: hepatocyte injury in NAFLD and other CLDs

As elegantly reviewed by Parekh and Anania [143], liver injury in NAFLD can be considered essentially as the consequence of increased hepatocyte stores of FFAs. Overall, the most relevant mechanisms leading to hepatocyte injury in these metabolically altered conditions involve an excess of FFAs: directly inducing hepatocyte apoptosis and stimulating production of TNF, which is considered in this context as an adipocytokine; increasing Fas ligand binding to Fas (CD-95) receptor in steatotic hepatocytes, leading to apoptosis; leading to impaired mitochondrial or peroxisomal β-oxidation of FFAs accumulating in hepatocytes – as previously described (see the section 'ROS and oxidative stress-related reactive intermediates in NAFLD and NASH: their generation and the role in causing steatosis'), this eventually leads to generation of ROS and lipid peroxidation products, mainly HNE, which in turn may cause cell injury and death; inducing ER stress and the so-called 'unfolded protein response' (UPR), which is potentially able to result in the induction of a form of caspase-dependent cell death involving mitochondria.

**Figure 25:**
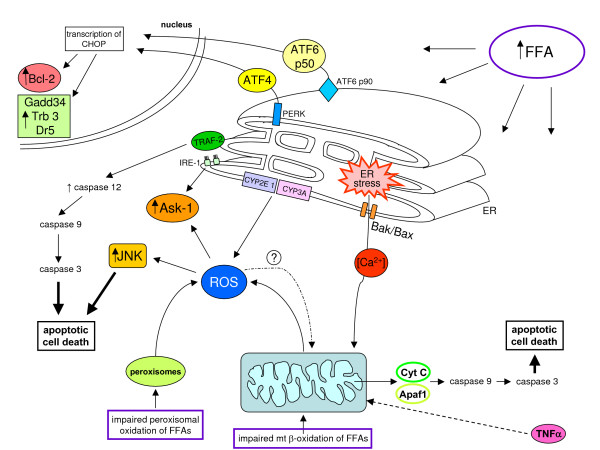
ER stress and ROS in NAFLD/NASH. This figure describes the most relevant features of ER stress following excess accumulation of FFAs in hepatocytes and also involving oxidative stress and ROS (the latter may originate in NAFLD from deranged mitochondria, CYP2E1 and CYP4A isoforms, or from peroxisomes). On the basis of what is described in the text (sections 'ROS and oxidative stress-related reactive intermediates in NAFLD and NASH: their generation and the role in causing steatosis' and 'Free fatty acids, endoplasmic reticulum stress, oxidative stress and cell death: hepatocyte injury in NAFLD but not only'), the following major features can be offered. a) When the UPR response fails to solve the problem of protein folding caused by different conditions able to induce ER stress (see text for details), including increases in FFA levels, this is followed by an induction of apoptotic cell death that can use both mitochondrial pathways as well as other independent pathways. b) ROS and oxidative stress are able to disrupt ER functions, a major cause seemingly being ROS-dependent increased release of calcium from ER stores: excess calcium has been reported to induce mitochondrial outer membrane permeabilization and, in turn, increased mitochondrial ROS release as a further contribution to increased intracellular levels by other sources and causes operating in NAFLD-related hepatocytes. c) In such a complex scenario, ER stress can result in apoptosis by a number of mechanisms, including: damage to mitochondria leading to cytochrome release, apoptosome formation and related sequential activation of executioner caspase 9 and 3; IRE-1 recruitment of TRAF-2 in order to activate either ASK-1 and then JNK, a potentially pro-apoptotic pathway that can be further sustained by ROS, or (at least in mice) caspase 12, which, in turn, can activate caspases 9 and 3; and activation of PERK and ATF6 (p90), which can lead through nuclear translocation of ATF4 and ATF6(p50), respectively, to transcriptional up-regulation of CHOP, a factor that promotes apoptosis by either inhibiting expression of Bcl-XL or up-regulating expression of pro-apoptotic proteins such as Gadd34, Trb3 and Dr5.

The first three mechanisms are all intrinsically related to ROS generation, as already described. If the interplay between FFAs, ER stress and oxidative stress is considered, the scenario can be summarized as follows (more details in [212,213]). In normal conditions the ER is the site devoted to protein entry into the secretory pathway. The ER environment involves a protein-folding machinery that is based on protein chaperones, proteins designed to catalyze folding and proteins and systems able to sense and detect unfolded or misfolded proteins. The latter systems prevent secretion of misfolded proteins and direct them to be degraded; UPR signalling pathways, specifically designed to avoid accumulation of unfolded proteins in the ER lumen, are essential for adaptation to altered homeostatic conditions, including redox changes. In this context, two concepts should be underlined: first, the ER is a unique oxygen folding environment in which disulfide bonds can be formed – unfortunately, even in normal conditions protein folding can lead to ROS generation, likely as by-products of protein oxidation occurring in the ER; and second, oxidative stress, whatever the source, can affect ER and activate UPR, the latter initially operating as an adaptative mechanism to preserve cell function and favour cell survival. Several environmental or metabolic insults may then result in an alteration of protein folding within the ER [212,213], including redox changes, depletion of calcium, energy deprivation, elevated protein traffic within the ER compartment, altered post-translational modifications and impairment of glycosylation and Golgi processing and, as also described in the case of NAFLD, excess storage of FFAs (reviewed in [143]). Indeed, signs of ER stress have been detected in hepatocytes of mice fed high fat diets or ob/ob mice (mice deficient in leptin that are obese and diabetics), including phosphorylation of pancreatic ER kinase (PERK), translation initiation factor 2 (TRAF2) and JNK. Moreover, ER stress has been shown to exacerbate hepatocyte insulin resistance [214]. Figure 25 summarizes the most relevant features of ER stress, following accumulation of FFAs and also involving oxidative stress and ROS, the latter once again significantly contributing to multiple pathways leading to hepatocyte death (for more details, see [212,213]).

ER stress-induced apoptosis has also been implicated in chronic hepatitis C and ALD [186]. With respect to HCV, several studies have shown that HCV-related proteins, including either core proteins as well as E1 and E2 proteins of the envelope, are able to induce overexpression of C/EBP homologous protein (CHOP) via the UPR response, ER depletion of calcium, and apoptosis in liver cells (HCV replicon cells) [215,216]. Moreover, ER stress and apoptosis were also found in the livers of HCV core transgenic mice [215]. More recently, it has been reported that activation of the *Gadd153 *gene by HCV in HCV replicon cells is able to sensitizes these cells to oxidant injury [217] and that both HCV and, interestingly, HBV proteins are able to induce ER stress and to up-regulate protein phosphatase 2A (PP2A) through activation of CREB [218]. In the case of ALD, ER stress involvement has been attributed to hyper-homocysteinemia, since betaine treatment in a mouse model of ALD can promote removal of homocysteine and prevent not only ER stress but also liver steatosis as well as induction of apoptosis. Excellent reviews dedicated to ER stress and HCV- or alcohol-related liver injury published recently by Kaplowitz and coworkers [219-221] also take into consideration the role of ROS.

### Ethanol-related redox mechanisms leading to mitochondrial damage and hepatocyte apoptosis

**Figure 26:**
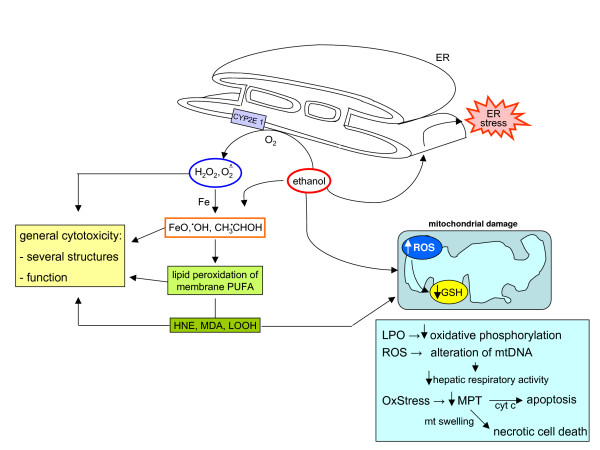
The central role of mitochondrial damage in ethanol-dependent and ROS-mediated hepatocyte injury.

This section and Figure 26 offer a number of additional concepts (see also the section 'Chronic ethanol consumption and metabolism: induction of oxidative stress and related events' above) that are mostly focussed on the role of ethanol and ROS-dependent injury of mitochondria in determining hepatocyte cell death [133-136,222]. Chronic ethanol consumption can promote intramitochondrial formation of ROS and decrease/deplete the mitochondrial content of GSH, thus making mitochondria even more susceptible to oxidative injury [223,224]. Lipid peroxidation has a major role in the impairment of mitochondrial oxidative phosphorylation [223]. Mitochondrial DNA is oxidatively altered in animals treated with ethanol [225] and this may account for the high levels of mitochondrial DNA deletions found in the liver of alcoholic patients [226] and the ethanol-related impairment of hepatic respiratory activity (presumably by affecting the synthesis of subunits of the electron transport chain encoded by mitochondrial DNA). Intramitochondrial generation of ROS and oxidative stress can induce the collapse of mitochondrial membrane potential and promote mitochondria permeability transition (MPT), possibly by favouring Bax translocation to mitochondria [225,227]; induction of MPT can, alternatively, lead to mitochondrial swelling and then to necrotic cell death as well as to cytochrome *c *release and induction of caspase-dependent apoptosis [196,228-230]. The hepatic inflammatory reaction may have a role in ethanol hepatotoxicity, with a major role attributed to activated Kupffer cells (reviewed in [133,135,231]), in which ethanol may 'switch on' a ROS- and NF-κB-related cycle leading to an amplified release of TNF; indeed, hepatocytes undergoing ethanol-induced oxidative stress may be more sensitive to the pro-apoptotic action of TNF [232], including increased sensitivity to TNF-related MTP, which may also depend on ROS- or HNE-mediated changes to ERK1/2- or PI3K-related survival signals [233,234].

### Mitochondria, nitric oxide, RNS and cell death

NO and related RNS are additional key players in the chronic inflammatory settings that characterize chronic wound healing and, more specifically, fibrogenic CLDs. NO and RNS theoretically can promote or prevent apoptotic cell death by interfering with either mitochondria-dependent or mitochondria-independent signalling pathways [235-238]. The most relevant feature is represented by the cellular redox state, with major determinants being the steady state concentration of ROS (mainly O_2_^•-^) and the rate between ROS and NO generation; as a rule, NO can act in a preventive way when ROS levels are low and *vice versa *[235-238].

Cytoprotective action is likely to rely on NO-dependent inhibition of ^•^OH generation in the presence of an iron-catalysed Fenton reaction or on inhibition of propagation of lipid peroxidation (NO being able to react with lipid alkoxyl or lipid hydroperoxyl radicals). In the presence of higher levels of ROS, the right NO/O_2_^•- ^ratio or the right levels of O_2_, NO may lead to the generation of highly reactive RNS, such as N_2_O_3 _or ONOO^-^, at levels that are able to induce more aggressive oxidation, nitrosation/S-nitrosation and nitration of different biological macromolecules, eventually leading to either necrotic or apoptotic cell death. Figure 5 summarizes these two alternative scenarios (that is, preventive versus injurious) that can be elicited by NO and RNS, with emphasis on the major molecular mechanisms that may be involved (more details in [235-243]). Here one should just recall that if liver injury is concerned, NO levels (and then those of RNS) are usually increased in CLDs as a consequence of up-regulation of iNOS in hepatocytes, endothelial cells, Kupffer cells and, possibly, HSC/MFs [235-238,241,242]. This is likely to be significantly related to levels of TNF in the chronic inflammatory environment as well as to increased translocation of gut-derived endotoxins to the portal circulation, as clearly described in the case of chronic ethanol ingestion and the subsequent interaction of endotoxins with CD14 and TLR-4 in Kupffer cells [243].

### Oxidative stress and induction of cell death in HSC/MFs

In the past decade an emerging issue in hepatology has been that liver fibrosis and even cirrhosis may be potentially reversible. Recovery from either acute or chronic liver injury in animal models is characterized by apoptosis of HSC/MFs, reduction of TIMP (tissue inhibitor of metalloproteinases) levels and progressive degradation of fibrillar fibrotic ECM. In this scenario, the sensitivity of HSCs and HSC/MFs to pro-apoptotic stimuli has been investigated to gain basic knowledge for a putative cell targeted antifibrotic therapy [79-82,91,94,95,244]. Pertinent to this review, HSC/MFs exposed to oxidative stress can undergo caspase-independent cell death but this usually requires very high concentrations of either H_2_O_2_, O_2_^•- ^or HNE, which are hardly comparable with levels observed *in vivo *[94,189,246]. This has been substantiated mainly for human cells, which, perhaps, may be more resistant than activated rat or murine cells, possibily because human HSC/MFs when activated overexpress Bcl-2 and other survival pathway proteins [94].

The overall message is that HSC/MFs are likely to survive the conditions of oxidative stress usually operating in CLDs, and rather (see below) are more likely to sustain their pro-inflammatory and pro-fibrogenic responses.

## Redox mechanisms and chronic inflammatory response in CLDs

Perpetuation of inflammatory response is a major aetiology-independent driving force for fibrogenic progression in CLDs. In such a chronic scenario, ROS and other reactive intermediates or pro-oxidants (for example, HOCl) released by activated inflammatory cells, either resident (that is, Kupffer cells) or recruited from peripheral blood, may significantly contribute to injury perpetuation. However, they may also (particularly ROS and HNE) act in a paracrine way to affect the response of surrounding cells.

Mediators of oxidative stress, whatever the source (including also ER stress [247]), the aetiology or metabolic condition can trigger or modulate expression of pro-inflammatory cytokines and chemokines in inflammatory cells and HSC/MFs, mostly through activation of NF-κB [15,107]. However, oxidative stress mediators also have a significant role in mediating the pro-apoptotic/necrotic effects of certain cytokines, with TNF being possibly the paradigm [107,186,187,248,249]. This scenario is of particular relevance in some CLDs, such as in human ALD or experimental BDL [243,250]: both ethanol ingestion or experimental cholestasis can lead to significant translocation of gut derived endotoxins to the portal circulation, where they interact with the surface receptor CD14, resulting in activation of Kupffer cells and increased synthesis of pro-inflammatory cytokines (mainly TNF), eicosanoids, and, once again, ROS and NO. With TNF being an important cause of hepatotoxicity in CLDs, one should recall that hepatocytes are resistant to the pro-apoptotic action of TNF because of the concomitant pro-survival involvement of the NF-κB and PI3K pathway: it is once again the increase in intracellular oxidative stress that can alter the balance, rendering hepatocytes more susceptible to TNF-induced cell death.

### Functional responses of inflammatory cells to oxidative stress-related intermediates

Apart from the NF-κB-related increased expression of inflammatory cytokines and chemokines [15,106,107], ROS and RNS may act as signalling intermediates by activating tyrosine kinases and inhibiting tyrosine phosphatases, resulting in an enhancement of tyrosine phosphorylation events known to regulate anti-microbial and host defence functions in leukocytes [251-253]. The following concepts should be underlined. First, ROS are likely to be involved in the process of phagocytosis, possibly by leading to amplification of the stimulating signal that follows engagement of Fc receptors on the surface of phagocytic cells, as reported for neutrophils, where ROS seem able to increase either the cross-linking of the FcãRIIIb [254], as well as by contributing, in neutrophils and macrophages, to amplification of the signal by modulating the activity of the tyrosine kinase Syk, a FcãR downstream signalling element [255]. Second, ROS-mediated inhibitory modulation of the activity of PTPs (for example, CD45, SHP-1, HePTP) has been shown to regulate signalling events involved in the activation of T lymphocytes [256,257]. In addition, CD45 has long been shown to be relevant for LTB4- and C5a-induced chemotaxis and low affinity FcãR signalling in neutrophils [258,259]: since ROS have been shown to be potent inhibitors of CD45 [260], they may interfere with physiological responses to these chemoattractants. Third, ROS may have a role in the apoptosis-related removal of leukocytes during inflammatory responses, as clearly shown for neutrophils and other cells, again by involving tyrosine phosphorylation, CD45 and SHP-1 [261-264], but also through the NOX-dependent, Lyn-mediated activation of the inositol phosphatase SHIP [265]. Similarly, peroxynitrite has also been shown to enhance apoptosis in leukocytes [266,267].

An additional concept is that intermediates able to cross the leukocyte plasma membrane may affect, in a paracrine way, the behaviour of surrounding cells, as shown originally for endothelial cells [268,269] and now accepted for many other cells with roles in chronic pro-inflammatory, angiogenic and fibrogenic environments. Accordingly, the lipid soluble aldehyde HNE, at levels compatible with those described in CLDs, up-regulates the expression of the pro-fibrogenic cytokine TGFβ1 in both rat Kupffer cells and human monocyte/macrophage cells [270]. These data may help to explain the scenario observed when using the *in vivo *experimental CCl_4_-dependent chronic model: administration of α-tocopherol to rats undergoing this protocol resulted not only in a reduction of oxidative stress, lipid peroxidation and HNE, but also in decreased synthesis of both collagen type I and TGF β1 [271,272]. Moreover, HNE and other HAKs have been reported to stimulate leukocyte chemotaxis at very low concentrations (0.1 μM; reviewed in [21,35,55]), suggesting that α-tocopherol and other chain-breaking antioxidants may prevent experimental liver fibrosis (reviewed in [21,35,36,55]) by either preventing or inhibiting selected phenotypic responses of activated MF-like cells (see the section 'Redox mechanisms in liver fibrogenesis: pro-fibrogenic cells as a functional target' below) or, at least in part, by inhibiting leukocyte recruitment due to HNE or HAKs. Indeed, both ROS and HNE have been shown to up-regulate MCP-1 expression *in vivo *and *in vitro*, and then to sustain recruitment/activation of monocytes/macrophages and Kupffer cells as well as to attract HSC/MFs [76,273,274].

### Pro-inflammatory response of activated HSC/MFs to ROS and HNE: the strange case of MCP-1

A peculiar example of interplay between the generation of reactive intermediates from oxidative stress, inflammatory response and fibrogenesis is represented by the case of MCP-1 (CCL2), which can recruit and activate monocytes and T lymphocytes and plays a major role in the formation and maintenance of the inflammatory infiltrate in different pathological conditions [275-281]. MCP-1, which is overexpressd in human CLDs and experimental models of liver injury [273,282,283], can be synthesised by activated macrophages and Kupffer cells as well as by HSC/MFs and biliary epithelial cells [283,284]. With regard to HSC/MFs, MCP-1 expression is stimulated by pro-inflammatory cytokines, such as IL-1 and TNF [282,283], thrombin [285], engagement of integrin receptors [285] and both ROS and HNE [273,286]. Indeed, as for TNF or other chemokines such as IL-8 and RANTES, MCP-1 depends on the activation of the redox sensitive transcription factors AP-1 and NF-κB [287]. In human HSC/MFs, ROS can stimulate MCP-1 expression through involvement of NF-κB, whereas HNE does not involve NF-κB and more likely operates through an AP-1-related mechanism [288], possibly involving activation of PKC [282], as shown for the PKCβ isoform in monocyte/macrophage cell lines [289].

Finally, data on human and rat fibrotic livers indicate a direct correlation between oxidative stress, hepatic levels of MCP-1 and the number of monocytes infiltrating the injured liver [273,284]. Moreover, MCP-1 can significantly stimulate chemotaxis of human HSC/MFs, another putative redox-sensitive pro-fibrogenic feature [290].

## Redox mechanisms in liver fibrogenesis: pro-fibrogenic cells as a functional target

**Figure 27:**
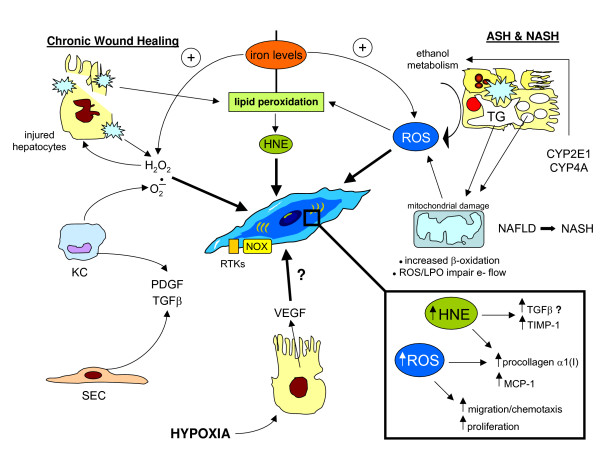
ROS and related mediators as pro-fibrogenic stimuli: a 'stellate centric' view.

In this section relevant data will be recalled (most referring to HSC/MFs) and considered within a 'myofibroblast-centric' view (Figure 27), in which pro-fibrogenic responses of MF-like cells can be affected by both extra- and intracellularly generated ROS and HNE.

### Pro-fibrogenic action of oxidative stress revealed by antifibrotic action of antioxidant molecules

The hypothesis of a causative involvement of oxidative stress in fibrogenesis relies on an impressive number of experimental studies leading to the same final scenario: antioxidant supplementation is able to significantly prevent fibrotic progression in animal models of CLDs by reducing the extent of oxidative stress and/or lipid peroxidation (reviewed in [35,36], with comments in the section 'Antioxidants as antifibrotic therapeutics for CLDs?' below). The first antioxidants used were silymarin [291,292], α-tocopherol [271,272], silybin [174] and S-adenosylmethionine [293], and most laboratories were able to describe a temporal sequence of events, suggesting overall that oxidative stress and lipid peroxidation precede or are concomitant with HSC activation and collagen deposition [294-297]. Similarly, experimental studies have also reported that NO was able to prevent both lipid peroxidation and collagen deposition [298,299].

### HNE and ROS can up-regulate pro-collagen type I expression in HSC/MFs: a puzzle that in the end makes sense

#### ROS and HNE exert a direct pro-fibrogenic action on HSC/MFs: the paracrine effect disclosed

Up-regulation of pro-collagen type I by both ROS and HNE in activated HSC/MFs is a relevant finding that has been confirmed by several different laboratories using either cells of human or rat origin. The first published study in 1993 [300] was on human HSC/MFs exposed to very low levels of HNE (1 μM), resulting in the strongly increased expression of pro-collagen type I, which was prevented by pre-treatment of cells with either vitamin E or the synthetic antioxidant diphenyl-phenylendiamine. After that study, the susceptibility of rat or human HSC/MFs in terms of pro-collagen type I synthesis to oxidative stress mediators was analysed and confirmed using different experimental strategies. The first strategy adopted in the 1990s was to expose HSC/MFs to extracellularly available mediators [189,301-305], including H_2_O_2_, O_2_^•- ^generated by the xanthine/xanthine-oxidase system, MDA, HAKs of different chain length, and the conditioned medium of normal hepatocytes undergoing oxidative stress [304,305]. Up-regulation of pro-collagen type I was also obtained later by co-culturing HSC/MFs (which do not express CYP2E1 [137]) with hepatocytes transfected to overexpress CYP2E1 and then exposed to conditions (for example, ethanol) that result in paracrine exposure of HSC/MFs to CYP2E1-dependent ROS generation [306,307]. More recently, the connection between oxidative stress, lipid peroxidation and collagen synthesis has once again been confirmed by exposing rat HSC/MFs to F_2_-isoprostanes [308]. With regard to the pro-fibrogenic mechanism, HNE has been shown in human HSC/MFs to elicit transient activation of JNK isoforms and their nuclear translocation as well as to lead to up-regulation of c-Jun and increased AP-1 binding to DNA [288]; a very similar JNK/AP-1-dependent pattern, inducing up-regulation of collagen type I, has been shown to operate in rat HSC/MFs exposed to UV irradiation [309]. The overall take-home message from these early studies was clear: ROS, mainly H_2_O_2_, HNE and HAKs of different chain length, released in a paracrine way by damaged hepatocytes, endothelial cells or activated inflammatory cells, can easily cross the membrane of HSC/MFs and lead to increased synthesis of pro-collagen type I, a significant component of the fibrillar-like ECM in fibrotic and cirrhotic livers.

#### ROS generated within HSC/MFs up-regulate collagen type I: the focus is now within the target pro-fibrogenic cell

The next step forward was provided by a series of elegant studies by Nieto and coworkers [306,310,311], who adopted the strategy of transfecting rat HSC/MFs to express human CYP2E1: pro-collagen type I transcription and synthesis in transfected cells was proportional to the levels of CYP2E1 and exacerbated by exposure of cells to ethanol or arachidonic acid (that is, conditions leading to CYP2E1-related increased generation of ROS) and prevented by using either antioxidants or specific inhibitors of CYP2E1, such as diallylsulfide. Around the same time, another part of the puzzle was revealed by showing that TGFβ1, the most potent pro-fibrogenic cytokine, was able to up-regulate collagen type I in HSC/MFs by eliciting H_2_O_2_-dependent signalling involving the binding of p35 C/EBPβ protein to a specific region of the promoter of the collagen α1(I) gene [305], an action possibly related to modulation of intracellular levels of GSH [312] and/or the involvement of p38MAPK [313]. Other signalling pathways and elements have been proposed to mediate ROS-dependent collagen type I expression, including up-regulation of cyclooxygenase 2 [311] or the redox sensitive transcription factor Sp-1 [304,314], but it should be noted that again the H_2_O_2_-dependent involvement of the same C/EBPβ protein was found also to mediate acetaldehyde (ACA)-stimulated up-regulation of collagen type I gene expression [315]. The ACA effect on HSC/MFs was biphasic, with an early phase being mediated by ACA and a late effect due to ACA-induced up-regulation of TGFβ1 expression [316]. The authors suggested in the end that ACA and TGFβ1 were eliciting similar, but not identical, mechanisms to up-regulate collagen type I expression. ACA has also been shown to up-regulate collagen type I expression through activation of JNK, similar to what was also found for HNE [288] and UV exposure [309], as finally mediated by the DNA binding protein BTEB [317]. More recently, intracellular generation of H_2_O_2 _has been shown to mediate leptin-stimulated enhancement of α 1(I) collagen gene expression in immortalized LX-2 human HSCs [318]: H_2_O_2 _was shown to activate Erk1/2 and p38MAPK through active involvement of Janus kinases 1 and 2 (JAK1 and JAK2).

If one considers that extracellular ROS, HNE and F_2_-isoprostanes are all able to up-regulate TGFβ1 synthesis in either HSC/MFs or mononuclear cells [270,308,317], another concept emerges: both the expression and activity of the pro-fibrogenic cytokine TGFβ1 are modulated by ROS or other mediators produced within the target cell, by surrounding cells (in a paracrine manner) or, as suggested for the case of ACA [316], even in an autocrine manner.

#### Intracellular generation of ROS can occur in association with cytokine-receptor interaction: NOX isoforms come into the liver fibrogenesis playground

In 2003, Bataller and coworkers [318] were the first to identify the presence of components of NOX in HSC/MFs, suggesting that the pro-fibrogenic action of Ang II, as already delineated in *in vivo *and *in vitro *studies [319,320], is dependent on the associated activation of NOX and the related ROS-dependent activation of MAPKs, phosphorylation of c-Akt and increased AP-1 DNA binding activity [318], events blocked by the specific NOX inhibitor DPI (diphenyl-phenyleneiodonium) or the inhibitor of Ang II type 1 receptor (AT1), losartan. More details on the pro-fibrogenic action of Ang II in reference to the related role of NOX can be found in specific reviews [47,48]. The activation of NOX and intracellular generation of ROS have been involved in the up-regulation of collagen type I expression in HSC/MFs after engulfment by apoptotic bodies from dead hepatocytes [97], a finding similar to an earlier report showing that macrophage engulfment of apoptotic bodies resulted in increased transcription of TGFβ1 [321]. The initial report by Canbay *et al*. [97] was followed by further studies unequivocally showing that apoptotic bodies included in HSC/MFs were signalling through NOX and ROS to up-regulate collagen α 1(I) expression [322].

### ROS and HNE affect other phenotypic responses of HSC/MFs: the specific mediator can make the difference

#### ROS, but not HNE, mediate proliferation of HSC/MFs

**Figure 28:**
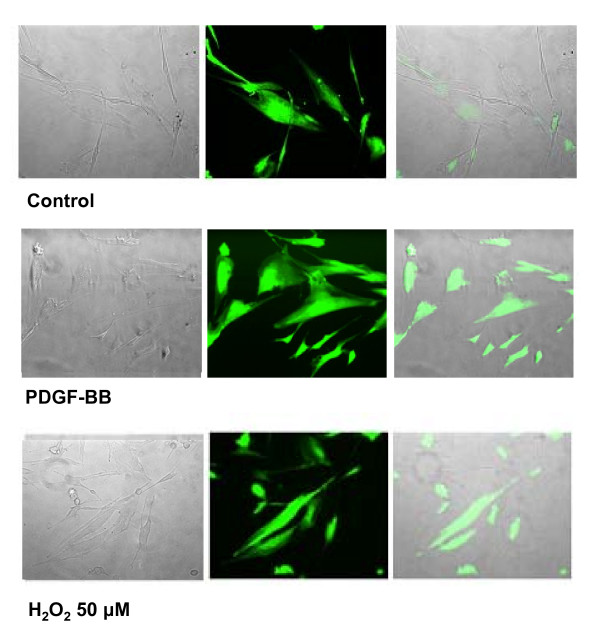
Intracellular generation of ROS in human HSC/MFs exposed to PDGF-BB or to hydrogen peroxide. Detection of intracellular generation of ROS was performed by using the conversion of 2',7'-dichlorodihydrofluorescein diacetate (DCFH-DA) probe in human HSC/MFs. Experimental conditions included control cells and cells treated with PDGF-BB (10 ng/ml) or H_2_O_2 _(50 μM, positive control) for 15 minutes. Cells were observed and photographed under a Zeiss fluorescence microscope equipped with phase contrast objectives. Images of the same fields were collected and images in the right column offer, for all conditions, the overlay of fluorescence and phase contrast images (E Novo et al, unpublished data).

Activation of HSCs as well as proliferation of HSC/MFs have been suggested to rely on the activation of NF-κB as well as on the expression of the c-*myb *proto-oncogene (reviewed in [323]). Indeed, when rat HSC/MFs are co-cultured with HepG2 cells overexpressing CYP2E1 as a source of ROS, they start to express α smooth muscle actin (α-SMA; a marker of MF-differentiation) and to proliferate [324]. These effects have been prevented by using antioxidants or inhibitors of the Na^+^/K^+ ^exchanger [323,325-328]. It has been suggested that the mitogenic action of ROS may rely on a crucial cysteine residue in Raf-1, MEK and Erk signalling elements; indeed, treatment of HSC/MFs with N-acetylcysteine is followed by activation of Erk, phosphorylation of the transcription factor Sp1 and up-regulation of p21Cip1/WAF1 expression, eventually leading to cell cycle arrest in G1 phase [329]. More recently, Adachi *et al*. [330] have proposed that the mitogenic action of PDGF-BB (see also Figure 28), and possibly also its chemotactic activity, may rely on ROS generation through involvement of NOX, leading to activation of MAPKs, particularly p38MAPK. In these experiments, performed on a human immortalised line of HSCs and on murine HSC/MFs, DPI was able to prevent the PDGF-BB-dependent proliferative response, which was restored by adding H_2_O_2 _together with PDGF-BB. Ang II has also been shown to up-regulate proliferation of HSC/MFs through involvement of NOX and ROS [318].

Results obtained with HNE as well as with other HAKs of different chain length substantially differ from those obtained with ROS: HNE and HAKs do not elicit proliferation of human HSC/MFs when employed at pro-fibrogenic doses (1–5 μM) [245,288,300,331] but rather, when used at a pro-fibrogenic dose (1 μM), they induce a block in DNA synthesis by selectively inhibiting PDGF-β receptor intrinsic tyrosine kinase activity and downstream signalling pathways [288,331]. This peculiar effect of HNE and HAKs is transient, with sensitivity to PDGF-BB being recovered within 48 hours and associated with increased expression of PDGF-β receptor subunits. Interestingly, a similar block in PDGF-dependent signalling and proliferation has been described in human cells when exposed to very high levels of ROS, either H_2_O_2 _or superoxide anion [246,331].

Such a discrepancy between the effects reported for ROS and HNE is likely to depend on the different mechanisms of action: although Uchida and coworkers [23,332] have proposed that, in some cells, HNE may result in mitochondrial damage and subsequent release of ROS (at high concentrations, however, such as 20 μM or more) or by trapping thiols, HNE is more likely to act as a non-oxidant agent by forming adducts to proteins by means of nucleophilic Michael type reactions [9,19,21], as shown in the case of JNK activation [288]. Moreover, HNE does not activate NF-κB [21,23] in human HSC/MFs [288] as ROS do, and can even inhibit c-*myb *[21], which has been involved in ROS-mediated activation of proliferation [323]. HNE exerts its pro-fibrogenic action only on fully activated human HSC/MFs [21,189] and, even more relevant, does not apparently act as an activating factor for rat HSCs early in primary culture [333]. This differs from what was suggested for ROS [75,77,80,81] and is likely to occur because quiescent HSCs can remove H_2_O_2 _less efficiently than fully activated cells [310,312], whereas HSC/MFs are more sensitive to HNE because they lack isoforms of GSH-S-transferase and aldehyde dehydrogenase able to remove or inactivate HNE [288,334]. Finally, HNE seems much more selective in its activity towards HSC/MFs, since it causes the up-regulation of only a limited number of genes, including those encoding collagen type I, TGFβ1, and TIMP-1 [21,189].

#### ROS, but not HNE, can significantly affect chemotaxis and ECM remodelling in HSC/MFs: ROS as a signal to migrate

Reports from different laboratories have shown that extracellular generation of O_2_^•- ^stimulates the migration and even invasiveness (that is, migration in matrigel) of human HSC/MFs [246,335]. Both laboratories described an O_2_^•-^-dependent stimulatory effect on the Ras/Erk pathway but opposite results for the activation of PI3K; these effects were prevented by SOD [246,335] but were not reproduced by exposing cells to H_2_O_2 _[246]. Migration, particularly in matrigel, was reported to be also dependent on superoxide-stimulated up-regulation of MMP-2 [335]. The idea that ROS may contribute to HSC/MF migration has been reinforced by studies in which Ang II [318] and PDGF-BB [330] stimulated migration in a NOX-dependent way. Recently, a ROS contribution to PDGF-dependent chemotaxis as well as the migratory response to O_2_^•- ^has been reported to critically involve JNK activation [336] (E Novo *et al*., submitted). Once again, HNE behaves in a different way since it affects neither the migration of human HSC/MFs nor the expression of MMPs (MMP-1 and MMP-2) [189], and, differently from O_2_^•-^, which has been reported to up-regulate TIMP-2 [335], it stimulates only TIMP-1 expression [189].

#### A final message from this section

The final response of a pro-fibrogenic target cell to oxidative stress is relatively unpredictable and significantly affected by a number of parameters, including: the steady state concentration of reactive species; the intrinsic state of the target cells (that is, activated versus quiescent); the presence of specific growth factors and cytokines in the microenvironment or of other cellular sources of ROS or HNE; and the concomitant generation of NO in the microenvironment, which may inhibit PDGF-BB-stimulated proliferation through enhanced synthesis of PGE2 and cAMP [337].

## Redox mechanisms in immune reactions associated with CLDs: fuel for chronic inflammation and fibrogenic progression

Several lines of evidence suggest that immune responses may have a significant role in regulating hepatic inflammation in pathological settings of CLDs. As recently reviewed for the case of ALD [249], oxidative stress can contribute to the progression of CLDs by giving rise to either oxidized or adducted epitopes able to elicit an immune response that, in turn, can contribute to perpetuation of chronic injury and progression of CLDs.

The first studies in this field were performed on ALD patients by showing the existence of circulating antibodies against different epitopes as well as infiltration of both CD4+ and CD8+ T lymphocytes in areas of inflammation and necrosis [249]. The first antibodies characterized in ALD patients were originally those directed against adducts between acetaldehyde and liver proteins [338]. However, recent studies have shown that human ALD patients exhibit significant titres of circulating antibodies directed against a number of epitopes modified by free radicals or oxidative stress reactive intermediates [249,339]. At present, the best characterized examples of these oxidized or modified epitopes, recognized by circulating antibodies (usually IgG), are: various adducts between liver proteins and hydroxyl-ethyl radicals (HERs), with circulating antibodies recognizing particularly HER-CYP2E1 adducts [249]; various proteins (mostly not identified) modified by end-products of lipid peroxidation, such as HNE, MDA or lipid hydroperoxides, with titres of related circulating antibodies more prevalent in patients with an advanced stage of ALD [340]; and lysine residues in proteins modified by the combined reaction of MDA and acetaldehyde, leading to the formation of highly antigenic products that have been defined as malonaldehyde-acetaldehyde adducts [341,342]. Indeed, as many as 35% of patients with an advanced stage of ALD (but not heavy drinkers with steatosis only) show the presence of a T cell proliferative response against lipid peroxidation protein adducts [340,343], suggesting that oxidative modifications of epitopes may promote both humoral and cellular immune responses.

Circulating IgG able to recognize epitopes modified by lipid peroxidation-derived reactive molecules has also been detected in patients with NAFLD or affected by chronic hepatitis C [249,344,345], indicating that the scenario is of more general value. When NAFLD patients were compared to control subjects, a significantly increased titre of IgG directed against protein adducted with MDA or arachidonic acid hydroperoxide or oxidized cardiolipin was found. The presence of these antibodies in NAFLD patients was independent of age, body mass index, transaminase levels, and the extent of steatosis or the concomitant presence of diabetes. Metabolic changes leading to hepatic steatosis can indeed affect immune functions, possibly by down-regulating the number of liver regulatory T cells, predominantly those with a Th1 cytokine pattern; moreover, adipokines released by adipose tissue have been suggested as additional factors able to affect the regulation of inflammatory and immune response [346,347].

A very similar scenario (that is, increased titres of IgG against human serum albumin adducted against MDA, HNE, arachidonic acid hydroperoxide and oxidized cardiolipin) has been described in patients affected by chronic hepatitis C. The titres of these antibodies were significantly increased in chronic HCV patients in relation to alcohol intake, but significant increases in these titres was reported even for those patients having only a modest alcohol intake [345]. This study also pointed out that those chronic HCV patients who were also heavy drinkers had statistically significantly more piecemeal necrosis and fibrosis than non-drinkers. Moreover, diffuse piecemeal necrosis was approximately four-fold more frequent in patients who consumed ethanol and had high titres for these antibodies than among patients whose antibody titres were within the control range. Thus, even moderate ethanol consumption can promote oxidative stress in chronic HCV patients, eliciting an immune response that is likely to contribute to the already well known ethanol-dependent worsening of the disease.

With regard to NAFLD patients, the scenario is not completely applicable, with titres of lipid peroxidation-related antibodies apparently unrelated to histological signs of necro-inflammation. However, a carefully performed statistical analysis has revealed that both titres and the frequency of these antibodies were significantly higher in NAFLD patients with bridging fibrosis or cirrhosis compared to patients with no fibrosis or just mild levels of pericellular/perilobular fibrosis [344]. This suggests that the presence of oxidative stress-triggered immune reactions could be an independent predictor of NAFLD progression to an advanced stage of fibrosis and then, likely, as a mechanism potentially contributing to NAFLD progression to NASH.

**Figure 29:**
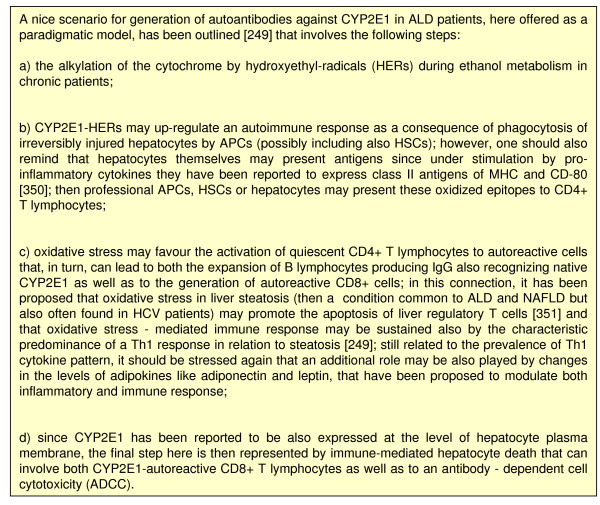
Redox-dependent development of autoantibodies against oxidative stress-modified epitopes.

Mechanisms that may be involved in the development of immune responses to oxidative stress-modified antigens are rather complex and have been recently discussed in detail for the specific case of ALD [249], with an emphasis on the unique liver immunological properties and the crucial and dual role of Kupffer cells. These resident macrophages are indeed able to down-regulate under physiological conditions antigen presentation and T cell activation by releasing TNF, IL-10 and ROS. However, they can also do the opposite in alcoholics exposed to high levels of LPS by releasing IL-12 and IL-18, which can recruit natural killer T cells as well as both CD8+ and CD4+ lymphocytes. Moreover, one should note that hepatic stellate cells may contribute to recruitment of these cells since they have been described to act also as antigen presenting cells [348,349]. The interested reader can find more details in specialized reviews [249,348-351], and Figure 29 summarizes just those mechanisms and events involved in the redox-dependent development of autoantibodies and in mediating hepatocyte cell death.

### Oxidative stress-mediated immune responses as fuel for inflammation and fibrogenesis

In CLDs, with ALD patients being a reference for the amount of evidence available, oxidized or modified epitopes may induce humoral as well as cell-mediated immune responses able to significantly contribute to the maintenance of hepatic inflammation in the natural history of ALD. This is crucial in the specific ethanol-related scenario that is often dominated by increased translocation of LPS and endotoxins from the gut to the portal circulation (and the consequent effect on Kupffer cells) and with regard to the fact that ethanol-derived oxidative stress can increase the hepatotoxic action of TNF: indeed, in either chronically ethanol fed rats or alcoholics the presence of high titres of IgG against antigens modified by lipid peroxidation or oxidative stress correlate well with increased TNF production and, quite reasonably, the severity of inflammatory infiltrate [249].

One can envisage a scenario in which, in the presence of continuous antigen stimulation, cytokines released by lymphocytes can actively sustain Kupffer cell-mediated release of cytokines and chemokines as well as of ROS and NO, which, in turn, may contribute to the perpetuation of oxidative injury, the inflammatory response and fibrogenesis. Interestingly, as many as 60–80% of ALD patients with an advanced stage of the disease have significantly increased levels of anti-phospholipid autoantibodies (aPL-Ab) that recognize oxidized cardiolipin and phosphatidylserine (reviewed in [249]). aPL-Ab, by recognizing and binding to specifically oxidized epitopes in apoptotic hepatocytes (not in living cells, with phosphatidylserine being oxidized during apoptosis and before exposure on plasma membranes), may affect the ability of Kupffer cells to recognize and phagocytose apoptotic cells. Since phagocytosis of apoptotic cells usually involves increased release of TGFβ1 and IL-10 and down-regulation of TNF and IL-12 production, an impaired disposal of apoptotic hepatocytes by Kupffer cells will negatively affect the anti-inflammatory response of Kupffer cells. Moreover, Kupffer cells as well as other phagocytes may be further activated (pro-inflammatory) by increased recognition of aPL-Ab bound to the surface of apoptotic cells through the IgG Fc receptors. Finally, impaired disposal of apoptotic cells and/or bodies may, at the same time: further increase the inflammatory response because of post-apoptotic cell lysis; maintain conditions that further sustain development of aPL-Ab; and act as a pro-fibrogenic stimulus, as shown by already cited studies in which phagocytosis of apoptotic bodies by HSC/MFs resulted in the NOX- and ROS-dependent increased stimulation of pro-collagen type I synthesis [97,322].

## Antioxidants as antifibrotic therapeutics for CLDs?

This section offers some comments on the potential usefulness of antioxidants as therapeutic agents. Since the literature on this topic is impressive, we offer here just a number of take-home messages (for more detail, see [35,36,78,352-354] and the references therein). The first message is unequivocal: antioxidants significantly prevent experimental liver fibrosis and its progression toward cirrhosis by inhibiting recruitment of inflammatory cells, the number of MFs, and levels of pro-inflammatory and pro-fibrogenic cytokines. This statement applies to different animal models of CLDs and to both naturally occurring and synthetic antioxidants that differ in structure and mechanism of action. The list of effective antioxidant strategies employed in animal models of fibrosis is impressive [35,36,77,80,352], including treatment with α-tocopherol, carotenoids, the selenium antioxidant ebselen, hydroxyl radical scavengers (such as dimethylsulphoxide of dimethylthiourea), N-acetyl-cysteine, several flavonoids and polyphenols (such as silymarin, quercetin, curcumin, epigallocatechin, and so on), the Japanese herbal medicine sho-saiko-to, the GSH-replenishing compound S-adenosyl methionine, the CYP2E1 inhibitor diallyl sulphide and the supernutrient polyenylphosphatidylcholine.

Unfortunately, results from clinical trials [35,36,78,352-354] are definitively less impressive in terms of changes in laboratory data (some trials report a significant decrease in serum alanine aminotransferase (ALT) levels and few other 'positive' features on selected liver parameters), histological appearance and survival rate. No convincing significant decrease of liver fibrosis has been documented so far, with just the possible exception of some trials with NAFLD/NASH patients treated with vitamin E ([353,354] and references therein). If one has to outline the possible reasons for such an evident discrepancy between experimental and clinical results, the following may apply. Experimental protocols have usually been designed to make the antioxidant molecule available from the beginning of the protocol, whereas in clinical trials antioxidants have been administered mainly to patients with established cirrhosis or with an advanced stage of CLD; this is relevant if one considers that oxidative stress (as stressed in this review) is likely to represent a constant pro-fibrogenic feature in the natural history of any CLD. Also, in order to match the effective antifibrotic doses employed in experimental studies, human patients should receive very high doses of these compounds, which are either intrinsically difficult to reach or, for some compounds, may raise serious toxicity concerns.

A number of strategies may help to overcome these problems. A first concept is that early diagnosis of the CLD should reasonably allow administration of safe antioxidants as soon as possible during the natural history of the disease to slow down its fibrotic progression. Alternatively, we should use strategies that lead to increased availability of candidate antioxidants with sufficiently rapid rate constants as to be pharmacologically active (that is, even at low doses). Theoretically, at least two different strategies should be tested in properly designed trials: first, the use of more powerful antioxidant molecules, such as flavonoids/polyphenols and/or active principles of herbal compounds, which may also affect fibrotic progression as a consequence of their putative 'signalling' properties able to counteract HSC activation [35,36,77,80,352,355]; and second, the use of transfection strategies to deliver antioxidant enzymes such as superoxide dismutase, thioredoxin or heme oxygenase-1 directly to the injured parenchyma or, even more specifically, to HSCs (proof of principle of the efficacy of these strategies has already been reported in experimental models [356-358]).

## Competing interests

The authors declare that they have no competing interests.

## References

[B1] Commoner B, Townsend J, Pake GE (1954). Free radicals in biological materials. Nature.

[B2] Harman D (1956). Aging: a theory based on free radical and radiation chemistry. J Gerontol.

[B3] Slater TF (1966). Necrogenic action of carbon tetrachloride in the rat: a speculative mechanism based on activation. Nature.

[B4] McCord JM, Fridovich I (1969). Superoxide dismutase: an enzymic function for erythrocuprein (hemocuprein). J Biol Chem.

[B5] Slater TF (1972). Free Radical Mechanisms in Tissue Injury.

[B6] Harman D (1981). The aging process. Proc Natl Acad Sci USA.

[B7] Cadenas E (1989). Biochemistry of oxygen toxicity. Ann Rev Biochem.

[B8] Cadenas E, Davies KJ (2000). Mitochondrial free radical generation, oxidative stress and aging. Free Radic Biol Med.

[B9] West JD, Marnett LJ (2006). Endogenous reactive intermediates as modulators of cell signaling and cell death. Chem Res Toxicol.

[B10] White AA, Crawford KM, Patt CS, Lad PJ (1976). Activation of soluble guanylate cyclase from rat lung by incubation or by hydrogen peroxide. J Biol Chem.

[B11] Mittal CK, Murad F (1977). Activation of guanylate cyclase by superoxide dismutase and hydroxyl radical: a physiological regulator of guanosine 3',5'-monophosphate formation. Proc Natl Acad Sci USA.

[B12] Ignarro LJ, Kadowitz PJ (1985). The pharmacological and physiological role of cGMP in vascular smooth muscle relaxation. Ann Pharmacol Toxicol.

[B13] Radomski MW, Palmer RMJ, Moncada S (1987). The anti-aggregating properties of vascular endothelium: interactions between prostacyclin and nitric oxide. Br J Pharmacol.

[B14] Thannickal VJ, Farnburg BL (2000). Reactive oxygen species in cell signaling. Am J Physiol Lung Cell Mol Physiol.

[B15] Dröge W (2002). Free radicals in the physiological control of cell function. Physiol Rev.

[B16] Soberman RJ (2003). The expanding network of redox signaling: new observations, complexities, and perspectives. J Clin Invest.

[B17] Chiarugi P, Cirri P (2003). Redox regulation of protein tyrosine phosphatases during receptor tyrosine kinase signal transduction. Trends Biochem Sci.

[B18] D'Autrèaux B, Toledano MB (2007). ROS as signalling molecules: mechanisms that generate specificity in ROS homeostasis. Nat Rev Mol Cell Biol.

[B19] Esterbauer H, Schaur RJ, Zollner H (1991). Chemistry and biochemistry of 4-hydroxynonenal, malonaldehyde and related aldehydes. Free Radic Biol Med.

[B20] Dianzani MU (1998). 4-Hydroxynonenal and cell signaling. Free Rad Res.

[B21] Parola M, Bellomo G, Robino G, Barrera G, Dianzani MU (1999). 4-Hydroxynonenal as a biological signal: molecular bases and pathophysiological implication. Antioxid Redox Signal.

[B22] Poli G, Schaur RJ (2000). 4-Hydroxynonenal in the phatomechanisms of oxidative stress. IUBMB Life.

[B23] Uchida K (2003). 4-Hydroxy-2-nonenal: a product and mediator of oxidative stress. Prog Lipid Res.

[B24] Witztum JL, Steinberg D (2001). The oxidative modification hypothesis of atherosclerosis: does it hold for humans?. Trends Cardiovasc Med.

[B25] Evans JL, Goldfine ID, Maddux BA, Grodsky GM (2003). Oxidative stress and stress-activated signaling pathways: a unifying hypothesis of type 2 diabetes. Endocr Rev.

[B26] Harrison D, Griendling KK, Landmesser U, Hornig B, Drexler H (2003). Role of oxidative stress in atherosclerosis. Am J Cardiol.

[B27] Pennathur S, Heinecke JW (2007). Oxidative stress and endothelial dysfunction in vascular disease. Curr Diab Rep.

[B28] Kaneto H, Katakami N, Kawamori D, Miyatsuka T, Sakamoto K, Matsuoka TA, Matsuhisa M, Yamasaki Y (2007). Involvement of oxidative stress in the pathogenesis of diabetes. Antioxid Redox Signal.

[B29] Papaharalambus CA, Griendling KK (2007). Basic mechanisms of oxidative stress and reactive oxygen species in cardiovascular injury. Trends Cardiovasc Med.

[B30] Marnett LJ, Riggins JN, West JD (2003). Endogenous generation of reactive oxidants and electrophiles and their reactions with DNA and protein. J Clin Invest.

[B31] Wu WS (2006). The signaling mechanism of ROS in tumor progression. Cancer Metastasis Rev.

[B32] Klein JA, Ackerman SL (2003). Oxidative stress, cell cycle and neurodegeneration. J Clin Invest.

[B33] Mattson MP (2006). Neuronal life-and-death signaling, apoptosis, and neurodegenerative disorders. Antioxid Redox Signal.

[B34] Zhu X, Su B, Wang X, Smith MA, Perry G (2007). Causes of oxidative stress in Alzheimer diseases. Cell Mol Life Sci.

[B35] Parola M, Robino G (2001). Oxidative stress-related molecules and liver fibrosis. J Hepatol.

[B36] Zamara E, Novo E, Parola M, Ali S, Mann DA, Friedman SL (2004). Oxidative stress and liver fibrosis: from liver injury to the modulation of cell signaling and response. Liver Diseases: Biochemical Mechanisms and New Therapeutic Insights.

[B37] Farrell GC, Larter CZ (2006). Nonalcoholic fatty liver diseases: from steatosis to cirrhosis. Hepatology.

[B38] Albano E (2006). Alcohol, oxidative stress and free radical damage. Proc Nutr Soc.

[B39] Rahman I, Biswas SK, Kode A (2006). Oxidant and antioxidant balance in the airways and airway diseases. Eur J Pharmacol.

[B40] Rahman I, Yang SR, Biswas SK (2006). Current concepts of redox signaling in the lungs. Antioxid Redox Signal.

[B41] Cho HY, Reddy SP, Kleeberger SR (2006). Nrf2 defends the lung from oxidative stress. Antioxid Redox Signal.

[B42] Halliwell B, Gutteridge JMC (1999). Free Radicals in Biology and Medicine.

[B43] Babior BM (1999). NADPH oxidase: an update. Blood.

[B44] Vignais PV (2002). The superoxide-generating NADPH oxidase: structural aspects and activation mechanism. Cell Mol Life Sci.

[B45] Genestra M (2007). Oxyl radicals, redox-sensitive signalling cascades and antioxidants. Cell Signal.

[B46] Lambeth JD (2007). Nox enzymes, ROS, and chronic disease: an example of antagonistic pleiotropy. Free Radic Biol Med.

[B47] De Minicis S, Bataller R, Brenner DA (2006). NADPH oxidase in the liver: defensive, offensive, or fibrogenic?. Gastroenterology.

[B48] De Minicis S, Brenner DA (2007). NOX in liver fibrosis. Arch Biochem Biophys.

[B49] Chiarugi P, Fiaschi T (2007). Redox signalling in anchorage-dependent cell growth. Cell Signal.

[B50] Pritsos CA (2000). Cellular distribution, metabolism and regulation of the xanthine oxidoreductase enzyme system. Chem Biol Interact.

[B51] Vazquez-Vivar J, Kalyanaramam B (2000). Generation of superoxide from nitric oxide synthase. FEBS.

[B52] Rojkind M, Domininguez-Rosales JA, Nieto N, Greenwel P (2002). Role of hydrogen peroxide and oxidative stress in healing responses. Cell Mol Life Sci.

[B53] Morrow JD, Awad JA, Kato T, Takahashi K, Badr KF, Roberts LJ, Burk RF (1992). Formation of novel non-cyclooxygenase-derived prostanoids (F2-isoprostanes) in carbon tetrachloride hepatotoxicity. J Clin Invest.

[B54] Halliwell B, Whiteman M (2004). Measuring reactive species and oxidative damage *in vivo *and in cell culture: how should you do it and what do the results mean?. Br J Pharmacol.

[B55] Comporti M, Signorini C, Arezzini B, Vecchio D, Monaco B, Gardi C (2008). Isoprostanes and hepatic fibrosis. Mol Aspects Med.

[B56] Comporti M, Signorini C, Arezzini B, Vecchio D, Monaco B, Gardi C (2008). F2-isoprostanes are not just markers of oxidative stress. Free Radic Biol Med.

[B57] Poli G, Parola M (1997). Oxidative damage and fibrogenesis. Free Radic Biol Med.

[B58] Pacher P, Beckman JS, Liaudet L (2007). Nitric oxide and peroxynitrite in health and disease. Physiol Rev.

[B59] Sase K, Michel T (1997). Expression and regulation of endothelial nitric oxide synthase. Trends Cardiovasc Med.

[B60] Archer SL, Huang JMC, Hampl V, Nelson DP, Shultz PJ, Weir EK (1994). Nitric oxide and cGMP cause vasorelaxation by activation of a charybdotoxin sensitive K-channel by a cGMP-dependent protein kinase. Proc Natl Acad Sci USA.

[B61] Beckman JS (1996). Oxidative damage and tyrosine nitration from peroxynitrite. Chem Res Toxicol.

[B62] Beckman JS, Lancaster JR, Orlando FL (1996). The physiological and pathological chemistry of nitric oxide. Nitric Oxide: Principles and Actions.

[B63] Butler AR, Megson IL, Wright PG (1998). Diffusion of nitric oxide and scavenging by blood in the vasculature. Biochim Biophys Acta.

[B64] Halliwell B, Gutteridge JMC (1989). Free Radicals in Biology and Medicine.

[B65] Young IS, Woodside JV (2001). Antioxidant in health and disease. J Clin Pathol.

[B66] Berndt C, Lillig CH, Holmgren A (2007). Thiol-based mechanisms of the thioredoxin and glutaredoxin systems: implications for diseases in the cardiovascular system. Am J Physiol Heart Circ Physiol.

[B67] Grune T, Reinheckel T, Davies KJ (1997). Degradation of oxidized proteins in mammalian cells. FASEB J.

[B68] Bader N, Grune T (2006). Protein oxidation and proteolysis. Biol Chem.

[B69] Jung T, Bader N, Grune T (2007). Oxidized proteins: intracellular distribution and recognition by the proteasome. Arc Bioch Bioph.

[B70] Wu WS, Wu JR, Hu CT (2006). Signal cross talks for sustained MAPK activation and cell migration: the potential role of reactive oxygen species. Cancer Metastasis Rev.

[B71] Liu H, Colavitti R, Rovira II, Finkel T (2005). Redox-dependent transcriptional regulation. Circ Res.

[B72] Fritz G, Grosch S, Tomicic M, Kaina B (2003). APE/Ref-1 and the mammalian response to genotoxic stress. Toxicology.

[B73] Tell G, Damante G, Caldwell D, Kelley MR (2005). The intracellular localization of APE/Ref-1: more than a passive phenomenon?. Antioxid Redox Signal.

[B74] Shafritz DA, Oertel M, Menthena A, Nierhoff D, Dabeva MD (2006). Liver stem cells and the prospects for liver reconstitution by transplanted cells. Hepatology.

[B75] Friedman SL (2000). Molecular regulation of hepatic fibrosis, an integrated cellular response to issue injury. J Biol Chem.

[B76] Pinzani M, Marra F (2001). Cytokine receptor and signalling in hepatic stellate cells. Semin Liv Dis.

[B77] Friedman SL (2003). Liver fibrosis: from bench to bedside. J Hepatol.

[B78] Pinzani M, Rombouts K (2004). Liver fibrosis-from the bench to clinical targets. Dig Liver Dis.

[B79] Bataller R, Brenner DA (2005). Liver fibrosis. J Clin Invest.

[B80] Friedman SL (2004). Mechanisms of disease: mechanisms of hepatic fibrosis and therapeutics implications. Nat Clin Pract Gastroenterol Hepatol.

[B81] Friedman SL (2008). Hepatic stellate cells: protean, multifunctional, and enigmatic cells of the liver. Physiol Rev.

[B82] Parola M, Marra F, Pinzani M (2008). Myofibroblast-like cells and liver fibrogenesis: emerging concepts in a rapidly moving scenario. Mol Asp Med.

[B83] Medina J, Arroyo AG, Sánchez-Madrid F, Moreno-Otero R (2004). Angiogenesis in chronic inflammatory liver disease. Hepatology.

[B84] Desmet VJ, Roskams T (2004). Cirrhosis reversal: a duel between dogma and myth. J Hepatol.

[B85] Cassiman D, Libbrecht L, Desmet V, Denef C, Roskams T (2002). Hepatic stellate cells/myofibroblast subpopulation in fibrotic human and rat livers. J Hepatol.

[B86] Cassiman D, Roskams T (2002). Beauty is in the eye of the beholder: emerging concepts and pitfalls in hepatic stellate cells research. J Hepatol.

[B87] Forbes SJ, Russo FP, Rey V, Burra P, Rugge M, Wright NA, Alison MR (2004). A significant proportion of myofibroblasts are of bone marrow origin in human liver fibrosis. Gastroenterology.

[B88] Russo FP, Alison MR, Bigger BW, Amofah E, Florou A, Amin F, Bou-Gharios G, Jeffery R, Iredale JP, Forbes SJ (2006). The bone marrow functionally contributes to liver fibrosis. Gastroenterology.

[B89] Valfrè di Bonzo L, Ferrero I, Cravanzola C, Mareschi K, Rustichell D, Novo E, Sanavio F, Cannito S, Zamara E, Bertero M, Davit A, Francica S, Novelli F, Colombatto S, Fagioli F, Parola M (2008). Human mesenchymal stem cells as a two-edged sword in hepatic regenerative medicine: engraftment and hepatocyte differentiation versus profibrogenic potential. Gut.

[B90] Forbes SJ (2008). Stem cell therapy for chronic liver disease – choosing the right tools for the job. Gut.

[B91] Iredale J (2007). Models of liver fibrosis: exploring the dynamic nature of inflammation and repair in a solid organ. J Clin Invest.

[B92] Aleffi S, Petrai I, Bertolani C, Parola M, Colombatto S, Novo E, Vizzuti F, Anania FA, Milani S, Rombouts K, Laffi G, Pinzani M, Marra F (2005). Upregulation of proinflammatory and proangiogenic cytokines by leptin in human hepatic stellate cells. Hepatology.

[B93] Novo E, Cannito S, Zamara E, Valfrè di Bonzo L, Caligiuri A, Cravanzola C, Compagnone A, Colombatto S, Marra F, Pinzani M, Parola M (2007). Proangiogenic cytokines as hypoxia-dependent factors stimulating migration of human hepatic stellate cells. Am J Pathol.

[B94] Novo E, Marra F, Zamara E, Valfrè di Bonzo L, Monitillo L, Cannito S, Petrai I, Mazzocca A, Bonacchi A, De Franco RS, Colombatto S, Autelli R, Pinzani M, Parola M (2006). Overexpression of Bcl-2 by activated human hepatic stellate cells: resistance to apoptosis as a mechanism of progressive hepatic fibrogenesis in humans. Gut.

[B95] El-Sharkawy AM, Oakley F, Mann D (2005). The role and regulation of hepatic stellate cells apoptosis in reversal of liver fibrosis. Apoptosis.

[B96] Leist M, Jäättelä M (2001). Four deaths and a funeral: from caspases to alternative mechanisms. Nat Rev Mol Cell Biol.

[B97] Canbay A, Taimr P, Torok N, Higuchi H, Friedman S, Gores GJ (2003). Apoptotic body engulfment by a human stellate cell line is profibrogenic. Lab Invest.

[B98] Canbay A, Higuchi H, Bronk SF, Taniai M, Sebo TJ, Gores GJ (2002). Fas enhances fibrogenesis in the bile duct ligated mouse: a link between apoptosis and fibrosis. Gastroenterology.

[B99] Den Hertog J, Groen A, Wijk T Van der (2005). Redox regulation of protein-tyrosine phosphatases. Arch Biochem Biophys.

[B100] Tonks KN (2005). Redox redux: revisiting PTPs and the control of cell signaling. Cell.

[B101] Tonks NK (2006). Protein tyrosine phosphatases: from genes, to function, to disease. Mol Cell Biol.

[B102] Weiss FU, Daub H, Ullrich A (1997). Novel mechanisms of RTK signal generation. Curr Opin Genet Dev.

[B103] Gotoh Y, Cooper JA (1998). Reactive oxygen species- and dimerization-induced activation of apoptosis signal-regulating kinase 1 in tumor necrosis factor-α signal transduction. J Biol Chem.

[B104] Liu H, Nishitoh H, Ichijo H, Kyriakis JM (2000). Activation of apoptosis signal-regulating kinase 1 (ASK1) by tumor necrosis factor receptor-associated factor 2 requires prior dissociation of the ASK1 inhibitor thioredoxin. Mol Cell Biol.

[B105] Zingg JM (2007). Modulation of signal transduction by vitamin E. Mol Aspects Med.

[B106] Schreck R, Baeuerle PA (1991). Reactive oxygen intermediates as apparently widely used messengers in the activation of NFκB transcription factor and HIV-1. Trends Cell Biol.

[B107] Temkin V, Karin M (2007). From death receptor to reactive oxygen species and c-Jun N-terminal protein kinase: the receptor-interacting protein 1 odyssey. Immunol Rev.

[B108] Luo JL, Kamata H, Karin M (2005). IKK/NF-kappaB signaling: balancing life and death – a new approach to cancer therapy. J Clin Invest.

[B109] Bonizzi G, Karin M (2004). The two NF-kappaB activation pathways and their role in innate and adaptative immunity. Trends Immunol.

[B110] Gloire G, Legrand-Poels S, Piette J (2006). NF-κB activation by reactive oxygen species: fifteen years later. Biochem Pharmacol.

[B111] Pietrangelo A (2004). Hereditary hemochromatosis-a new look at an old disease. N Engl J Med.

[B112] Pietrangelo A (2007). Hemochromatosis: an endocrine liver disease. Hepatology.

[B113] Philippe MA, Ruddell RG, Ramm GA (2007). Role of Iron in hepatic fibrosis: one pieces in the puzzle. World J Gastroenterol.

[B114] Pietrangelo A (2003). Hemochromatosis. Gut.

[B115] Pietrangelo A (2003). Hemochromatosis gene modifies corse of hepatitis C viral infection. Gastroenterology.

[B116] Martinelli AL, Ramalho LN, Zucoloto S (2004). Hepatic stellate cells in hepatitis C patients: relationship with liver iron deposits and severity of liver disease. J Gastroenterol Hepatol.

[B117] Rigamonti C, Andorno S, Maduli E, Morelli S, Pittau S, Nicosia G, Boldorini R, Sartori M (2002). Iron, hepatic stellate cells and fibrosis in chronic hepatitis C. Eur J Clin Invest.

[B118] Bridle K, Cheung TK, Murphy T, Walters M, Anderson G, Crawford DG, Fletcher LM (2006). Hepcidin is down-regulated in alcoholic liver injury: implications for the pathogenesis of alcoholic liver disease. Alcohol Clin Exp Res.

[B119] Brittenham GM (2003). Iron chelators and iron toxicity. Alcohol.

[B120] Pietrangelo A, Borella F, Casalgrandi G, Montosi G, Ceccarelli D, Gallesi D, Giovannini F, Gasparetto A, Masini A (1995). Antioxidant activity of sylibin *in vivo *during chronic iron overload in rats. Gastroenterology.

[B121] Pietrangelo A, Gualdi R, Casalgrandi G, Montosi G, Venturi E (1995). Molecular and cellular aspects of iron-induced hepatic cirrhosis in rodents. J Clin Invest.

[B122] Eaton JW, Qian M (2002). Molecular bases of cellular iron toxicity. Free Radic Biol Med.

[B123] Papanikolaou G, Pantopoulos K (2005). Iron metabolism and toxicity. Toxicol Appl Pharmacol.

[B124] Ramm GA, Ruddell RG (2005). Hepatotoxicity of iron overload: mechanisms of iron-induced hepatic fibrogenesis. Semin Liver Dis.

[B125] Cairo G, Recalcati S, Pietrangelo A, Minotti G (2002). The iron regulatory proteins: targets and modulators of free radical reactions and oxidative damage. Free Radic Biol Med.

[B126] Llanos RM, Mercer JF (2002). The molecular basis of copper homeostasis in copper-related disorders. DNA Cell Biol.

[B127] Tao TY, Gitlin JD (2003). Hepatic copper metabolism: insights from genetic disease. Hepatology.

[B128] Sokol RJ, Winklhofer-Roob BM, Deveraux MW, McKim JM (1995). Generation of hydroperoxides in isolated rat hepatocytes and hepatic mitochondria exposed to hydrophobic bile acids. Gastroenterology.

[B129] Novo E, Zamara E, Valfre di Bonzo L, Parola M, Burns JL Modulation of cell death, signal transduction and cell response by oxidative stress in the progression of chronic liver diseases. Liver Cirrhosis Research.

[B130] Day CP (2006). Genes or environment to determine alcoholic liver disease and non-alcoholic fatty liver disease. Liver Int.

[B131] Boyer N, Marcellin P (2000). Pathogenesis, diagnosis and management of hepatitis C. J Hepatol.

[B132] Poynard T, Ratziu V, Charlotte F, Goodman Z, McHutchinson J, Albrecht J (2001). Rates and risk factors of liver fibrosis progression in patients with chronic hepatitis C. J Hepatol.

[B133] Arteel GE (2003). Oxidant and antioxidant in alcohol-induced liver disease. Gastroenterology.

[B134] Nanji AA (2004). Role of different dietary fatty acids in the pathogenesis of experimental alcoholic liver disease. Alcohol.

[B135] Albano E (2008). Oxidative mechanisms in the pathogenesis of alcoholic liver disease. Mol Aspects Med.

[B136] Dey A, Cederbaum AI (2006). Alcohol and oxidative liver injury. Hepatology.

[B137] Parola M, Robino G, Bordone R, Leonarduzzi G, Casini A, Pinzani M, Neve E, Bellomo G, Dianzani MU, Ingelman-Sundberg M, Albano E (1997). Detection of cytochrome P450A (CYP3A) in human hepatic stellate cells. Biochem Biophys Res Commun.

[B138] Powell EE, Jonsson JR, Clouston AD (2005). Steatosis: co-factor in other liver diseases. Hepatology.

[B139] Pessayre D, Fromenty B (2005). NASH: a mithocondrial disease. J Hepatol.

[B140] Pan M, Cederbaum AI, Zhang YL, Ginsberg HN, Williams KJ, Fisher EA (2004). Lipid peroxidation and oxidant stress regulate hepatic apolipoprotein B degradation and VLDL production. J Clin Invest.

[B141] Bardag-Gorce F, French BA, Nan L, Song H, Nguyen SK, Yong H, Dede J, French SW (2006). CYP2E1 induced by ethanol causes oxidative stress, proteasome inhibition and cytokeratin aggresome (Mallory body-like) formation. Exp Mol Pathol.

[B142] Angulo P (2002). Nonalcoholic fatty liver disease. N Engl J Med.

[B143] Parekh S, Anania FA (2007). Abnormal lipid and glucose metabolism in obesity: implications for nonalcoholic fatty liver disease. Gastroenterology.

[B144] Marchesini G, Bugianesi E, Forlani G, Cerrelli F, Lenzi M, Manini R, Natale S, Vanni E, Villanova N, Melchionda N, Rizzetto M (2003). Nonalcoholic fatty liver, steatohepatitis, and the metabolic sindrome. Hepatology.

[B145] Tilg H, Hotamisligil GS (2006). Nonalcoholic fatty liver disease: cytokine-adipokine interplay and regulation of insulin resistance. Gastroenterology.

[B146] Day CP, James OF (1998). Steatohepatitis: a tale of two 'hits'?. Gastroenterology.

[B147] Bugianesi E, Gastaldelli A, Vanni E, Gambino R, Cassader M, Baldi S, Ponti V, Pagano G, Ferrannini E, Rizzetto M (2005). Insulin resistance in non-diabetic patients with non-alcoholic fatty liver disease: sites and mechanisms. Diabetologia.

[B148] Reid AE (2001). Nonalcoholic steatohepatitis. Gastroenterology.

[B149] Robertson G, Leclercq I, Farrell GC (2001). Nonalcoholic steatosis and steatohepatitis. II. Cytochrome P-450 enzymes and oxidative stress. Am J Physiol Gastrointest Liver Physiol.

[B150] Mehta K, Van Thiel DH, Shah N, Mobarhan S (2002). Nonalcoholic fatty liver disease: pathogenesis and the role of antioxidants. Nutr Rev.

[B151] Day CP (2002). Non-alcoholic steatohepatitis (NASH): where are we now and where are we going?. Gut.

[B152] Leclerq IA, Farrell GC, Field J, Bell DR, Gonzalez FJ, Robertson GR (2000). CYP2E1 and CYP4A as microsomal catalysts of lipid peroxides in murine non alcoholic steatohepatitis. J Clin Invest.

[B153] Weltman MD, Farrell GC, Hall P, Ingelman-Sundberg M, Liddle C (1998). Hepatic cytochrome P4502E1 is increased in patients with nonalcoholic steatohepatitis. Hepatology.

[B154] Browning JD, Horton JD (2004). Molecular mediators of hepatic steatosis and liver injury. J Clin Invest.

[B155] Serviddio G, Sastre J, Bellanti F, Vina J, Vendemmiale G, Altomare E (2008). Mitochondrial involvement in non-alcoholic steatohepatitis. Mol Asp Med.

[B156] Crabb DW, Liangpunsakul S (2006). Alcohol and lipid metabolism. J Gastroenterol Hepatol.

[B157] Houstis N, Rosen ED, Lander ES (2006). Reactive oxygen species have a casual role in multiple forms of insulin resistance. Nature.

[B158] Bloch-Damti A, Basham N (2005). Proposed mechanisms for the induction of insulin resistance by oxidative stress. Antioxid Redox Signal.

[B159] Shattemberg JM, Wang Y, Singh R, Rigoli RM, Czaja MJ (2005). Hepatocyte CYP2E1 overexpression and steatohepatitis lead to impaired hepatic insulin signalling. J Biol Chem.

[B160] Xu Z, Chen L, Leung L, Yen TSB, Lee C, Chan JY (2005). Liver-specific inactivation of the Nrf1 gene in adult mouse leads to nonalcoholic steatohepatitis and hepatic neoplasia. Proc Natl Acad Sci USA.

[B161] Parola M, Novo E (2005). Nrf1 gene expression in the liver: a single gene linking oxidative stress to NAFLD, NASH and hepatic tumours. J Hepatol.

[B162] Loguercio C, Federico A (2003). Oxidative stress in viral and alcoholis hepatitis. Free Radic Biol Med.

[B163] Moriya K, Nakagawa K, Santa T, Shintani Y, Fujie H, Miyosh H, Tsutsumi T, Miyazawa T, Ishibashi K, Horie T, Imai K, Todoroki T, Kimura S, Koike K (2001). Oxidative stress in the absence of inflammation in a mouse model for hepatitis C virus-associated hepatocarcinogenesis. Cancer Res.

[B164] Okuda M, Li K, Beard MR, Showalter LA, Scholle F, Lemon SM, Weinman SA (2002). Mitochondrial injury, oxidative stress, and antioxidant gene expression are induced by hepatitis C virus core protein. Gastroenterology.

[B165] Lerat H, Honda M, Beard MR, Loesch K, Sun J, Yang Y, Okuda M, Gosert R, Xiao SY, Weinman SA, Lemon SM (2002). Steatosis and liver cancer in transgenic mice expressing the structural and non-structural proteins of hepatitis C virus. Gastroenterology.

[B166] Gong G, Waris G, Tanveer R, Siddiqui A (2001). Human hepatitis C virus NS5A protein alters intracellular calcium levels, induces oxidative stress, and activates STAT-3 and NF-kappa B. Proc Natl Acad Sci USA.

[B167] Qadri I, Iwahashi M, Capasso JM, Hopkin MW, Flores S, Schaack J, Simon FR (2004). Induced oxidative stress and activated expression of manganese superoxide dismutase (MnSOD) during hepatitis C virus replication: Role of JNK, p38MAPK AP-1. Biochem J.

[B168] Waris G, Livolsi A, Imbert V, Peyron JF, Siddiqui A (2003). Hepatitis C virus NS5A and subgenomic replicon activate NF-kappaB via tyrosine phosphorylation of IkBα and its degradation by calpain protease. J Biol Chem.

[B169] Seronello S, Sheikh MY, Choi J (2007). Redox regulation of hepatitis C in nonalcoholic and alcoholic liver. Free Rad Biol Med.

[B170] Nishina S, Hino K, Korenaga M, Vecchi C, Pietrangelo A, Mizukami Y, Furutani T, Sakai A, Okuda M, Hidaka I, Okita K, Sakaida I (2008). Hepatitis C virus-induced reactive oxygen species raise hepatic iron level in mice by reducing hepcidin transcription. Gastroenterology.

[B171] Alpini G, McGill JM, La Russo NF (2002). The pathobiology of biliary epithelia. Hepatology.

[B172] Xia X, Demorrow S, Francis H, Glaser S, Alpini G, Marzioni M, Fava G, Lesage G (2007). Cholangiocyte injury and ductopenic syndromes. Semin Liver Dis.

[B173] Grappone C, Pinzani M, Parola M, Pellegrini G, Caligiuri A, DeFranco R, Marra F, Herbst H, Alpini G, Milani S (1999). Expression of platelet-derived growth factor in newly formed cholangiocytes during experimental biliary fibrosis in rats. J Hepatol.

[B174] Parola M, Leonarduzzi G, Robino G, Albano E, Poli G, Dianzani MU (1996). On the role of lipid peroxidation in the pathogenesis of liver damage induced by long-standing cholestasis. Free Radic Biol Med.

[B175] Kitada T, Seki S, Iwai S, Yamada T, Sakaguchi H, Wakasa K (2001). *In situ *detection of oxidative DNA damage, 8-hydroxy-deoxyguanosine, in chronic human liver disease. J Hepatol.

[B176] Tsuneyama K, Harada K, Kono N, Sasaki M, Saito T, Gershwin ME, Ikemoto M, Aria H, Nakanuma Y (2002). Damaged interlobular bile ducts in primary biliary cirrhosis show reduced expression of glutathione-S-transferase-pi and aberrant expression of 4-hydroxynonenal. J Hepatol.

[B177] Aboutwerat A, Pemberton PW, Smith A, Burrows PC, McMahon RF, Jain SK, Warnes TW (2003). Oxidant stress is a significant feature of primary biliary cirrhosis. Biochim Biophys Acta.

[B178] Salunga TL, Cui ZG, Shimoda S, Zheng HC, Nomato K, Kondo T, Takano Y, Selmi C, Alpini G, Gershwin ME, Tsuneyama K (2007). Oxidative-stress-induced apoptosis of bile duct cells in primary biliary cirrhosis. J Autoimmun.

[B179] Yerushalmi B, Dahl R, Devereaux MW, Gumpricht E, Sokol RJ (2001). Bile acid-induced rat hepatocyte apoptosis is inhibited by antioxidants and blockers of the mitochondrial permeability transition. Hepatology.

[B180] Rodrigues CM, Fan G, Wong PY, Kren BT, Steer CJ (1998). Ursodeoxycholic acid may inhibit deoxycholic acid-induced apoptosis by modulating mitochondrial transmembrane potential and reactive oxygen species production. Mol Med.

[B181] Rodrigues CM, Fan G, Ma X, Kren BT, Steer CJ (1998). A novel role for ursodeoxycholic acid in inhibiting apoptosis by modulating mitochondrial membrane perturbation. J Clin Invest.

[B182] Gumpricht E, Dahl R, Yerushalmi B, Devereaux MW, Sokol RJ (2002). Nitric oxide ameliorates hydrophobic bile acid-induced apoptosis in isolated rat hepatocytes by non-mitochondrial pathways. J Biol Chem.

[B183] Bataller R, North KE, Brenner DA (2003). Genetic polymorphisms and the progression of liver fibrosis: a critical appraisal. Hepatology.

[B184] Kaplowitz N (2000). Mechanisms of liver cell injury. J Hepatol.

[B185] Jaeschke H, Gore GJ, Cederbaum AI, Hinson JA, Pessayre D, Lemasters JJ (2002). Mechanisms of hepatotoxicity. Toxicol Sci.

[B186] Mahli H, Gores GJ, Lemasters JJ (2006). Apoptosis and necrosis in the liver: a tale of two deaths?. Hepatology.

[B187] Kaplowitz N (2006). Liver biology and pathobiology. Hepatology.

[B188] Arnaiz SL, Llesuy S, Cutrin JC, Boveris A (1995). Oxidative stress by acute acetominophen administration in mouse liver. Free Radic Biol Med.

[B189] Zamara E, Novo E, Marra F, Gentilini A, Romanelli RG, Caligiuri A, Robino G, Tamagno E, Aragno M, Danni O, Autelli R, Colombatto S, Dianzani MU, Pinzani M, Parola M (2004). 4-Hydroxynonenal as a selective pro-fibrogenic stimulus for activated human hepatic stellate cells. J Hepatol.

[B190] Patel T, Bronk SF, Gores GJ (1994). Increases of intracellular magnesium promote glycodeoxycholate-induced apoptosis in rat hepatocytes. J Clin Invest.

[B191] Parola M, Robino G, Dianzani MU (1999). 4-Hydroxy-2,3-alkenals as molecular mediators of oxidative stress in the pathogenesis of liver fibrosis. Int J Mol Med.

[B192] Guicciardi ME, Gores GJ (2005). Apoptosis: a mechanism of acute and chronic liver injury. Gut.

[B193] Festjens N, Vanden Berghe T, Vandenabeele P (2006). Necrosis, a well-orchestrated form of cell demise: signalling cascades, important mediators and concomitant immune response. Biochim Biophys Acta.

[B194] Festjens N, Vanden Berghe T, Cornelis S, Vandenabeele P (2007). RIP1, a kinase on the crossroads of a cell's decision to live or die. Cell Death Diff.

[B195] Vanden Berghe T, Declercq W, Vandenabeele P (2007). NADPH oxidases: new players in TNF-induced necrotic cell death. Mol Cell.

[B196] Green DR, Kroemer G (2004). The pathophysiology of mitochondrial cell death. Science.

[B197] Micheau O, Tschopp J (2003). Induction of TNF receptor I-mediated apoptosis via two sequential signaling complexes. Cell.

[B198] Chang L, Karin M (2001). Mammalian MAP kinase signalling cascades. Nature.

[B199] Chang L, Kamata H, Solinas G, Luo JL, Maeda S, Venuprasad K, Liu YC, Karin M (2006). The E3 ubiquitin ligase itch couples JNK activation to TNFalpha-induced cell death by inducing c-FLIP(L) turnover. Cell.

[B200] Thome M, Schneider P, Hofmann K, Fickenscher H, Meinl E, Neipel F, Mattmann C, Burns K, Bodmer JL, Schröter M, Scaffidi C, Krammer PH, Peter ME, Tschopp J (1997). Viral FLICE-inhibitory proteins (FLIPs) prevent apoptosis induced by death receptors. Nature.

[B201] Ding WX, Ni HM, DiFrancesca D, Stolz DB, Yin XM (2004). Bid-dependent generation of oxigen radicals promotes death receptor activation-induced apoptosis in murine hepatocytes. Hepatology.

[B202] Kim YS, Morgan MJ, Choksi S, Liu ZG (2007). TNF-Induced activation of the Nox1 NADPH oxidase and its role in the induction of necrotic cell death. Mol Cell.

[B203] Kamata H, Honda S, Maeda S, Chang L, Hirata H, Karin M (2005). Reactive oxygen species promote TNFalpha-induced death and sustained JNK activation by inhibiting MAP kinase phosphatases. Cell.

[B204] Pham CG, Bubici C, Zazzeroni F, Papa S, Jones J, Alvarez K, Jayawardena S, De Smaele E, Cong R, Beaumont C, Torti FM, Torti SV, Franzoso G (2004). Ferritin heavy chain upregulation by NF-kappaB inhibits TNFalpha-induced apoptosis by suppressing reactive oxygen species. Cell.

[B205] Tobiume K, Matsuzawa A, Takahashi T, Nishitoh H, Morita K, Takeda K, Minowa O, Miyazono K, Noda T, Ichijo H (2001). ASK1 is required for sustained activations of JNK/p38 MAP kinases and apoptosis. EMBO Rep.

[B206] Liu Y, Min W (2002). Thioredoxin promotes ASK1 ubiquitination and degradation to inhibit ASK1-mediated apoptosis in a redox activity-independent manner. Circ Res.

[B207] Miramar MD, Costantini P, Ravagnan L, Saraiva LM, Haouzi D, Brothers G, Penninger JM, Peleato ML, Kroemer G, Susin SA (2001). NADPH oxidase activity of mitochondrial apoptosis-inducing factor. J Biol Chem.

[B208] Zangar RC, Davydov DR, Verma S (2004). Mechanisms that regulate production of reactive oxygen species by cytochrome P450. Toxicol.

[B209] Deng Y, Ren X, Yang L, Lin Y, Wu X (2003). A JNK-dependent pathway is required for TNFalpha-induced apoptosis. Cell.

[B210] Jones PL, Ping D, Boss JM (1997). Tumor necrosis factor alpha and interleukin-1beta regulate the murine manganese superoxide dismutase gene through a complex intronic enhancer involving C/EPB-beta and NF-kappaB. Mol Cell Biol.

[B211] Lin H, Chan R, Lo M, Czaja MJ (2002). NF-κB inhibition sensitizes hepatocytes to TNF-induced apoptosis through a sustained activation of JNK and c-Jun. Hepatology.

[B212] Malhotra JD, Kaufman RJ (2007). The endoplasmic reticulum and the unfolded protein response. Sem Cell Dev Biol.

[B213] Marciniak SJ, Ron D (2006). Endoplasmic reticulum stress signaling in disease. Physiol Rev.

[B214] Wei Y, Wang D, Topczewski F, Pagliassotti MJ (2006). Saturated fatty acids induce endoplasmic reticulum stress and apoptosis independently of ceramide in liver cells. Am J Physiol Endocrinol Metab.

[B215] Benali-Furet N, Chami M, Ludivine H, DeGiorgi F, Vernejoul F, Lagorce D, Buscail L, Bartenschlager R, Ichas F, Rizzuto R, Paterlini-Brechot P (2005). Hepatitis C virus core triggers apoptosis in liver cells by inducing ER stress and calcium depletion. Oncogene.

[B216] Chan SW, Egan PA (2005). Hepatitis C virus envelope proteins regulate CHOP via induction of the unfolded protein response. FASEB J.

[B217] Ciccaglione AR, Marcantonio C, Tritarelli E, Equestre M, Venditelli F, Costantino A, Geraci A, Rapicetta M (2007). Activation of the ER stress gene GADD153 by hepatitis C virus sensitized cells to oxidant injury. Virus Res.

[B218] Christen V, Treves S, Duong FH, Heim MH (2007). Activation of endoplasmic reticulum stress response by hepatitis viruses up-regulates protein phosphatases 2A. Hepatology.

[B219] Kaplowitz N, Ji C (2006). Unfolding new mechanisms of alcoholic liver disease in the endoplasmic reticulum. J Gastroenterol Hepatol.

[B220] Ji C, Kaplowitz N (2006). ER stress: can the liver cope?. J Hepatol.

[B221] Kaplowitz N, Than TA, Shinohara M, Ji C (2007). Endoplasmic reticulum stress and liver injury. Sem Liv Dis.

[B222] Yip WW, Burt AD (2006). Alcoholic liver disease. Semin Diagn Pathol.

[B223] Bailey SM, Cunningham CC (2002). Contribution of mitochondria to oxidative stress associated with alcohol liver disease. Free Rad Biol Med.

[B224] Fernandez-Checa JC, Kaplowitz N (2005). Hepatic mitochondrial glutathione: transport and role in disease and toxicity. Toxicol Appl Pharmacol.

[B225] Hoek JB, Cahill A, Pastorino JG (2002). Alcohol and mitochondria: a dysfunctional relationship. Gastroenterology.

[B226] Mansouri A, Fromenty B, Berson A, Robin MA, Grimbert S, Beaugrand M, Erlinger S, Pessayre D (1997). Multiple hepatic mitochondrial DNA deletions suggest premature oxidative aging in alcoholics. J Hepatol.

[B227] Adachi M, Higuchi H, Miura S, Azuma T, Inokuchi S, Saito H, Kato S, Ishii H (2004). Bax interacts with voltage-dependent anion channel and mediates ethanol-induced apoptosis in rat hepatocytes. Am J Physiol.

[B228] Adachi M, Ishii H (2002). Role of mitochondria in alcoholic liver injury. Free Rad Biol Med.

[B229] Caro AA, Cederbaum AI (2004). Oxidative stress, toxicology and pharmacology of CYP2E1. Ann Rev Pharmacol Toxicol.

[B230] Fromenty B, Pessayre D (1997). Impaired mitochondrial function in microvesicular steatosis. Effects of drugs, ethanol, hormones and cytokines. J Hepatol.

[B231] Hines IN, Wheeler MD (2004). Recent advances in alcoholic liver disease. III. Role of the innate immune response in alcoholic hepatitis. Am J Physiol.

[B232] Hoek JB, Pastorino JG (2004). Alcohol and mitochondria: a dysfunctional relationship. Semin Liver Dis.

[B233] Shulga N, Hoek JB, Pastorino JG (2005). Elevated PTEN levels account for the increased sensitivity of ethanol-exposed cells to tumor necrosis factor cytotoxicity. J Biol Chem.

[B234] Sampey BP, Stewart BJ, Petersen DR (2007). Ethanol-induced modulation of hepatocellular extracellular signal-regulated kinase 1/2 activity via 4-hydroxynonenal. J Biol Chem.

[B235] Li J, Billiar TR (1998). Nitric oxide. IV. Determinants of nitric oxide protection and toxicity in liver. Am J Physiol.

[B236] Clemens MG (1999). Nitric oxide in liver injury. Hepatology.

[B237] Boyd CS, Cadenas E (2002). Nitric oxide and cell signaling pathways in mitochondrial-dependent apoptosis. Biol Chem.

[B238] Kim PKM, Zuckerbraun BS, Otterbein LE, Vodovotz Y, Billiar TR (2004). Till cell death do us part: nitric oxide and mechanisms of hepatotoxicity. Biol Chem.

[B239] Vodovotz Y, Kim PKM, Bagci EZ, Ermentrout GB, Chow CC, Bahar I, Billiar TR (2004). Inflammatory modulation of hepatocytes apoptosis by nitric oxide: *in vivo*, *in vitro*, and *in silico *studies. Curr Mol Med.

[B240] Grisham MB, Jourd'Heuil D, Wink DA (1998). Nitric oxide. I. Physiological chemistry of nitric oxide and its metabolites: implication in inflammation. Am J Physiol.

[B241] Shash V, Haddad FG, Garcia-Cardena G, Frangos JA, Mennone A, Groszmann RJ, Sessa WC (1997). Liver sinusoidal endothelial cells are responsible for nitric oxide modulation of resistance in the hepatic sinusoids. J Clin Invest.

[B242] Rockey DC (2001). Hepatic blood flow regulation by stellate cells in normal and injured liver. Semis Liver Dis.

[B243] Rao RK, Seth A, Sheth P (2004). Recent advances in alcoholic liver disease. I. Role of intestinal permeability and endotoxemia in alcoholic liver disease. Am J Physiol.

[B244] Iredale JP (2001). Hepatic stellate cell behaviour during resolution of liver injury. Semin Liver Dis.

[B245] Robino G, Parola M, Marra F, Caligiuri A, De Franco RM, Zamara E, Bellomo G, Gentilini P, Pinzani M, Dianzani MU (2000). Interaction between 4-hydroxy-2,3-alkenals and the platelet-derived growth factor-beta receptor. Reduced tyrosine phosphorylation and downstream signaling in hepatic stellate cells. J Biol Chem.

[B246] Novo E, Marra F, Zamara E, Valfrè di Bonzo L, Caligiuri A, Cannito S, Antonaci C, Colombatto S, Pinzani M, Parola M (2006). Dose dependent and divergent effects of superoxide anion on cell death, proliferation, and migration of activated human hepatic stellate cells. Gut.

[B247] Malhotra JD, Kaufman RJ (2007). Endoplasmic reticulum stress and oxidative stress: a vicious cycle or a double-edged sword?. Antioxid Redox Signal.

[B248] Marra F, Gastaldelli A, Svegliati Baroni G, Tell G, Tiribelli C (2007). Molecular basis and mechanisms of progression of non-alcoholic steatohepatitis. J Mol Med.

[B249] Vidali M, Stewart FS, Albano E (2008). Interplay between oxidative stress and immunity in the progression of alcohol-mediated liver injury. Trends Mol Med.

[B250] Isayama F, Hines IN, Kremer M, Milton RJ, Byrd CL, Perry AW, McKim SE, Parsons C, Rippe RA, Wheeler MD (2006). LPS signaling enhances hepatic fibrogenesis caused by experimental cholestasis in mice. Am J Physiol.

[B251] Fialkow L, Wang Y, Downey GP (2007). Reactive oxygen and nitrogen species as signaling molecules regulating neutrophil function. Free Rad Biol Med.

[B252] Forman HJ, Torres M (2001). Redox signaling in macrophages. Mol Asp Med.

[B253] Thakur V, McMullen MR, Pritchard MT, Nagy LE (2007). Regulation of macrophage activation in alcoholic liver disease. J Gastroenterol Hepatol.

[B254] Salmon J, Millard SS, Brogle NL, Kimberly RP (1995). Fc gamma receptor IIIb enhances Fc gamma receptor Ila function in an oxidant-dependent and allele-sensitive manner. J Clin Invest.

[B255] Greenberg S, Chang P, Silverstein SC (1994). Tyrosine phosphorylation of the gamma subunit of Fc gamma receptors, p72syk, and paxillin during Fc receptor-mediated phagocytosis in macrophages. J Biol Chem.

[B256] Holmes N (2006). CD45: all is not yet crystal clear. Immunology.

[B257] Lee K, Esselman WJ (2002). Inhibition of PTPs by H_2_O_2 _regulates the activation of distinct MAPK pathways. Free Radic Biol Med.

[B258] Harvath L, Balke JA, Christiansen NP, Russell AA, Skubitz KM (1991). Selected antibodies to leukocyte common antigen (CD45) inhibit human neutrophil chemotaxis. J Immunol.

[B259] Hoffmeyer F, Witte K, Gebhardt U, Schmidt RE (1995). The low affinity Fc gamma RIIa and Fc gamma RIIIb on polymorphonuclear neutrophil are differentially regulated by CD45 phosphatase. J Immunol.

[B260] Fialkow L, Chan CK, Downey GP (1997). Inhibition of CD45 during neutrophil activation. J Immunol.

[B261] Yasui K, Kobayashi N, Yamazaki T, Agematsu K, Matsuzaki S, Ito S, Nakata S, Baba A, Koike K (2005). Superoxide dismutase (SOD) as a potential inhibitory mediator of inflammation via neutrophil apoptosis. Free Radic Res.

[B262] Melley DD, Evans TW, Quinlan GJ (2005). Redox regulation of neutrophil apoptosis and the systemic inflammatory response syndrome. Clin Sci (London).

[B263] Zhang B, Hirahashi J, Cullere X, Mayadas TN (2003). Elucidation of molecular events leading to neutrophil apoptosis following phagocytosis: cross-talk between caspase 8, reactive oxygen species, and MAPK/ERK activation. J Biol Chem.

[B264] Fortin M, Steff AM, Felberg J, Ding I, Schraven B, Johnson P, Hugo P (2002). Apoptosis mediated trough CD45 is independent of its phosphatase activity and association with leukocyte phosphatase-associated phosphoprotein. J Immunol.

[B265] Gardai S, Whitlock BB, Helgason C, Ambruso D, Fadok V, Bratton D, Henson PM (2002). Activation of SHIP by NADPH oxidase-stimulated Lyn leads to enhanced apoptosis in neutrophils. J Biol Chem.

[B266] Blaylock MG, Cuthbertson BH, Galley HF, Ferguson NR, Webster NR (1998). The effect of nitric oxide and peroxynitrite on apoptosis in human polynorphonuclear leukocytes. Free Radic Biol Med.

[B267] Ward C, Wong TH, Murray J, Rahman I, Haslett C, Chilvers ER, Rossi AG (2000). Induction of human neutrophil apoptosis by nitric oxide donors: evidence for caspase-dependent, cyclic-GMP-independent, mechanism. Biochem Pharmacol.

[B268] Natarajan V, Taher MM, Roehm B, Parinandi NL, Schmid HH, Kiss Z, Garcia JG (1993). Activation of endothelial cell phospholipase D by hydrogen peroxide and fatty liver hydroperoxide. J Biol Chem.

[B269] Patel KD, Zimmerman GA, Prescott SM, McEver RP, McIntyre TM (1991). Oxygen radicals induce human endothelial cells to express GMP-140 and bind neutrophils. J Cell Biol.

[B270] Leonarduzzi G, Scavazza A, Biasi F, Chiarpotto E, Camandola S, Vogel S, Dargel R, Poli G (1997). The lipid peroxidation end product 4-hydroxy-2,3-nonenal up-regulates trasforming growth factor β1 expression in the macrophage lineage: a link between oxidative injury and fibrosclerosis. FASEB J.

[B271] Parola M, Leonarduzzi G, Biasi F, Albano E, Biocca ME, Poli G, Dianzani MU (1992). Vitamin E dietary supplementation protects against carbon tetrachloride-induced chronic liver damage and chirrosis. Hepatology.

[B272] Parola M, Muraca R, Dianzani I, Barrera G, Leonarduzzi G, Bendinelli P, Piccoletti R, Poli G (1992). Vitamin E dietary supplementation inhibits trasforming growth factor β1 gene expression in the rat liver. FEBS Lett.

[B273] Marra F, DeFranco R, Grappone C, Parola M, Milani S, Leonarduzzi G, Pastacaldi S, Wenzel UO, Pinzani M, Dianzani MU, Laffi G, Gentilini P (1999). Expression of monocyte chemotactic protein-1 precedes monocyte recruitment in a rat model of acute liver injury, and is modulated by vitamin E. J Investig Med.

[B274] Zamara E, Galastri S, Aleffi S, Petrai I, Aragno M, Mastrocola R, Novo E, Bertolani C, Milani S, Vizzutti F, Vercelli A, Pinzani M, Laffi G, LaVilla G, Parola M, Marra F (2007). Prevention of severe toxic liver injury and oxidative stress in MCP-1-deficient mice. J Hepatol.

[B275] Valente AJ, Graves DT, Vialle-Valentin CE, Delgado R, Schwartz CJ (1988). Purification of a monocyte chemotactic factor secreted by nonhuman primate vascular cells in culture. Biochemistry.

[B276] Loetscher P, Seitz M, Clark-Lewis I, Baggiolini M, Moser B (1994). Monocyte chemotactic proteins MCP-1, MCP-2, and MCP-3 are major attractants for human CD4+ and CD8+ T lymphocytes. FASEB J.

[B277] Rollins BJ (1996). Monocyte chemoattractant protein 1: a potential regulator of monocyte recruitment in inflammatory disease. Mol Med Today.

[B278] Gu L, Rutledge B, Fiorillo J, Ernst C, Grewal I, Flavell R, Glaude R, Rollins B (1997). *In vivo *properties of monocyte chemoattractant protein-1. J Leukoc Biol.

[B279] Shin WS, Szuba A, Rockson SG (2002). The role of chemokines in human cardiovascular pathology: enhanced biological insights. Atherosclerosis.

[B280] Daly C, Rollins BJ (2003). Monocyte chemoattractant protein-1 (CCL2) in inflammatory disease and adaptative immunity: therapeutic opportunities and controversies. Microcirculation.

[B281] Charo IF, Taubman MB (2004). Chemokines in the pathogenesis of vascular disease. Circ Res.

[B282] Marra F, Valente AJ, Pinzani M, Abboud HE (1993). Cultured human liver fat-storing cells produce monocyte chemotactic protein-1. Regulation by proinflammatory cytokines. J Clin Invest.

[B283] Czaja MJ, Geerts A, Xu J, Schmiedeberg P, Ju Y (1994). Monocyte chemoattractant protein 1 (MCP-1) expression occurs in toxic rat liver injury and human liver disease. J Leukocyte Biol.

[B284] Marra F, DeFranco R, Grappone C, Milani S, Pastacaldi S, Pinzani M, Romanelli RG, Laffi G, Gentilini P (1998). Increased expression of monocyte chemotactic protein-1 during active hepatic fibrogenesis: correlation with monocyte infiltration. Am J Pathol.

[B285] Marra F, Valente AJ, Grandaliano G, Abboud HE (1995). Thrombin stimulates proliferation of liver fat-storing cells and expression of monocyte chemotactic protein-1. Hepatology.

[B286] Xu Y, Rojkind M, Czaja MJ (1996). Regulation of monocyte chemoattractant protein 1 by cytokines and oxygen free radicals in rat hepatic fat-storing cells. Gastroenterology.

[B287] Roebuck KA, Carpenter LR, Lakshminarayanan V, Page SM, Moy JN, Thomas LL (1999). Stimulus-specific regulation of chemokine expression involves differential activation of the redox-responsive transcription factors AP-1 and NF-kappaB. J Leukoc Biol.

[B288] Parola M, Robino G, Marra F, Pinzani M, Bellomo G, Leonarduzzi G, Chiarugi P, Camandola S, Poli G, Waeg G, Gentilini P, Dianzani MU (1998). HNE interacts directly with JNK isoforms in human hepatic stellate cells. J Clin Invest.

[B289] Marinari UM, Nitti M, Pronzato MA, Domenicotti C (2003). Role of PKC-dependent pathways in HNE-induced cell protein transport and secretion. Mol Asp Med.

[B290] Marra F, Romanelli RG, Giannini C, Failli P, Pastacaldi S, Arrighi MC, Pinzani M, Laffi G, Montalto P, Gentilini P (1999). Monocyte chemotactic protein-1 as a chemoattractant for human hepatic stellate cells. Hepatology.

[B291] Mourelle M, Muriel P, Favari L, Franco T (1989). Prevention of CCl_4_-induced liver cirrhosis by silymarin. Fundam Clin Pharmacol.

[B292] Boigk G, Stroedter L, Herbst H, Waldschmidt J, Riecken EO, Schuppan D (1997). Silymarin retard collagen accumulation in early and advanced biliary fibrosis secondary to complete bile duct obliteration in rats. Hepatology.

[B293] Gasso M, Rubio M, Varala G, Cabre M, Cavalleria J, Alonso E, Deulofem R, Camps, Gimenez A, Pajares M, Pares A, Mato JM, Rodes J (1996). Effects of S-adenosylmethionine of lipid peroxidation and liver fibrogenesis in carbon tetrachloride-induced cirrhosis. J Hepatol.

[B294] Bedossa P, Houglum K, Trautwein C, Holstege A, Chojkier M (1994). Stimulation of collagen α _1_(I) gene expression is associated with lipid peroxidation in hepatocellular injury. A link to tissue fibrosis?. Hepatology.

[B295] Houglum K, Bedossa P, Chojkier M (1994). TGF-α and collagen α _1_(I) gene expression are increased in hepatic acinar zone I of rats with iron overload. Am J Physiol.

[B296] Niemela O, Parkkila S, Yla-Herttuala S, Villanueva J, Ruebner B, Halsted CH (1995). Sequential acetaldehyde production, lipid peroxidation and fibrogenesis in micropig model of alcohol induced disease. Hepatology.

[B297] Tsukamoto H, Horne W, Kamimura S, Niemela O, Parkkila S, Yla-Herttuala S, Brittenham GM (1995). Experimental liver cirrhosis induced by alcohol and iron. J Clin Invest.

[B298] Nanjii AA, Greenberg SS, Tahan SR, Fogt F, Loscalzo J, Sadrzadeh SM, Xie J, Stamler JS (1995). Nitric oxide production in experimental alcoholic liver disease in the rat: role in protection from injury. Gastroenterology.

[B299] Muriel P (1998). Nitric oxide protection of rat liver from lipid peroxidation, collagen accumulation, and liver damage induced by carbon tetrachloride. Biochem Pharmacol.

[B300] Parola M, Pinzani M, Casini A, Albano E, Poli G, Gentilini A, Gentilini P, Dianzani MU (1993). Stimulation of lipid peroxidation or 4-hydroxynonenal treatment increases procollagen α _1_(I) gene expression in human liver fat-storing cells. Biochem Biophys Res Commun.

[B301] Casini A, Ceni E, Salzano R, Biondi P, Parola M, Galli A, Foschi M, Caligiuri A, Pinzani M, Surrenti C (1997). Neutrophil-derived superoxide anion induces lipid peroxidation and stimulates collagen synthesis in human hepatic stellate cells: role of nitric oxide. Hepatology.

[B302] Maher JJ, Tzagarakis C, Gimenez A (1994). Malondialdehyde stimulates collagen production by hepatic lipocytes only upon activation in primary culture. Alcohol Alcohol.

[B303] Parola M, Pinzani M, Casini A, Leonarduzzi G, Marra F, Caligiuri A, Ceni E, Biondi P, Poli G, Dianzani MU (1996). Induction of procollagen type I gene expression and synthesis in human hepatic stellate cells by 4-hydroxy-2,3-nonenal and other 4-hydroxy-2,3-alkenalks is related to their molecular structure. Biochem Biophys Res Commun.

[B304] Svegliati Baroni G, D'Ambrosio L, Ferretti G, Casini A, Di Sario A, Salzano R, Ridolfi F, Saccomanno S, Jezequel AM, Benedetti A (1998). Fibrogenic effect of oxidative stress on rat hepatic stellate cells. Hepatology.

[B305] Garcia-Trevijano E, Iraburu MJ, Fontana L, Dominguez-Rosales JA, Auster A, Covarrubias-Pinedo A, Rojkind M (1999). Trasforming growth factor β1 induces the expression of α (I) procollagen mRNA by a hydrogen peroxide-C/EBPβ-dependent mechanism in rat hepatic stellate cells. Hepatology.

[B306] Nieto N, Friedman SL, Cederbaum AI (2002). Cytochrome P502E1-derived reactive oxygen species mediate paracrine stimulation of collagen I protein synthesis by hepatic stellate cells. J Biol Chem.

[B307] Nieto N, Cederbaum AI (2003). Increased Sp1-dependent transactivation of the LAMã promoter in hepatic stellate cells co-cultured with HepG2 cells overexpressing cytochrome P450 2E1. J Biol Chem.

[B308] Comporti M, Arezzini B, Signorini C, Sgherri C, Monaco B, Gardi C (2005). F2-isoprostanes stimulate collagen synthesis in activated hepatic stellate cells: a link with liver fibrosis?. Lab Invest.

[B309] Chen A, Davis BH (1999). UV irradiation activates JNK and increases alpha (I) collagen gene expression in rat hepatic stellate cells. J Biol Chem.

[B310] Nieto N, Friedman SL, Greenwel P, Cederbaum AI (1999). Cyp2E1-mediated oxidative stress induces collagen type I expression in rat hepatic stellate cells. Hepatology.

[B311] Nieto N, Greenwel P, Friedman SL, Zhang F, Dannenberg AJ, Cederbaum AI (2000). Ethanol and arachidonic acid increase α 2 (I) collagen expression in rat hepatic stellate cells overexpressing cytochrome P450 2E1. J Biol Chem.

[B312] De Bleser PJ, Xu G, Rombouts K, Rogiers V, Geerts A (1999). Glutathione levels discriminate between oxidative stress and transforming growth factor-β signalling in activated rat hepatic stellate cells. J Biol Chem.

[B313] Cao Q, Mak KM, Lieber CS (2002). DLPC decreases TGFβ1 – induced collagen mRNA by inhibiting p38 MAPK in hepatic stellate cells. Am J Physiol Gastrointest Liver Physiol.

[B314] Nieto N (2006). Oxidative-stress and IL-6 mediate the fibrogenic effects of Kupffer cells on stellate cells. Hepatology.

[B315] Greenwel P, Dominguez-Rosales JA, Mavi G, Rivas-Estilla AM, Rojkind M (2000). Hydrogen peroxide: a link between acetaldehyde-elicited alpha1(I) collagen gene up-regulation and oxidative stress in mouse hepatic stellate cells. Hepatology.

[B316] Svegliati-Baroni G, Inagaki Y, Rincon-Sanchez AR, Else C, Saccomanno S, Benedetti A, Ramirez F, Rojkind M (2005). Early response of alpha2(I) collagen to acetaldehyde in human hepatic stellate cells is TGF-beta independent. Hepatology.

[B317] Chen A, Davis BH (2000). The DNA binding protein BTEB mediates acetaldehyde-induced, Jun N-terminal kinase-dependent alphaI(I) collagen gene expression in rat hepatic stellate cells. Mol Cell Biol.

[B318] Bataller R, Schwabe RF, Choi YH, Yang L, Paik YH, Lindquist J, Qian T, Schoonhoven R, Hagedorn CH, Lemasters JJ, Brenner DA (2004). NADPH oxidase signal transduces angiotensin II in hepatic stellate cells and is critical in hepatic fibrosis. Hepatology.

[B319] Bataller R, Gabele E, Schoonhoven R, Morris T, Lehnert M, Yang L, Brenner DA, Rippe RA (2003). Prolonged infusion of angiotensin II into normal rats induces stellate cell activation and proinflammatory events in liver. Am J Physiol Gastrointest Liver Physiol.

[B320] Bataller R, Sancho-Bru P, Ginès p, Lora JM, Al-Garawi A, Solè M, Colmenero J, Nicolàs JM, Jimènez W, Weich N, Gutièrrez-Ramos JC, Arroyo V, Rodès J (2003). Activated human hepatic stellate cells express the renin-angiotensin system and synthesize angiotensin II. Gastroenterology.

[B321] Fadok VA, Bratton DL, Konowal A, Freed PW, Westcott JY, Henson PM (1998). Macrophages that have ingested apoptotic cells *in vitro *inhibit proinflammatory cytokine production through autocrine/paracrine mechanisms involving TGF-beta, PGE2, and PAF. J Clin Invest.

[B322] Zhan SS, Jiang JX, Wu J, Halsted C, Friedman SL, Zern MA, Torok NJ (2006). Phagocytosis of apoptotic bodies by hepatic stellate cells induces NADPH oxidase and is associated with liver fibrosis *in vivo*. Hepatology.

[B323] Buck M, Kim DJ, Houglum K, Hassanein T, Chojkier M (2000). C-Myb modulates transcription of the α-smooth muscle actin gene in activated hepatic stellate cells. Am J Physiol.

[B324] Nieto N, Friedman SL, Cederbaum AI (2002). Stimulation and proliferation of primary rat hepatic stellate cells by cytochrome P450 2E1-derived reactive oxygen species. Hepatology.

[B325] Kawada N, Seki S, Inoue M, Kuroki T (1998). Effect of antioxidants, resveratrol, quercetin and N-acetylcysteine, on the functions of cultured rat hepatic stellate cells and Kupffer cells. Hepatology.

[B326] Svegliati-Baroni G, Di Sario A, Casini A, Ferretti G, D'Ambrosio L, Ridolfi F, Bolognini L, Salzano R, Orlandi F, Benedetti A (1999). The Na^+^/H^+ ^exchanger modulates the fibrogenic effect of oxidative stress in rat hepatic stellate cells. J Hepatol.

[B327] Reeves HL, Dach CL, Peak M, Burt AD, Day CP (2000). Stress-activated protein kinases in the activation of rat hepatic stellate cells in culture. J Hepatol.

[B328] Benedetti A, Di Sario A, Casini A, Ridolfi F, Bendia E, Pigini P, Tonnini C, D'Ambrosio L, Feliciangeli G, Macarri G, Svegliati-Baroni G (2001). Inhibition of the Na^+^/H^+ ^exchanger reduces rat hepatic stellate cell activity and liver fibrosis: an *in vitro *and *in vivo *study. Gastroenterology.

[B329] Kim YK, Rhim TY, Choi I, Kim SS (2001). N-Acetylcysteine induces cell cycle arrest in hepatic stellate cells through its reducing activity. J Biol Chem.

[B330] Adachi T, Togashi H, Suzuki A, Kasai S, Ito J, Sugahara K, Kawata S (2005). NAD(P)H oxidase plays a crucial role in PDGF-induced proliferation of hepatic stellate cells. Hepatology.

[B331] Robino G, Zamara E, Novo E, Dianzani MU, Parola M (2001). 4-Hydroxy-2,3-alkenals as signal molecules modulating proliferative and adaptative cell responses. Biofactors.

[B332] Uchida K, Shiraichi M, Naito Y, Torii Y, Nakamura Y, Osawa T (1999). Activation of stress signaling pathways by the end product of lipid peroxidation. 4-Hydroxy-2-nonenal is a potential inducer of intracellular peroxide production. J Biol Chem.

[B333] Oleynyk JK, Khan NA, Ramm GA, Brown KE, O'Neill R, Britton RS, Bacon BR (2002). Aldehydic products of lipid peroxidation do not directly activate rat hepatic stellate cells. J Gastroenterol Hepatol.

[B334] Whalen R, Rockey DC, Friedman SL, Boyer TD (1999). Activation of rat hepatic stellate cells leads to loss of glutathione S-transferases and their enzymatic activity against products of oxidative stress. Hepatology.

[B335] Galli A, Svegliati-Baroni G, Ceni E, Milani S, Ridolfi F, Salzano R, Tarocchi M, Grappone C, Pellegrini G, Benedetti A, Surrenti C, Casini A (2005). Oxidative stress stimulates proliferation and invasiveness of hepatic stellate cells via a MMP2-mediated mechanism. Hepatology.

[B336] Novo E, Cannito S, Zamara E, Valfrè di Bonzo L, Tamagno E, Colombatto S, Marra F, Pinzani M, Parola M (2007). Cytokine-induced migration of human hepatic stellate cells requires activation of c-Jun N-terminal kinase and intracellular generation of reactive oxygen species. Gut.

[B337] Failli P, DeFranco R, Caligiuri A, Gentilini A, Romanelli RG, Marra F, Batignani G, Guerra CT, Laffi G, Gentilini P, Pinzani M (2000). Nitrovasodilators inhibit plateled derived growth factor-induced proliferation and migration of activated human hepatic stellate cells. Gastroenterology.

[B338] Klassen LW, Tuma D, Sorrell MF (1995). Immune mechanisms of alcohol-induced liver disease. Hepatology.

[B339] Albano E (2002). Free radical mechanisms in immune reactions associated with alcoholic liver disease. Free Radic Biol Med.

[B340] Mottaran E, Stewart SF, Rolla R, Vay D, Cipriani V, Moretti M, Vidali M, Sartori M, Rigamonti C, Day CP, Albano E (2002). Lipid peroxidation contributes to immune reactions associated with alcoholic liver disease. Free Radic Biol Med.

[B341] Thiele GM, Freeman TL, Klassen LW (2004). mmunologic mechanisms of alcoholic liver injury. Semin Liver Dis.

[B342] Rolla R, Vay D, Mottaran E, Parodi M, Traverso N, Aricó S, Sartori M, Bellomo G, Klassen LW, Thiele GM, Tuma DJ, Albano E (2000). Detection of circulating antibodies against malondialdehyde-acetaldehyde adducts in patients with alcohol-induced liver disease. Hepatology.

[B343] Stewart SF, Vidali M, Day CP, Albano E, Jones DE (2004). Oxidative stress as a trigger for cellular immune responses in patients with alcoholic liver disease. Hepatology.

[B344] Albano E, Mottaran E, Vidali M, Reale E, Saksena S, Occhino G, Burt AD, Day CP (2005). Immune response towards lipid peroxidation products as a predictor of progression of non-alcoholic fatty liver disease to advanced fibrosis. Gut.

[B345] Rigamonti C, Mottaran E, Reale E, Rolla R, Cipriani V, Capelli F, Boldorini R, Vidali M, Sartori M, Albano E (2003). Moderate alcohol consumption increases oxidative stress in patients with chronic hepatitis C. Hepatology.

[B346] Ronis MJ, Butura A, Korourian S, Shankar K, Simpson P, Badeaux J, Albano E, Ingelman-Sundberg M, Badger TM (2008). Cytokine and chemokine expression associated with steatohepatitis and hepatocyte proliferation in rats fed ethanol via total enteral nutrition. Exp Biol Med.

[B347] Tilg H, Moschen AR (2006). Adipocytokines: mediators linking adipose tissue, inflammation and immunity. Nat Rev Immunol.

[B348] Racanelli V, Rehermann B (2006). The liver as an immunological organ. Hepatology.

[B349] Winau F, Hegasy G, Weiskirchen R, Weber S, Cassan C, Sieling PA, Modlin RL, Liblau RS, Gressner AM, Kaufmann SH (2007). Ito cells are liver-resident antigen-presenting cells for activating T cell responses. Immunity.

[B350] Herkel J, Jagemann B, Wiegard C, Lazaro JF, Lueth S, Kanzler S, Blessing M, Schmitt E, Lohse AW (2003). MHC class II-expressing hepatocytes function as antigen-presenting cells and activate specific CD4 T lymphocyutes. Hepatology.

[B351] Ma X, Hua J, Mohamood AR, Hamad AR, Ravi R, Li Z (2007). A high-fat diet and regulatory T cells influence susceptibility to endotoxin-induced liver injury. Hepatology.

[B352] Pinzani M, Rombouts K, Colagrande S (2005). Fibrosis in chronic liver diseases: diagnosis and management. J Hepatol.

[B353] Chang CY, Argo CK, Al-Osaimi AMS, Caldwell SH (2006). Therapy of NAFLD: antioxidants and cytoprotective agents. J Clin Gastroenterol.

[B354] de Alwis NMW, Day CP (2008). Non-alcoholic fatty liver: the mist gradually clear. J Hepatol.

[B355] Williams RJ, Spencer JP, Rice-Evans C (2004). Flavonoids: antioxidants or signalling molecules?. Free Radic Biol Med.

[B356] Zhong Z, Froh M, Wheeler MD, Smutney O, Lehmann TG, Thurman RG (2002). Viral gene delivery of superoxide dismutase attenuates experimental cholestasis-induced liver fibrosis in the rat. Gene Ther.

[B357] Okuyama H, Nakamura H, Shimahara Y, Uyama N, Kwon YW, Kawada N, Yamaoka Y, Yodoi J (2005). Overexpression of thioredoxin prevents thioacetamide-induced hepatic fibrosis in mice. J Hepatol.

[B358] Tsui TY, Lau CK, Ma J, Wu X, Wang YQ, Farkas S, Xu R, Schlitt HJ, Fan ST (2005). rAAV-mediated stable expression of heme oxygenase-1 in stellate cells: a new approach to attenuate liver fibrosis in rats. Hepatology.

